# Recurrent pregnancy loss: systematic review and meta-analysis of overall prevalence and the distribution of major etiological categories

**DOI:** 10.3389/fmed.2026.1805994

**Published:** 2026-04-01

**Authors:** Tiago Carvalho, Miguel Ângelo-Dias, Filipa Moutinho, Sofia Silvério Serra, Teresa Costa, João Paulo Martins, Gonçalo S. Duarte, Jorge Lima

**Affiliations:** 1 Faculdade de Medicina da Universidade de Lisboa, Lisbon, Portugal; 2 Comprehensive Health Research Centre (CHRC), NOVA Medical School, Faculdade de Ciências Médicas, NMS, FCM, Universidade NOVA de Lisboa, Lisbon, Portugal; 3 Unidade Local de Saúde do Algarve, Faro, Portugal; 4 Library, NOVA Medical School, Faculdade de Ciências Médicas, NMS, FCM, Universidade NOVA de Lisboa, Lisbon, Portugal; 5 ESS, Polytechnic of Porto, Porto, Portugal; 6 Laboratory of Clinical Pharmacology and Therapeutics, Faculty of Medicine, University of Lisbon, Lisbon, Portugal; 7 Clinical Pharmacology Unit, Unidade Local de Saúde Santa Maria, Lisbon, Portugal; 8 Department of Obstetrics and Gynecology, Hospital da Luz Lisboa, Lisbon, Portugal

**Keywords:** recurrent pregnancy loss, prevalence, etiological factors, systematic review, meta-analysis

## Abstract

**Background:**

Recurrent pregnancy loss (RPL) is a clinically and emotionally significant reproductive condition, yet its reported prevalence and etiological distribution vary widely across studies. This systematic review and meta-analysis aimed to synthesize available evidence on the prevalence of RPL and the pooled proportions of its major etiological categories.

**Methods:**

We conducted a systematic review and meta-analysis of observational studies identified through searches of PubMed/Medline, EMBASE, Cochrane Library, Scopus, and Web of Science. Random-effects meta-analyses were performed to pool prevalence estimates and etiological proportions using inverse-variance weighting and a restricted maximum likelihood estimator. For prevalence analyses, the denominator corresponded to the total number of individuals screened, as reported by each study. Freeman–Tukey transformations were applied where appropriate. Heterogeneity was assessed using I^2^ and τ^2^.

**Results:**

A total of 105 studies were included, comprising 47,907 women with RPL for etiological analyses. Only two studies provided population-based prevalence estimates of RPL, yielding an estimated prevalence of approximately 1% (95% CI, 1–1%), although the small number of studies limits interpretation. Among women with RPL, the pooled proportion of idiopathic or unexplained RPL was highest (37, 95% CI, 30–44%; I^2^ = 94.3%), followed by acquired thrombophilia (12, 95% CI, 9–15%), endocrine factors (8, 95% CI, 6–10%), and anatomical factors and hereditary thrombophilia (6, 95% CI, 5–8%). Subgroup and meta-regression analyses suggested that geographic region and selected demographic and temporal study characteristics may contribute to between-study variability in etiological distributions.

**Conclusion:**

Reported prevalence and etiological proportions of RPL vary substantially across studies, and a large proportion of cases remain unexplained. The observed heterogeneity, partly associated with regional, demographic, and temporal factors, highlights the need for standardized definitions, diagnostic workups, and reporting practices to improve comparability across studies.

**Systematic review registration:**

PROSPERO Registry Number: CRD42024517675.

## Introduction

Recurrent pregnancy loss (RPL) is characterized by the occurrence of multiple pregnancy losses and represents a major clinical challenge associated with significant emotional distress for affected couples (1). Despite decades of research, substantial uncertainty remains regarding both its prevalence and etiological distribution.

Reported prevalence estimates of RPL vary widely, ranging from 0.6% to 5%, depending on population characteristics, diagnostic criteria, and study period (2–7). One of the earliest population-based studies, conducted by Alberman et al. (2), reported prevalence estimates of 1.5% and 0.8% among female doctors who had attempted pregnancy two or three times, respectively. Subsequent studies reported broadly similar estimates (3, 4, 7). However, much of these data are dated and may underestimate the true burden of RPL due to advances in early pregnancy detection and evolving diagnostic criteria. More recent reviews suggest that RPL affects approximately 1%–2% of women when defined as three consecutive pregnancy losses before 20 weeks of gestation (8).

Estimating the prevalence of RPL remains particularly challenging. In most countries, miscarriages and RPL are not systematically registered, and early pregnancy losses frequently go undocumented. Social and cultural stigma surrounding miscarriage may further contribute to underreporting in certain settings (9). In addition, the population at risk varies widely across studies, with denominators including women attempting conception, women with a history of pregnancy, or the general female population, thereby limiting comparability of prevalence estimates (10). These challenges are further enhanced by persistent heterogeneity in RPL definitions (10).

Indeed, there is no universal consensus regarding the definition of RPL across international guidelines. The World Health Organization originally defined RPL as three or more consecutive miscarriages before 22 weeks of gestation or fetal weight below 500 g (11). Subsequent definitions proposed by professional societies vary with respect to the number of losses required, gestational age thresholds, and inclusion of fetal weight, contributing further to inconsistencies across studies (10, 12–15).

Several established etiological categories of RPL have been described, including uterine anatomical abnormalities, inherited or acquired thrombophilia, endocrine disorders, and parental structural chromosomal abnormalities (16). In addition, maternal age, body weight, and lifestyle factors such as smoking, caffeine, and alcohol intake have been associated with an increased risk of pregnancy loss (10). Nevertheless, despite extensive evaluation, no identifiable cause is found, and a substantial proportion of cases remain unexplained (17, 18).

Although numerous studies have examined individual etiological categories, reported proportions vary considerably according to population demographics, diagnostic criteria, and extent of etiological workup. For example, parental chromosomal abnormalities are reported in 2%–5% of couples with RPL, compared with approximately 0.7% in the general population (19–23), while congenital uterine anomalies are more prevalent among women with pregnancy loss than among the general population (24, 25). Similarly, antiphospholipid syndrome represents a well-recognized acquired thrombophilia and a potentially treatable cause of RPL, although its reported prevalence varies across studies (26, 27). Considerable variability has also been reported for endocrine and infectious conditions, including chronic endometritis.

Understanding the prevalence of RPL and its major etiological categories is essential for reproductive health. Given the wide heterogeneity in reported prevalence estimates and etiological distributions, a comprehensive synthesis of the available evidence is warranted. Therefore, we conducted a systematic review and meta-analysis to estimate the prevalence of recurrent pregnancy loss and to quantify the pooled proportions of its established etiological categories.

## Materials and methods

### Study protocol

This systematic review and meta-analysis followed the Meta-Analysis of Observational Studies in Epidemiology (MOOSE) (28) and the Preferred Reporting Items for Systematic Reviews and Meta-Analysis (PRISMA) (29) guidelines (refer to Supplementary Table S1 for the PRISMA 2020 checklist) and was registered with PROSPERO (No: CRD42024517675) (30).

### Information sources and search strategy

A comprehensive literature review was conducted on January 15, 2025, across the major electronic databases: PubMed/Medline, Scopus, Web of Science, Cochrane Library, and EMBASE. The search strategy included terms related to RPL and prevalence (the full search strategies by database are presented in Supplementary Table S2). The literature review included studies published since 1995. References of the most relevant studies were hand-screened to identify any eventual missing publications not retrieved by the electronic search, and new searches were re-performed to ensure the inclusion of any eligible new publications during the conduction of this review.

### Eligibility criteria and study selection

Only observational studies reporting (i) the prevalence of RPL among adult women of reproductive age (≥18 years), diagnosed according to recognized clinical criteria as defined by the study authors, and/or (ii) the proportions of women with RPL with one or more established etiological categories were eligible. Established causes and their respective subtypes considered in this study included parental chromosomal abnormalities, uterine anatomical disorders, inherited and/or acquired thrombophilia, infectious causes, and endocrine disorders. Women with no identifiable cause, as determined by the diagnostic tests performed in each study and defined by the study authors, were classified as having idiopathic RPL. We included studies using either definition of RPL (two or more pregnancy losses or three or more pregnancy losses). Non-human studies were excluded.

We imposed no restrictions on the number of participants recruited, the number of recruitment centres, the regional area, or the language. Case reports (single), commentaries, letters to the editors, editorials, and review articles (wrong publication type) were excluded. Only studies published since 1995 were considered. Articles without enough data for analysis were also discarded.

Two reviewers (TC and MÂ-D) independently assessed all titles and abstracts of the retrieved search articles. The selection of full-text articles for inclusion was conducted independently by three reviewers (TC, MÂ-D, and FM), with any disagreements resolved by a fourth independent reviewer (JL).

The Rayyan® software was used to store, organize, and manage all the references obtained from the literature search (31).

### Data collection process and data items

From each study meeting the inclusion criteria, three reviewers (TC, MÂ-D, and FM) independently analysed and collected information on study authors, year of publication, study design, country of study, sample size, RPL definition applied, data on study population characteristics [including age, body mass index (BMI), the occurrence of previous miscarriages, ethnicity, the percentage of smoking women and the percentage of women consuming alcohol and caffeine]. The observed proportions of each established etiological category among women with RPL, together with their definitions and subtypes, were collected. Disagreements were resolved after discussion with a fourth reviewer (JL).

The primary outcomes were: (i) the prevalence of recurrent pregnancy loss (RPL), defined as the proportion of women meeting the study-specific definition of RPL (≥2 or ≥3 pregnancy losses) among the population assessed, using the denominator reported in each study; and (ii) the pooled proportions of established etiological categories among women with RPL, including parental chromosomal abnormalities, uterine anatomical disorders, inherited and/or acquired thrombophilia, endocrine disorders, and infectious causes. The secondary outcome was the pooled proportion of idiopathic/unexplained RPL, defined as the absence of an identifiable cause based on the diagnostic workup performed in each study.

### Risk of bias in the included studies

Two reviewers independently evaluated the methodological quality and risk of bias of the studies using the Joanna Briggs Institute (JBI) Critical Appraisal Checklist for Prevalence Studies (32). A third reviewer independently verified these evaluations. This tool comprises nine items addressing key domains (32). Each study was classified as having low risk of bias if all items were answered “Yes,” moderate risk if only one item was answered “No,” and high risk if two or more items were answered “No” (32).

### Data synthesis and statistical approach

Meta-analyses were conducted to estimate the overall prevalence of RPL and the pooled proportions of each etiological category among women with RPL. All pooled estimates and their 95% confidence intervals (CIs) were calculated using a random-effects model, employing the inverse variance method and the restricted maximum likelihood estimator.

For prevalence calculation, the total number of individuals screened was used as the denominator. Data was subjected to Freeman-Tukey transformation (double arcsine transformation) to avoid negative prevalence in the confidence interval (CI), limiting the CI between 0% and 100%. We used an Empirical Bayes estimator to pool data, and heterogeneity was examined using I^2^ and τ^2^ (tau-squared) (33). Subgroup analyses by continent of study and meta-regressions by age, BMI, and year of publication were performed according to the definitions of each RPL category. *p*-values were adjusted for multiple comparisons using the Holm method. When studies reported the age of participants as medians (with range or interquartile range), the sample mean was estimated using the methods described in (34).

Sensitivity analyses were performed for the proportions of the major categories of RPL by excluding studies rated as high risk of bias, or by excluding studies rated as either moderate or high risk of bias according to the JBI Critical Appraisal Checklist for Prevalence Studies.

## Results

### Study selection

**Figure 1:**
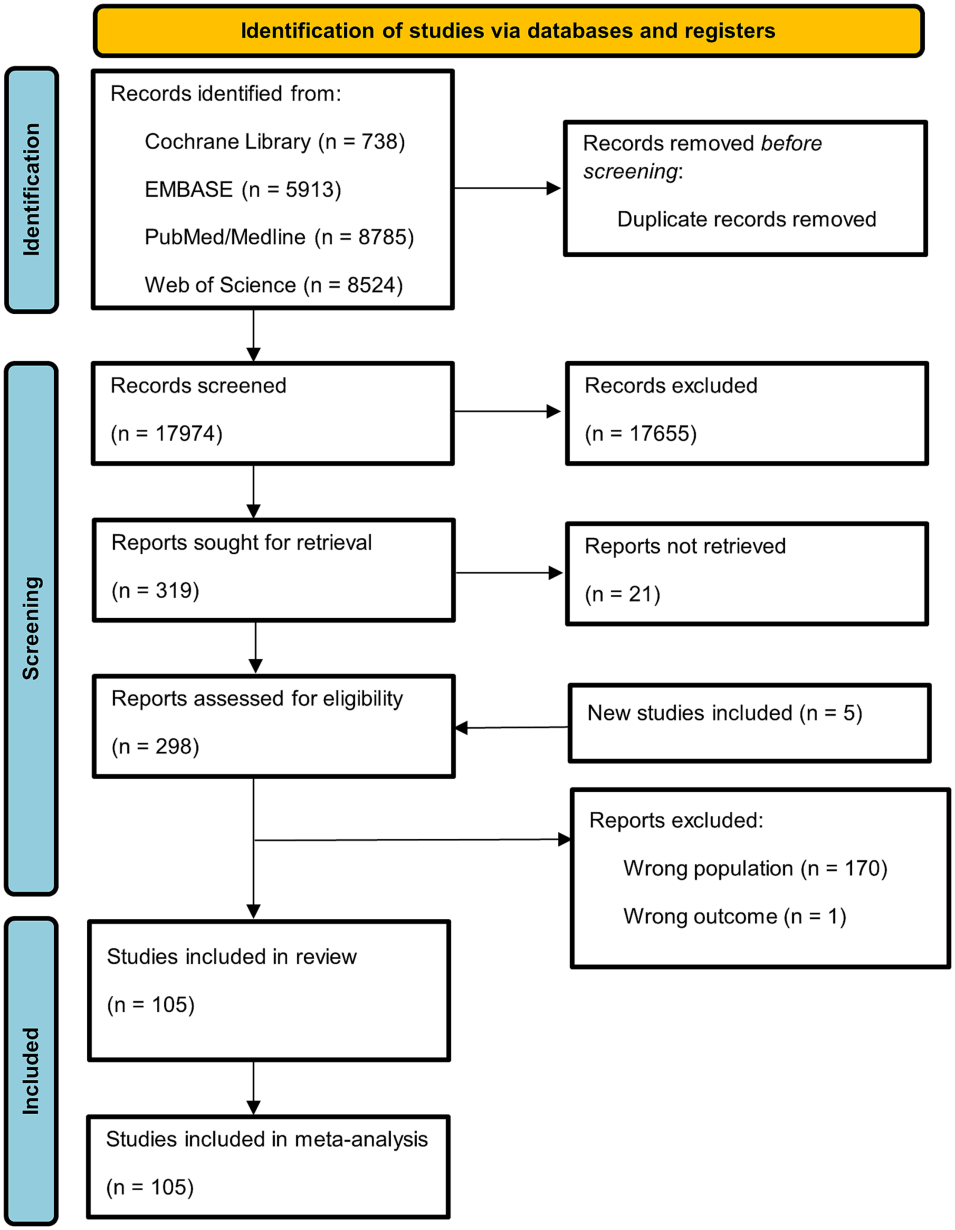
Preferred reporting items for systemic reviews and meta-analyses (PRISMA) 2020 flowchart.

Figure 1 depicts the PRISMA 2020 flow diagram of the systematic review process and study selection.

Our database search yielded 27,023 records. After removing 9,049 duplicates, 17,974 records were screened. Of these, 17,655 were excluded, and 319 reports were sought for retrieval. From these, 21 could not be retrieved and were excluded. After adding 5 new studies, we assessed 303 full-text reports for eligibility. After review, a total of 105 studies met all the inclusion criteria and were included in our systematic review (18, 25, 35–137) (Figure 1).

### Study characteristics

The characteristics of the included studies are detailed in Table 1.

**Table 1:** Characteristics of the included studies.

Study	Study type	Country of study	Sample size	RPL definition	Age^a^ (years)	BMI^a^ (kg/m^2^)	Previous Miscarriages^a^ (*n*)
Ahangari et al. 2019 (35)	Case–control study	Iran	245	≥2	32.2	NR	NR
Akbas et al. 2012 (36)	Cross-sectional cytogenetic study	Turkey	221 couples	≥2	NR	NR	NR
Ali et al. 2020 (37)	Prospective Cohort	India	280	≥2 consecutive losses, primary or secondary ≤ 24wg	30.5 ± 5	NR	NR
Arora et al. 2023 (38)	Observational study	India	65	≥2	27	NR	2,6
Awartani and Al Shabibi (39)	Retrospective cohort study	Saudi Arabia	1,074 couples	≥2	30.2 (female) 34.1 (male)	NR	5 ± 3
Bashiri et al. (40)	Retrospective cohort study	Israel	184	≥2 consecutive losses, primary	~27.5	NR	NR
Baumann et al. (41)	Retrospective cohort study	Germany	641	≥2 consecutive	33,2	NR	NR
Bedaiwy et al. (42)	Retrospective cohort study	Canada	591	≥2, <20 wg	36.0^b^ (range: 33.0–39.0)	24.0^b^ (range: 21.5–27.6)	NR
Bilibio et al. (43)	Cross-sectional study	Brazil	59	≥2, <12 wg	35.85	NR	NR
Bohlmann et al. (44)	Retrospective cohort study	Germany	206	≥2 consecutive losses, <14 wg	32.95 (2 miscarriages) 34.06 (≥3 miscarriages)	NR	NR
Bouet et al. (45)	Prospective observational study	Canada	53	≥2 consecutive losses, < 14 wg	34.5 ± 4.9	26.1 ± 4.7	NR
Bussen and Steck (46)	Case–control study	Germany	28	≥3, consecutive, primary or secondary	32.4 ± 4.5	NR	4.2 ± 1.2
Carbone et al. (47)	Retrospective cross-sectional study	Spain	333	≥2 consecutive	34	NR	2.8
Cavalcante et al. (48)	Multicenter, retrospective, observational study	Brazil	707 couples	≥2 consecutive losses, primary or secondary	32.5 (female) 36.2 (male)	NR	2.68 ± 0.9
Ching et al. (49)	Observational study	Malaysia	49	≥3 consecutive, ≤ 20 wg	30	NR	NR
Coccia et al. (50)	Retrospective cohort study	Italy	299 couples	≥2 consecutive 1st T losses	35.2 (female) 38.4 (male)	22.9 ± 3.3	NR
Coulam et al. (51)	Case–control genetic study	USA	150	≥2 consecutive	34.7	NR	NR
Coulam et al. (52)	Case–control genetic study	USA	550	≥2 consecutive	NR	NR	NR
Couto et al. (53)	Case–control study	Brazil	52	≥3 successive losses	NR	NR	3.75
Couto et al. (54)	Case–control study	Brazil	88	≥3	30.4	NR	3.6
Cueva et al. (55)	Observational cohort study	USA	74	≥2, <10 wg	35.5	26.7	NR
Dasari and Suganya (56)	Prospective observational study	India	323	≥2, wo prior birth in the index pregnancy	26.02 ± 4.12	NR	NR
Dendrinos et al. (57)	Prospective cohort study	Greece	48	>3 consecutive losses <20 wg	40.5 ± 5.2	NR	NR
Diejomaoh et al. (58)	Prospective observational study	Kuwait	90	≥3 consecutive losses	30.5 ± 6.0	NR	3.8
Diejomaoh et al. (59)	Prospective observational study	Kuwait	50	≥3 consecutive losses	33.8 ± 4.6	NR	NR
Drakeley et al. (60)	Retrospective cohort study	UK	158	≥2	32	NR	NR
Duzcan et al. (61)	Cross-sectional cytogenetic study	Turkey	113 couples	≥2 1^st^ T losses OR 1^st^ T abortion + 2nd and 3rd fetal death and/or malformed child	NR	NR	NR
Eltayeb et al. (62)	Retrospective cohort study	Oman	290	≥2 consecutive losses in 1^st^ or 2^nd^ T	32.93	NR	3^b^
Esplin et al. (63)	Case–control study	USA	74	≥3 consecutive losses, primary	33.6 ± 6.9	NR	4.5 ± 1.9
Fang et al. (64)	Retrospective cohort study	China	431	≥2 consecutive, <28 wg and HCG ≥ 50 IU/L	31,9	22.32 ± 3.11	2.6 ± 0.8
Foka et al. (65)	Case–control study	Greece	80	≥2	33	NR	3^b^
Gadwal and Harwal (66)	Descriptive, prospective observational study	India	60	≥2	NR	NR	NR
Ghosh et al. (67)	Prospective observational study	India	445	≥3 losses in 1^st^ T	NR	NR	NR
Godines-Enriquez et al. (68)	Retrospective cross-sectional	Mexico	203	≥ 2 losses, primary or secondary, < 24 wg	28.8 ± 4.6	27.7 ± 4.8	3.1 ± 0.8
Goncalves et al. (69)	Case–control study	Brazil	137	≥2 consecutive losses, < 12 wg	32.1	NR	2.8 ± 1.0
Goodman et al. (70)	Case–control genetic study	USA	550	≥2 consecutive losses	34.7	NR	NR
Guimaraes Filho et al. (71)	Retrospective cross-sectional study	Brazil	60	≥3 consecutive losses	32	NR	NR
Habayeb and Konje (72)	Retrospective observational study?	UK - England	189 couples	≥2 consecutive losses	33.3 (female) 35.4 (male)	NR	NR
Hassan et al. (73)	Case–control study	Iraq	55	≥2 consecutive losses, <24 wg	NR	NR	NR
Herzog et al. (74)	Retrospective observational study	Austria	207	≥3	33.4 ± 5.2	24.1 ± 4.4	NR
Huang et al. (75)	Case–control study	China	150	≥2 losses < 20 wg	30.2 ± 7.1	NR	2.6 ± 1.2
Iordache et al. (76)	Retrospective cohort study	Romania	211	≥2	NR	NR	NR
Isaoglu et al. (77)	Case–control study	Turkey	60	≥2 losses, primary or secondary	29.14 ± 6.18	NR	2.95 ± 1.65
Jaslow et al. (78)	Retrospective cohort study	USA	1,020	≥2 consecutive losses	32.6 ± 4.9	26.2 ± 6.3	3.4
Jaslow and Kutteh (35)	Single-centre, cross-sectional study	USA	875	≥2 consecutive losses	32.8 ± 4.8	NR	3.1 ± 1.2
Jivraj et al. (79)	Retrospective cohort study	UK	162	≥3	32.0 ± 5.4	NR	3.4
Kaider et al. (80)	Prospective immunodiagnostic study	USA	302	≥3 consecutive losses	NR	NR	NR
Karatas et al. (81)	Retrospective cohort study	Turkey	142 couples	≥2, <20 wg	29.8 (female) 32.2 (male)	NR	NR
Kasano et al. (82)	Retrospective observational study	Japan	828	≥2	38.19 ± 4.15	NR	2.37 ± 0,65
Kazerooni et al. (83)	Comparative case–control study	Iran	60	≥3 consecutive losses, < 20 wg	24.3 ± 4.8	28.3 ± 2.1	NR
Kocaaga et al. (84)	Retrospective descriptive study	Turkey	362 couples	≥2 losses in 1st T	29.1 (female) 32.4 (male)	NR	2^b^
Kovalak et al. (85)	Retrospective cohort	Turkey	75 couples	≥2 consecutive losses, < 20 wg	30 (female) 33 (male)	NR	NR
Kutteh et al. (86)	Prospective case–control study	USA	50	≥3 consecutive losses	33.6 ± 4.8	NR	4.1 ± 0.9
Kutteh et al. (87)	Retrospective two-center study	USA	700	≥2 consecutive losses	33.3 ± 5.0	NR	3.7 ± 1.6
Kutteh et al. (88)	Prospective cohort study	USA	65	≥2 consecutive losses	NR	NR	3^b^
Le et al. (89)	Cross-sectional study	Vietnam	301	≥2 consecutive losses	NR	NR	NR
Leduc-Robert et al. (90)	Retrospective cohort study	Canada	1,064	≥2 losses, < 20 wg	34.59 ± 4.64	24.89 ± 4.83	2.78
Lee et al. (91)	Retrospective observational study	South Korea	189	≥2 consecutive losses	32.4	NR	3.2
Lee et al. (92)	Prospective cohort study	South Korea	178	≥2	34.03 ± 4.30	NR	2.69 ± 1.11
Legnani et al. (93)	Case–control observational study	Italy	140	≥3 consecutive, or ≥2 with one euploid loss, <10 weeks	37.0 ± 5.6	NR	2.59 ± 0.79
Li et al. (94)	Retrospective observational study	UK	538	≥3 consecutive	32.4 ± 5.3	NR	4.3
Li et al. (95)	Retrospective cohort study	China	3,235 couples	≥2 losses < 20 wg	29.5 (female) 31.2 (male)	NR	2.4
Liu et al. (96)	Retrospective cohort study	China	232	≥2 losses during 1^st^ T	32.6 ± 4.8	NR	2.9 ± 1.3
Makino (97)	Cohort study	Japan	1,536 couples	≥2 losses	NR	NR	NR
Matjila et al. (98)	Retrospective analysis of prospective database	South Africa	592	≥3 consecutive (any trimester) or ≥2 consecutive second-trimester losses	29.7 ± 5.5	NR	3.34 ± 1.63
Mehta et al. (99)	Observational study	India	62	≥3 consecutive losses, < 20 wg	NR	NR	NR
Mishra et al. (100)	Observational study	India	112	≥2	28^b^	NR	NR
Morita et al. (101)	Prospective multicenter cohort study	Japan	1,340	≥2	35.1 ± 4.6	NR	2.8 ± 1.2
Niroumanesh et al. (103)	Descriptive cytogenetic study	Iran	100 couples	≥2 losses, < 20 wg	27.2 (female) 31.3 (male)	NR	2.7 ± 1.6
Nouri et al. (102)	Cross-sectional study	Iran	280	≥2, losses, < 20 wg	NR	NR	NR
Ocak et al. (104)	Case–control retrospective study	Turkey	495 couples	≥2 consecutive losses, < 20 wg	30.6	NR	NR
Oliveira et al. (105)	Retrospective cohort study	Brazil	127 couples	≥2 losses, < 20 wg	33.2 ± 5.7 (female) 35.3 ± 7.3 (male)	NR	3.1 ± 1.5
Oshiro et al. (106)	Retrospective cohort study	USA	366	≥2 losses	~32	NR	4.0^b^
Ozawa et al. (107)	Retrospective cohort study	Japan	2,324 couples	≥2 consecutive losses	NR	NR	NR
Pickering et al. (108)	Observational study	UK - England	122	≥3 consecutive losses	35^b^	NR	NR
Rai et al. (109)	Prospective cohort study	UK	1,111	≥3 consecutive losses, < 12 wg	34^b^	NR	3^b^
Rawat et al. (110)	Cross-sectional case–control study	India	100	≥2 losses	25.3 ± 3.7	NR	3.2
Roussev et al. (111)	Prospective immunologic study	USA	45	≥2 losses	NR	NR	NR
Sachdeva et al. (112)	Observational study	India	65	≥2 losses	NR	NR	NR
Salim et al. (113)	Prospective comparative study	UK	509	≥3 consecutive losses, < 14 wg	34.9	NR	4
Scarrone et al. (114)	Retrospective observational study	Italy	200	≥2 losses, < 20 wg	36.2	24.0 ± 3.9	3.4 ± 1.6
Singh (115)	Prospective observational study	India	150	≥3, < 20 wg	NR	NR	NR
Souza et al. (116)	Case–control study	Brazil	56	≥3	29.6	NR	NR
Souza et al. (117)	Cross-sectional study	Brazil	66	≥2 consecutive losses, <20 wg or fetal weight <500 g	34^b^	23.1^b^	3.0^b^
Stephenson (18)	Prospective cohort study	Canada	197 couples	≥3 consecutive losses, <20 wg, excluding aneuploidy	33	NR	4.1
Stern et al. (118)	Prospective prevalence study	Multinational	97	≥3 consecutive 1^st^ T losses	32.7	NR	4
Sugiura-Ogasawara et al. (119)	Observational cohort study	Japan	482	≥2 consecutive losses	32.4 ± 4.45	NR	3.04 ± 1.38
Tanimura et al. (120)	Prospective cross-sectional study	Japan	227	≥2 consecutive losses	35.0 ± 4.6	NR	3.6 ± 1.8
Ticconi et al. (121)	Prospective cohort study	Italy	431	≥ 2 losses, < 24 wg	35.83 ± 5.95	24.51 ± 4.66	NR
Ticconi et al. (122)	Retrospective observational study	Italy	843	≥ 2 losses, < 24 wg	35.6 ± 5.5	25.2 ± 5.7	2.81 ± 1.05
Triggianese et al. (123)	Cohort study	Italy	187	≥2 consecutive losses	37.1 ± 4.4	NR	2.6
Turki et al. (124)	Cross-sectional genetic study	Saudi Arabia	171 (73 couples + 25 women)	≥ 2 losses, < 20 wg	32.17 ± 6.39	NR	4.18 ± 2.58
Uysal et al. (125)	Cohort study	Turkey	57	≥3 consecutive losses, < 20 wg	30.6 ± 6.23	25.41 ± 4.34	3.98
Valli et al. (126)	Retrospective cohort study	Italy	344	≥2 losses in 1^st^ T	31.7 ± 4.9	NR	3.12 ± 1.05
Vaquero et al. (127)	Longitudinal case–control study	Italy	286 couples	≥2 losses, < 24 wg	37	23	3
Ventolini et al. (128)	Prospective cohort study	USA	23	≥3 unexplained 1^st^ or 2^nd^ T losses, primary	28.1	NR	3
Wagner et al. (129)	Retrospective cohort study	The Netherlands	173	≥3 consecutive losses <22 wg, with ≥1 previous birth ≥22 weeks.	29.7	25.1	NR
Westergaard et al. (130)	Cohort observational study	Denmark	10,691	≥3 consecutive losses	NR	NR	NR
Wolf et al. (131)	Cross-sectional study	Germany	49	≥2 unexplained early losses	31.9	NR	3
Wramsby et al. (132)	Prospective case–control study	Sweden	84	≥3 consecutive losses	NR	NR	NR
Yengel et al. (133)	Observational case–control study	Turkey	145	≥2 losses	30.5 ± 6.5	NR	NR
Yetman and Kutteh (134)	Retrospective study	USA	866	≥2 consecutive losses	NR	NR	NR
Yoshihara et al. (135)	Observational cohort study	Japan	1,014	≥2 losses	33.8	21	2.68
Yoshihara et al. (136)	Retrospective cohort study	Japan	1,021	≥2 losses	33^b^	20.4^b^	2^b^
Youssef et al. (137)	Retrospective cohort	Netherlands	383 couples	≥2 losses < 24 wg, excluding ectopic and molar pregnancies	32.1 (female) 34.2 (male)	25.1	NR

NR, not reported. Wg, weeks of gestation.

a Data are presented as mean with or without standard deviation (when provided), unless otherwise indicated.

b Data are presented as median.

The included articles were published between 1996 and 2025 and included a total of 47,907 women with RPL. Among 105 studies, 74 (70% of the sample, comprising 31,540 women with RPL) defined RPL as two or more pregnancy losses only, with a mean age of 32.41 years. The countries contributing the highest number of studies were the United States (15 studies, 14.3%), India (10 studies, 9.5%), Brazil (9 studies, 8.6%), and Turkey (9 studies, 8.6%). Additionally, 29 studies (28% of the sample, comprising 15,635 women with RPL) defined RPL as three or more pregnancy losses only, with a mean age of 32.11 years. Finally, two studies (encompassing a population of 732 women with RPL) investigated RPL applying both criteria depending on the trimester or the type of loss, with a mean age of 33.35 years (Table 1).

### Risk of bias of the included studies and sensitivity analysis

Of the 105 studies included, 83 (79.0%) were classified as having a low risk of bias, 18 (17.1%) as moderate risk, and only 4 (3.8%) as high risk of bias. These results indicate that most studies were of high methodological quality, according to JBI criteria (Table 2).

**Table 2:** Risk of bias assessment of the included studies using the Joanna Briggs Institute critical appraisal checklist for prevalence studies.

Study	Item 1	Item 2	Item 3	Item 4	Item 5	Item 6	Item 7	Item 8	Item 9	Overall risk of bias
Ahangari et al. (35)	Yes	Yes	Yes	Yes	Yes	Yes	Yes	Yes	Yes	Low
Akbas et al. (36)	Yes	Yes	Yes	Yes	Yes	Yes	Yes	Yes	Yes	Low
Ali et al. (37)	Yes	Yes	Yes	Yes	Yes	Yes	Yes	Yes	Yes	Low
Arora et al. (38)	Yes	Yes	Yes	No	Yes	Yes	Yes	Yes	Yes	Moderate
Awartani and Al Shabibi (39)	Yes	Yes	Yes	Yes	Yes	Yes	Yes	Yes	Yes	Low
Bashiri et al. (40)	Yes	Yes	Yes	Yes	Yes	Yes	Yes	Yes	Yes	Low
Baumann et al. (41)	Yes	Yes	Yes	Yes	Yes	Yes	Yes	Yes	Yes	Low
Bedaiwy et al. (42)	Yes	Yes	Yes	Yes	Yes	Yes	Yes	Yes	Yes	Low
Bilibio et al. (43)	Yes	Yes	Yes	No	No	Yes	Yes	Yes	Yes	High
Bohlmann et al. (44)	Yes	Yes	Yes	Yes	Yes	Yes	Yes	Yes	Yes	Low
Bouet et al. (45)	Yes	Yes	Yes	No	Yes	Yes	Yes	Yes	Yes	Moderate
Bussen and Steck (46)	Yes	Yes	No	Yes	Yes	Yes	Yes	Yes	Yes	Moderate
Carbone et al. (47)	Yes	Yes	Yes	Yes	Yes	Yes	Yes	Yes	Yes	Low
Cavalcante et al. (48)	Yes	Yes	Yes	Yes	Yes	Yes	Yes	Yes	Yes	Low
Ching et al. (49)	Yes	Yes	Yes	Yes	Yes	Yes	Yes	Yes	Yes	Low
Coccia et al. (50)	Yes	Yes	Yes	Yes	Yes	Yes	Yes	Yes	Yes	Low
Coulam et al. (51)	Yes	Yes	Yes	Yes	Yes	Yes	Yes	Yes	Yes	Low
Coulam et al. (52)	Yes	Yes	Yes	Yes	Yes	Yes	Yes	Yes	Yes	Low
Couto et al. (53)	Yes	Yes	Yes	Yes	Yes	Yes	Yes	Yes	Yes	Low
Couto et al. (54)	Yes	Yes	Yes	Yes	Yes	Yes	Yes	Yes	Yes	Low
Cueva et al. (55)	Yes	Yes	Yes	Yes	Yes	Yes	Yes	Yes	Yes	Low
Dasari and Suganya (56)	Yes	Yes	Yes	Yes	No	Yes	Yes	Yes	Yes	Moderate
Dendrinos et al. (57)	Yes	Yes	Yes	No	Yes	Yes	Yes	Yes	Yes	Low
Diejomaoh et al. (58)	Yes	Yes	Yes	Yes	Yes	Yes	Yes	Yes	Yes	Low
Diejomaoh et al. (59)	Yes	Yes	Yes	No	Yes	Yes	Yes	Yes	Yes	Moderate
Drakeley et al. (60)	Yes	Yes	Yes	Yes	Yes	Yes	Yes	Yes	Yes	Low
Duzcan et al. (61)	Yes	Yes	Yes	Yes	Yes	Yes	Yes	Yes	Yes	Low
Eltayeb et al. (62)	Yes	Yes	Yes	Yes	Yes	Yes	Yes	Yes	Yes	Low
Esplin et al. (63)	Yes	Yes	Yes	Yes	Yes	Yes	Yes	Yes	Yes	Low
Fang et al. (64)	Yes	Yes	Yes	Yes	Yes	Yes	Yes	Yes	Yes	Low
Foka et al. (65)	Yes	Yes	Yes	Yes	Yes	Yes	Yes	Yes	Yes	Low
Gadwal and Harwal (66)	Yes	Yes	Yes	Yes	Yes	Yes	Yes	Yes	Yes	Low
Ghosh et al. (67)	Yes	Yes	Yes	Yes	Yes	Yes	Yes	Yes	Yes	Low
Godines-Enriquez et al. (68)	Yes	Yes	Yes	Yes	Yes	Yes	Yes	Yes	Yes	Low
Goncalves et al. (69)	Yes	Yes	Yes	Yes	Yes	Yes	Yes	Yes	Yes	Low
Goodman et al. (70)	Yes	Yes	Yes	Yes	Yes	Yes	Yes	Yes	Yes	Low
Guimaraes Filho et al. (71)	Yes	Yes	Yes	No	Yes	Yes	Yes	Yes	Yes	Moderate
Habayeb and Konje (72)	Yes	Yes	Yes	Yes	Yes	Yes	Yes	Yes	Yes	Low
Hassan et al. (73)	Yes	Yes	Yes	No	Yes	Yes	Yes	Yes	Yes	Moderate
Herzog et al. (74)	Yes	Yes	No	Yes	No	Yes	Yes	Yes	Yes	High
Huang et al. (75)	Yes	Yes	Yes	Yes	Yes	Yes	Yes	Yes	Yes	Low
Iordache et al. (76)	Yes	Yes	Yes	Yes	Yes	Yes	Yes	Yes	Yes	Low
Isaoglu et al. (77)	Yes	Yes	Yes	No	Yes	Yes	Yes	Yes	Yes	Moderate
Jaslow et al. (78)	Yes	Yes	Yes	Yes	No	Yes	Yes	Yes	Yes	Moderate
Jaslow and Kutteh (35)	Yes	Yes	Yes	Yes	Yes	Yes	Yes	Yes	Yes	Low
Jivraj et al. (79)	Yes	Yes	Yes	No	Yes	Yes	Yes	Yes	Yes	Low
Kaider et al. (80)	Yes	Yes	Yes	Yes	Yes	Yes	Yes	Yes	Yes	Low
Karatas et al. (81)	Yes	Yes	Yes	Yes	Yes	Yes	Yes	Yes	Yes	Low
Kasano et al. (82)	Yes	Yes	Yes	Yes	Yes	Yes	Yes	Yes	Yes	Low
Kazerooni et al. (83)	Yes	Yes	Yes	Yes	Yes	Yes	Yes	Yes	Yes	Low
Kocaaga et al. (84)	Yes	Yes	Yes	Yes	Yes	Yes	Yes	Yes	Yes	Low
Kovalak et al. (85)	Yes	Yes	Yes	No	Yes	Yes	Yes	Yes	Yes	Moderate
Kutteh et al. (86)	Yes	Yes	Yes	No	Yes	Yes	Yes	Yes	Yes	Moderate
Kutteh et al. (87)	Yes	Yes	Yes	Yes	Yes	Yes	Yes	Yes	Yes	Low
Kutteh et al. (88)	Yes	Yes	Yes	Yes	Yes	Yes	Yes	Yes	Yes	Low
Le et al. (89)	Yes	Yes	Yes	Yes	No	Yes	Yes	Yes	Yes	Moderate
Leduc-Robert et al. (90)	Yes	Yes	Yes	Yes	Yes	Yes	Yes	Yes	Yes	Low
Lee et al. (91)	Yes	Yes	Yes	Yes	Yes	Yes	Yes	Yes	Yes	Low
Lee et al. (92)	Yes	Yes	Yes	Yes	Yes	Yes	Yes	Yes	Yes	Low
Legnani et al. (93)	Yes	Yes	Yes	Yes	Yes	Yes	Yes	Yes	Yes	Low
Li et al. (94)	Yes	Yes	Yes	Yes	Yes	Yes	Yes	Yes	Yes	Low
Li et al. (95)	Yes	Yes	Yes	Yes	Yes	Yes	Yes	Yes	Yes	Low
Liu et al. (96)	Yes	Yes	Yes	Yes	Yes	Yes	Yes	Yes	Yes	Low
Makino (97)	Yes	Yes	Yes	Yes	No	Yes	No	Yes	Yes	High
Matjila et al. (98)	Yes	Yes	Yes	Yes	Yes	Yes	Yes	Yes	Yes	Low
Mehta et al. (99)	Yes	Yes	Yes	Yes	No	No	Yes	Yes	Yes	High
Mishra et al. (100)	Yes	Yes	Yes	Yes	Yes	Yes	Yes	Yes	Yes	Low
Morita et al. (101)	Yes	Yes	Yes	Yes	Yes	Yes	Yes	Yes	Yes	Low
Niroumanesh et al. (103)	Yes	Yes	Yes	Yes	Yes	Yes	Yes	Yes	Yes	Low
Nouri et al. (102)	Yes	Yes	Yes	Yes	Yes	Yes	Yes	Yes	Yes	Low
Ocak et al. (104)	Yes	Yes	Yes	Yes	Yes	Yes	Yes	Yes	Yes	Low
Oliveira et al. (105)	Yes	Yes	Yes	Yes	Yes	Yes	Yes	Yes	Yes	Low
Oshiro et al. (106)	Yes	Yes	Yes	Yes	Yes	Yes	Yes	Yes	Yes	Low
Ozawa et al. (107)	Yes	Yes	Yes	Yes	Yes	Yes	Yes	Yes	Yes	Low
Pickering et al. (108)	Yes	Yes	Yes	Yes	Yes	Yes	Yes	Yes	Yes	Low
Rai et al. (109)	Yes	Yes	Yes	Yes	Yes	Yes	Yes	Yes	Yes	Low
Rawat et al. (110)	Yes	Yes	Yes	Yes	Yes	Yes	Yes	Yes	Yes	Low
Roussev et al. (111)	Yes	Yes	Yes	Yes	Yes	Yes	Yes	Yes	Yes	Low
Sachdeva et al. (112)	Yes	Yes	Yes	Yes	Yes	Yes	Yes	Yes	Yes	Low
Salim et al. (113)	Yes	Yes	Yes	Yes	Yes	Yes	Yes	Yes	Yes	Low
Scarrone et al. (114)	Yes	Yes	Yes	Yes	Yes	Yes	Yes	Yes	Yes	Low
Singh (115)	Yes	Yes	Yes	Yes	Yes	Yes	Yes	Yes	Yes	Low
Souza et al. (116)	Yes	Yes	Yes	Yes	Yes	Yes	Yes	Yes	Yes	Low
Souza et al. (117)	Yes	Yes	Yes	Yes	Yes	Yes	Yes	Yes	Yes	Low
Stephenson (18)	Yes	Yes	Yes	Yes	Yes	Yes	Yes	Yes	Yes	Low
Stern et al. (118)	Yes	Yes	Yes	Yes	No	Yes	Yes	Yes	Yes	Low
Sugiura-Ogasawara et al. (119)	Yes	Yes	Yes	Yes	Yes	Yes	Yes	Yes	Yes	Low
Tanimura et al. (120)	Yes	Yes	Yes	Yes	Yes	Yes	Yes	Yes	Yes	Low
Ticconi et al. (121)	Yes	Yes	Yes	Yes	Yes	Yes	Yes	Yes	Yes	Low
Ticconi et al. (122)	Yes	Yes	Yes	Yes	No	Yes	Yes	Yes	Yes	Moderate
Triggianese et al. (123)	Yes	Yes	Yes	Yes	Yes	Yes	Yes	Yes	Yes	Low
Turki et al. (124)	Yes	Yes	Yes	Yes	Yes	Yes	Yes	Yes	Yes	Low
Uysal et al. (125)	Yes	Yes	Yes	Yes	Yes	Yes	Yes	Yes	Yes	Low
Valli et al. (126)	Yes	Yes	Yes	Yes	Yes	Yes	Yes	Yes	Yes	Low
Vaquero et al. (127)	Yes	Yes	Yes	Yes	Yes	Yes	Yes	Yes	Yes	Low
Ventolini et al. (128)	Yes	Yes	Yes	No	Yes	Yes	Yes	Yes	Yes	Moderate
Wagner et al. (129)	Yes	Yes	Yes	Yes	Yes	Yes	Yes	Yes	Yes	Low
Westergaard et al. (130)	Yes	Yes	Yes	Yes	Yes	Yes	Yes	Yes	Yes	Low
Wolf et al. (131)	Yes	Yes	Yes	No	Yes	Yes	Yes	Yes	Yes	Moderate
Wramsby et al. (132)	Yes	Yes	Yes	No	Yes	Yes	Yes	Yes	Yes	Moderate
Yengel et al. (133)	Yes	Yes	Yes	No	Yes	Yes	Yes	Yes	Yes	Moderate
Yetman and Kutteh (134)	Yes	Yes	Yes	Yes	No	Yes	Yes	Yes	Yes	Moderate
Yoshihara et al. (135)	Yes	Yes	Yes	Yes	Yes	Yes	Yes	Yes	Yes	Low
Yoshihara et al. (136)	Yes	Yes	Yes	Yes	Yes	Yes	Yes	Yes	Yes	Low
Youssef et al. (137)	Yes	Yes	Yes	Yes	Yes	Yes	Yes	Yes	Yes	Low

When the analysis was repeated, excluding studies classified as having a high risk of bias, the estimates remained largely unchanged, with only a one percentage point difference observed for idiopathic RPL and acquired thrombophilia (Supplementary Figure S1). In contrast, after excluding studies rated as either moderate or high risk of bias, the largest change was observed for infectious causes, which increased by 2% (Supplementary Figure S2).

### Overall RPL prevalence

**Figure 2:**
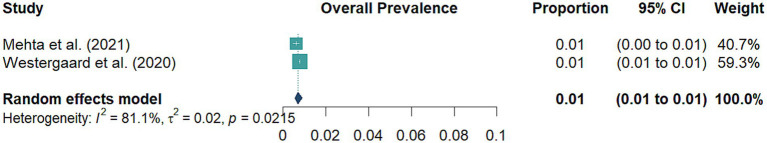
Forest plot showing the overall prevalence of recurrent pregnancy loss and 95% confidence intervals.

We pooled data from two studies, comprising a total of 1,385,460 participants, and calculated a prevalence of 1% (95% CI, 1%–1%; I^2^ = 81.1%; τ^2^ = 0.02) as illustrated in Figure 2 (see details in Supplementary Table S3).

Due to the limited number of studies, publication bias could not be assessed.

### Distribution of the major etiological categories in women with RPL

**Figure 3:**
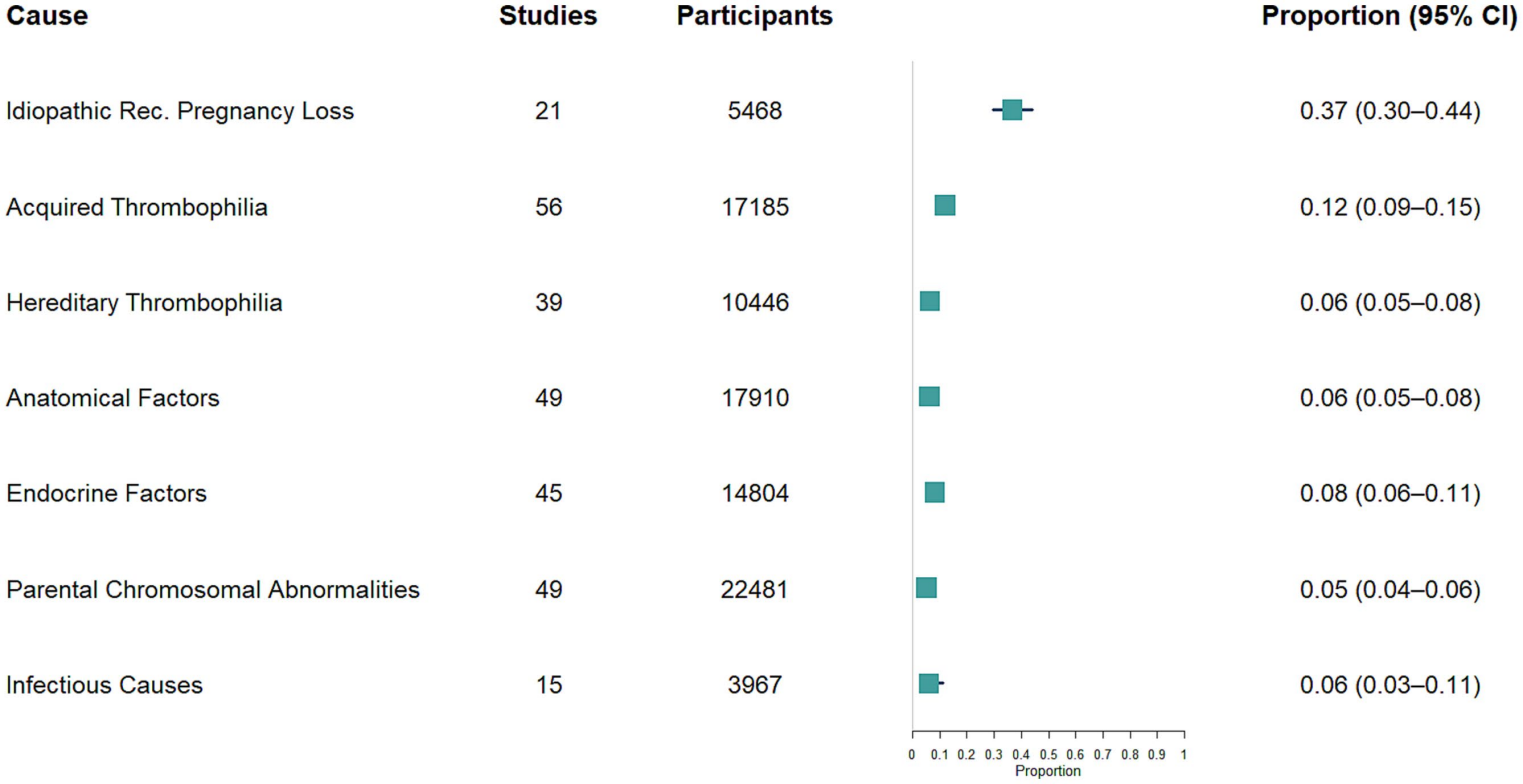
Forest plot showing the proportion of the major etiological categories of recurrent pregnancy loss and 95% confidence intervals.

Figure 3 summarises the overall distribution of pooled etiological proportions among women with RPL, based on data synthesised from multiple studies, highlighting the relative contribution of each factor and the associated 95% CIs.

#### Acquired thrombophilia

We pooled data from 56 studies, with a total of 17,185 participants. The estimated pooled proportion of acquired thrombophilia among women with RPL was 12% (95% CI, 9%–15%; I^2^ = 95.9%; τ^2^ = 1.15; Supplementary Figure S3).

The subgroup analysis according to the definition of acquired thrombophilia (classical antiphospholipid antibodies versus any antiphospholipid antibodies) did not reveal a statistically significant difference between subgroups (random effects model, *p*-value = 0.89; Supplementary Figure S3).

After imputing potentially missing studies with the trim-and-fill method, the pooled proportion estimate increased by 3%, indicating a possible impact of publication bias (Supplementary Figure S4).

#### Hereditary thrombophilia

We pooled data from 39 studies, with a total of 10,536 participants. The estimated pooled proportion of hereditary thrombophilia among women with RPL was 6% (95% CI, 5 to 8%; I^2^ = 95.3%; τ^2^ = 1.70; Supplementary Figure S5).

The subgroup analysis according to the definition of hereditary thrombophilia showed a statistically significant difference between subgroups (random effects model, *p*-value < 0.0001; Supplementary Figure S5). Detailed pairwise comparisons are provided in Supplementary Table S4.

The trim-and-fill analysis suggested that imputing potentially missing studies increased the pooled proportion estimate by 4% (Supplementary Figure S6).

#### Anatomical factors

We pooled data from 49 studies, with a total of 17,910 participants. The estimated pooled proportion of anatomical factors among women with RPL was 6% (95% CI, 5%–8%; I^2^ = 93.7%; τ^2^ = 1.08; Supplementary Figure S7).

A subgroup analysis was conducted based on the type of anatomical abnormality, namely, congenital uterine anomalies, acquired uterine anomalies, unspecified uterine anomalies, and cervical insufficiency. This analysis revealed a statistically significant difference between subgroups (random effects model, *p*-value = 0.0237; Supplementary Figure S7). Pairwise comparisons showed that cervical insufficiency differed significantly in distribution compared with acquired and unspecified uterine anomalies (*p*-value = 0.033 for both comparisons; Supplementary Table S5).

The trim-and-fill analysis suggested that imputing potentially missing studies increased the pooled proportion estimate by 3%, suggesting potential publication bias (Supplementary Figure S8).

#### Endocrine factors

We pooled data from 45 studies, with a total of 14,868 participants. The estimated pooled proportion of endocrine factors among women with RPL was 8% (95% CI, 6%–10%; I^2^ = 95.7%; τ^2^ = 1.74; Supplementary Figure S9).

A subgroup analysis based on the type of endocrine factor revealed a statistically significant difference between subgroups (random effects model, *p*-value < 0.0001, Supplementary Figure S9). Detailed pairwise comparisons are provided in Supplementary Table S6.

The trim-and-fill analysis suggested that imputing potentially missing studies increased the pooled prevalence estimate by 3% (Supplementary Figure S10).

#### Parental chromosomal abnormalities

We pooled data from 49 studies, with a total of 22,481 participants. The estimated pooled proportion of parental chromosomal abnormalities among couples with RPL was 5% (95% CI, 4%–6%; I^2^ = 93.0%; τ^2^ = 0.65; Supplementary Figure S11).

The trim-and-fill analysis suggested that imputing potentially missing studies increased the pooled proportion estimate by 1% (Supplementary Figure S12).

#### Infectious causes

We pooled data from 15 studies, with a total of 3,967 participants. The estimated pooled proportion of infectious causes among women with RPL was 6% (95% CI, 3%–11%; I^2^ = 89.7%; τ^2^ = 1.92; Supplementary Figure S13).

A subgroup analysis based on the type of infectious causes, including genital tract infections, bacterial vaginosis, chronic endometritis, and other infectious causes, did not show a statistically significant difference between the subgroups (random effects model, *p*-value = 0.3381, Supplementary Figure S13).

The trim-and-fill analysis suggested that imputing potentially missing studies increased the pooled prevalence estimate by 1% (Supplementary Figure S14).

#### Idiopathic RPL

We pooled data from 21 studies, with a total of 5,510 participants. The estimated pooled proportion of idiopathic (unexplained) RPL among women with RPL was 37% (95% CI, 30%–44%; I^2^ = 94.3%; τ^2^ = 0.48; Supplementary Figure S15). Only three studies distinguished idiopathic RPL cases according to whether products of conception were evaluated to exclude aneuploid losses. Subgroup analysis revealed a statistically significant difference between these groups (random-effects model, *p* = 0.001). The pooled proportion of idiopathic RPL was significantly lower in studies that excluded aneuploid losses and included only euploid losses (23, 95% CI 14%–34%; I^2^ = 32.7%; τ^2^ = 0.16) compared with studies using a classical idiopathic definition without products of conception analysis (39, 95% CI 32–47%; I^2^ = 93.9%; τ^2^ = 0.42). Details on the excluded and investigated causes applied by each study to classify RPL as idiopathic are provided in Supplementary Table S7.

No missing studies were identified using the trim and fill method (Supplementary Figure S16).

### Subgroup analysis by continent of study

To assess potential regional variation, we conducted subgroup analyses of the estimated proportion of each major etiological category of RPL, based on the continent in which each study was conducted (Supplementary Table S8).

A significant global subgroup effect by continent was observed for the proportion of acquired thrombophilia (*p* < 0.0001, Supplementary Table S8). Pairwise comparisons showed that the proportion of acquired thrombophilia was significantly higher in Oceania compared with Europe (*p* = 0.03, Supplementary Tables S9, S10).

For hereditary thrombophilia, we also observed a significant global subgroup effect (*p* < 0.0001, Supplementary Table S8). Pairwise analyses revealed that studies conducted in Africa reported significantly lower proportions of hereditary thrombophilia compared with all other regions (vs. Asia, Europe, and South America, *p* < 0.001; vs. North America, *p* = 0.01, Supplementary Tables S9, S10).

Regarding anatomical factors, several significant regional differences were observed (*p* < 0.0001, Supplementary Table S8). South America reported significantly higher proportions compared with Asia (*p* < 0.0001), Europe (*p* = 0.01), and Africa (*p* < 0.0001). Additionally, North America showed a higher proportion than Africa (*p* = 0.01), and a trend toward a higher proportion in South America compared with North America was also noted (*p* = 0.05) (Supplementary Tables S9, S10).

A significant global subgroup effect by continent was also observed for the proportions of idiopathic RPL (*p* < 0.0001; Supplementary Table S8). However, inspection of the effect estimates revealed no consistent differences between continents (Supplementary Tables S9, S10).

No significant regional differences were observed for other major etiological categories included in this study (Supplementary Tables S8–S10).

### Impact of age, BMI, and year of publication

Meta-regression analyses were performed to assess whether mean maternal age, BMI, or year of publication influenced the reported proportion of the major etiological category of RPL.

No significant associations were found between maternal age and any etiological category of RPL (Supplementary Table S11; Supplementary Figures S17–S23).

BMI was positively associated with the prevalence of acquired thrombophilia (slope = 0.168, 95% CI 0.004 to 0.332, *p* = 0.045), suggesting that higher BMI may be linked to increased reporting or diagnosis of acquired thrombophilia in RPL populations (Supplementary Table S12; Supplementary Figures S24–S28).

Year of publication was negatively associated with the distribution of acquired thrombophilia (slope = −0.046, *p* = 0.0001) and anatomical factors (slope = −0.031, *p* = 0.02), indicating a decreasing trend in reported proportions of these etiological categories over time (Supplementary Table S13; Supplementary Figures S29–S35).

No other significant associations were identified (Supplementary Tables S11–S13).

## Discussion

Our meta-analysis provides updated estimates of both the overall prevalence of RPL and the pooled proportion of the major etiological categories identified among women with RPL. These findings, synthesised from a comprehensive review and analysis of the existing literature, offer valuable insights into the multifactorial nature of RPL and the diverse array of underlying conditions associated with recurrent miscarriages.

Evidence supports substantial variability in reported estimates of the overall prevalence of RPL, ranging from 0.6% to 5% across different studies (2–7). Our findings fall within this range, with an estimated pooled prevalence of RPL of 1%. However, this estimate should be interpreted cautiously, as only two population-based studies were available, and denominators varied across the literature. Nevertheless, estimating the prevalence of RPL remains clinically relevant, as it highlights the overall burden of this condition and underscores its impact on reproductive health (138). RPL imposes significant stress on both the physical and mental health of affected women, frequently requiring healthcare interventions, incurring substantial expenses, and causing significant emotional distress (139). Improved prevalence estimates may assist healthcare professionals and policymakers in supporting women in managing reproductive health challenges associated with RPL (140, 141).

We also quantified the pooled proportions of idiopathic RPL and several established etiological categories among women with RPL, including thrombophilias (both acquired and hereditary), anatomical factors, parental chromosomal abnormalities, endocrine factors and infectious causes.

Regarding acquired thrombophilia, antiphospholipid syndrome (APS) represents the predominant condition and is characterised by classical antibodies such as lupus anticoagulant, anticardiolipin antibodies, and anti-beta-2-glycoprotein I antibodies, as well as other non-classical antibodies (12). Given that APS represents a potentially treatable condition, current guidelines recommend that APS should be considered in the diagnostic workup of women with RPL (26). In clinical practice, the American College of Obstetricians and Gynecologists (ACOG) recommend testing patients with RPL for classical APS antibodies but not routinely for other antiphospholipid antibodies. Furthermore, treatment is recommended only for women diagnosed with APS rather than for those without confirmed APS (17). In our meta-analysis, we considered both classical and other antiphospholipid antibodies.

The pooled proportions of acquired thrombophilia among women with RPL was 12%. We also conducted a subgroup analysis comparing classical antiphospholipid antibodies versus any antiphospholipid antibodies, but no statistically significant differences were observed. Antiphospholipid antibodies have been reported in approximately 1%–5% of the general healthy population (142). Our estimate is broadly consistent with prior literature suggesting that APS may be present in higher proportions in women with RPL (26, 27). Nevertheless, some studies have reported similar APS prevalence in women with RPL and in the general population, questioning the magnitude of this association (143). These discrepancies may partly reflect heterogeneity in diagnostic criteria, as APS definitions have evolved (e.g., Sapporo versus Sydney criteria), as well as differences in the antibody panels assessed and the clinical thresholds applied across studies. In line with this, our meta-regression showed a negative association between year of publication and the reported proportion of acquired thrombophilia, suggesting that changes in diagnostic practices and criteria over time may influence observed estimates and potentially bring them closer to the prevalence ranges reported in healthy populations.

In the context of hereditary thrombophilias, we considered the Factor V Leiden mutation, prothrombin gene mutation, antithrombin III deficiency, methylenetetrahydrofolate reductase (MTHFR) mutation, protein C deficiency, and protein S deficiency (12). Current guidelines from the RCOG advise against routine testing for protein C deficiency, antithrombin deficiency, and MTHFR mutation, as systematic reviews and meta-analyses have not consistently demonstrated strong associations with RPL (12, 144). The ACOG recommendations similarly discourage broad thrombophilia screening in the absence of additional clinical indications (17). Our pooled analysis suggested that the proportion of hereditary thrombophilia among women with RPL was approximately 6%. However, definitions and diagnostic panels varied widely across studies, likely contributing to the substantial heterogeneity observed.

Regarding anatomical structural abnormalities, such as congenital uterine anomalies (septate uterus, arcuate uterus, bicornuate uterus, unicornis uterus, and double uterus), and acquired uterine structural abnormalities (intrauterine adhesion, hysteromyoma, and adenomyosis), the existing literature has reported relatively high (16%) prevalence estimates among women with RPL (145). In our meta-analyses, the pooled proportion of uterine anatomical abnormalities among women with RPL was 6%, including both congenital and acquired structural abnormalities. Differences between our pooled estimate and higher figures reported in previous literature may reflect variation in diagnostic modalities (e.g., routine 3D ultrasound versus selective investigation), inclusion or exclusion of minor anomalies, and heterogeneous classification systems across studies. Given that uterine abnormalities represent potentially correctable conditions, RCOG guidelines recommend assessment of uterine anatomy, including evaluation with 3D ultrasound, as part of the investigation of RPL (12). Similarly, ACOG recommends evaluation of the uterine cavity within the diagnostic workup for RPL (17).

Endocrine factors relevant to RPL include polycystic ovary syndrome (PCOS), prolactin imbalances, thyroid antibodies, luteal phase defect, diabetes mellitus, thyroid disease, and subclinical hypothyroidism (12). According to the RCOG, increased risk of RPL is associated with subclinical hypothyroidism, thyroid antibodies, PCOS, and prolactin imbalances, whereas evidence remains insufficient for well-controlled diabetes, thyroid disease, or luteal phase defect (146). The RCOG recommends testing thyroid function and thyroid peroxidase antibodies (12), and ACOG similarly recommends thyroid screening and investigation of PCOS or insulin resistance only when clinically indicated (17). The pooled proportion of endocrine factors among women with RPL in our analysis was 8%. Reported estimates in the literature vary substantially, with hypothyroidism prevalence around 4% (147) and subclinical hypothyroidism prevalence reported up to 19% in women with RPL (148). The prevalence of PCOS appears to be similar in women with RPL and the general population, and it does not seem to affect the prognosis of RPL (20). In addition, a study by Zhang et al. (149) estimated a prevalence of RPL associated with abnormal endocrine test results of 51.8%. However, abnormal endocrine test results do not necessarily indicate clinically significant dysfunction, and differences in diagnostic thresholds and confounding factors may contribute to variability across studies it is important to note that abnormal test results do not always indicate underlying endocrine dysfunctions. Additionally, the authors highlight confounding factors that may have led to an overestimation of the true prevalence of this condition. This emphasises the need for standardised definitions and robust prospective research in this area.

Regarding chromosomal factors, both parental chromosome abnormalities and foetal chromosomal abnormalities are important considerations (12). The ACOG suggests parental karyotyping, particularly in cases with known translocations or previous unbalanced chromosomal results, and supports genetic testing of products of conception in selected cases of recurrent losses (17). In our meta-analysis, the pooled proportion of parental chromosomal abnormalities among couples with RPL was 5%, aligning with previous reports of 2%–5% in RPL populations compared with approximately 0.7% in the general population (19–23).

Regarding infectious causes, the literature has primarily focused on chronic endometritis and genital tract infections. TORCH infections (toxoplasmosis, rubella, cytomegalovirus, herpes simplex), and listeria are generally excluded from consideration, as they do not represent persistent genital tract infections sufficient to evade detection or induce significant symptoms in women with RPL (12). Current guidelines do not recommend routine infectious screening in women with RPL due to limited evidence supporting the benefit (12, 17). In our meta-analysis, the pooled proportion of infectious causes among women with RPL was 6%. Estimates varied widely across studies, with reported prevalence of chronic endometritis ranging from 9% to 56% (9). Evidence in this area remains limited, highlighting the need for prospective studies using standardised diagnostic criteria (150). Investigating the potential impact of primary prevention measures within primary health care services on the prevalence of infection as a cause of RPL is of particular interest. It is anticipated that such interventions may lead to a reduction in preventable infections associated with RPL.

Idiopathic or unexplained RPL refers to cases in which no specific cause is identified after diagnostic evaluation (17). We estimated that 37% of women with RPL were classified as idiopathic, consistent with prior literature suggesting that up to 50% of cases remain unexplained (18, 151). Idiopathic RPL remains the most frequent category largely because it represents a diagnosis of exclusion, reflecting persistent gaps in the understanding of RPL pathophysiology (145). Moreover, idiopathic classification depends heavily on the extent of etiological workup performed, which varies substantially across studies. As our analysis included studies published between 1995 and 2025, earlier studies may have classified cases as idiopathic that would likely be attributed to identifiable causes today. This temporal factor may partially contribute to variability in reported proportions (152), although in our meta-regression, year of publication was not significantly associated with the pooled proportion of idiopathic RPL. Importantly, although genetic analysis of products of conception (POC) was not a predefined inclusion or exclusion criterion in this review, most included studies did not report performing such analyses. Given that fetal chromosomal abnormalities are a major cause of pregnancy loss and may account for a substantial proportion of recurrent miscarriages (119), the absence of POC testing may have resulted in misclassification of some cases as unexplained and contributed to an overestimation of idiopathic RPL. It should also be noted that, while genetic testing of POC can help clarify the cause of an individual pregnancy loss, its role in routine RPL workup remains variable across settings, partly due to cost, availability, and limited implications for modifiable management strategies in some cases (17). In our dataset, only three studies distinguished idiopathic RPL according to whether genetic analysis of products of conception (POC) was performed. Subgroup analysis showed a statistically significant difference between these studies and those not including POC evaluation, with a substantially lower pooled proportion of idiopathic RPL when aneuploid losses were excluded (23% vs. 39%). This finding suggests that the absence of POC analysis may contribute to the misclassification of cases as idiopathic and to an overestimation of unexplained RPL. Nevertheless, this result should be interpreted cautiously, given the small number of studies contributing to this subgroup analysis.

Overall, our systematic review and meta-analysis included 105 observational studies from diverse geographic regions, providing a comprehensive global perspective on RPL. Subgroup analyses indicated potential differences across continents. However, given that some continents were represented by only a small number of studies, these findings should be interpreted as exploratory. Meta-regression analyses suggested that BMI and year of publication influenced reported proportions of certain etiological categories, underscoring the importance of considering demographic and temporal factors when interpreting global data.

This study had several strengths. Pooled proportion estimates for the major etiological categories were overall consistent in sensitivity analyses excluding studies at high risk of bias or excluding studies at moderate and high risk of bias, strengthening confidence in our findings. Grouping specific diagnoses into broader etiological categories provided a clearer overview of RPL, facilitating interpretation of the literature. Additionally, the wide geographic coverage enhances the generalizability of findings across diverse populations and healthcare settings.

This study also had limitations, including the substantial heterogeneity observed across all pooled analyses. Furthermore, only two studies provided population-based prevalence estimates of RPL, comprising 1,385,460 screened participants, which limits the robustness of pooled prevalence estimates despite the large denominator. The small number of studies prevented further exploration of prevalence differences by RPL definition (≥2 versus ≥3 losses) or by demographic and regional factors, highlighting the need for additional population-based observational research. It should be highlighted that possible publication bias was observed for the prevalences of acquired thrombophilia, hereditary thrombophilia, anatomical factors, endocrine factors, chromosomal anomalies, and infectious causes, with an underrepresentation of studies reporting higher prevalences of these etiological categories of RPL.

A meta-analysis published by van Dijk et al. (153) also examined RPL-related etiologies. Their work focused on comparing etiological frequencies between definitions of RPL based on ≥2 versus ≥3 pregnancy losses, using odds ratios to evaluate diagnostic timing (153). In contrast, our study aimed to provide pooled descriptive estimates of RPL prevalence and etiological proportions across a broad global literature, without comparative analyses based on the number of losses.

In conclusion, our study provides updated pooled estimates of both the overall prevalence of RPL and the major etiological categories reported among women with RPL. We observed regional variation in certain etiological categories, particularly acquired and hereditary thrombophilia, while BMI and publication year also influenced reported proportions. A substantial proportion of cases remain unexplained, underscoring persistent knowledge gaps and the need for standardised definitions, diagnostic workups, and reporting practices. Further population-based studies and prospective investigations are required to refine prevalence estimates, improve etiological classification, and ultimately enhance patient care.

## Data Availability

The original contributions presented in the study are included in the article/Supplementary material, and further inquiries can be directed to the corresponding authors.

## Supplementary material

SUPPLEMENTARY FIGURE S1 Sensitivity analyses for the proportion of the major etiological categories of recurrent pregnancy loss, by excluding studies rated as high risk of bias.

SUPPLEMENTARY FIGURE S2 Sensitivity analyses for the proportion of the major etiological categories of recurrent pregnancy loss, by excluding studies rated as either moderate or high risk of bias.

SUPPLEMENTARY FIGURE S3 Forest plot showing the proportion of acquired thrombophilia among women with recurrent pregnancy loss and 95% confidence intervals.

SUPPLEMENTARY FIGURE S4 Funnel plot for acquired thrombophilia. Observed studies are shown as black dots and open red circles represent studies imputed by the trim-and-fill algorithm. The vertical dashed line indicates the pooled mean difference. The blue lines represent pseudo 95% confidence limits.

SUPPLEMENTARY FIGURE S5 Forest plot showing the proportion of hereditary thrombophilia among women with recurrent pregnancy loss and 95% confidence intervals.

SUPPLEMENTARY FIGURE S6 Funnel plot for hereditary thrombophilia. Observed studies are shown as black dots and open red circles represent studies imputed by the trim-and-fill algorithm. The vertical dashed line indicates the pooled mean difference. The blue lines represent pseudo 95% confidence limits.

SUPPLEMENTARY FIGURE S7 Forest plot showing the proportion of anatomical factors among women with recurrent pregnancy loss and 95% confidence intervals.

SUPPLEMENTARY FIGURE S8 Funnel plot for anatomical factors. Observed studies are shown as black dots and open red circles represent studies imputed by the trim-and-fill algorithm. The vertical dashed line indicates the pooled mean difference. The blue lines represent pseudo 95% confidence limits.

SUPPLEMENTARY FIGURE S9 Forest plot showing the proportion of endocrine factors among women with recurrent pregnancy loss and 95% confidence intervals.

SUPPLEMENTARY FIGURE S10 Funnel plot for endocrine factors. Observed studies are shown as black dots and open red circles represent studies imputed by the trim-and-fill algorithm. The vertical dashed line indicates the pooled mean difference. The blue lines represent pseudo 95% confidence limits.

SUPPLEMENTARY FIGURE S11 Forest plot showing the proportion of parental chromosomal abnormalities among women with recurrent pregnancy loss and 95% confidence intervals.

SUPPLEMENTARY FIGURE S12 Funnel plot for parental chromosomal abnormalities. Observed studies are shown as black dots and open red circles represent studies imputed by the trim-and-fill algorithm. The vertical dashed line indicates the pooled mean difference. The blue lines represent pseudo 95% confidence limits.

SUPPLEMENTARY FIGURE S13 Forest plot showing the proportion of infectious causes among women with recurrent pregnancy loss and 95% confidence intervals.

SUPPLEMENTARY FIGURE S14 Funnel plot for infectious causes. Observed studies are shown as black dots and open red circles represent studies imputed by the trim-and-fill algorithm. The vertical dashed line indicates the pooled mean difference. The blue lines represent pseudo 95% confidence limits.

SUPPLEMENTARY FIGURE S15 Forest plot showing the proportion of idiopathic recurrent pregnancy loss among women with recurrent pregnancy loss and 95% confidence intervals.

SUPPLEMENTARY FIGURE S16 Funnel plot for idiopathic recurrent pregnancy loss. Observed studies are shown as black dots and open red circles represent studies imputed by the trim-and-fill algorithm. The vertical dashed line indicates the pooled mean difference. The blue lines represent pseudo 95% confidence limits.

SUPPLEMENTARY FIGURE S17 Meta-regression analysis by age for acquired thrombophilia.

SUPPLEMENTARY FIGURE S18 Meta-regression analysis by age for hereditary thrombophilia.

SUPPLEMENTARY FIGURE S19 Meta-regression analysis by age anatomical factors.

SUPPLEMENTARY FIGURE S20 Meta-regression analysis by age for endocrine factors.

SUPPLEMENTARY FIGURE S21 Meta-regression analysis by age for parental chromosomal abnormalities.

SUPPLEMENTARY FIGURE S22 Meta-regression analysis by age for infectious causes.

SUPPLEMENTARY FIGURE S23 Meta-regression analysis by age for idiopathic recurrent pregnancy loss.

SUPPLEMENTARY FIGURE S24 Meta-regression analysis by body mass index for acquired thrombophilia.

SUPPLEMENTARY FIGURE S25 Meta-regression analysis by body mass index for hereditary thrombophilia.

SUPPLEMENTARY FIGURE S26 Meta-regression analysis by body mass index for anatomical factors.

SUPPLEMENTARY FIGURE S27 Meta-regression analysis by body mass index for endocrine factors.

SUPPLEMENTARY FIGURE S28 Meta-regression analysis by body mass index for parental chromosomal abnormalities.

SUPPLEMENTARY FIGURE S29 Meta-regression analysis by year of publication for acquired thrombophilia.

SUPPLEMENTARY FIGURE S30 Meta-regression analysis by year of publication for hereditary thrombophilia.

SUPPLEMENTARY FIGURE S31 Meta-regression analysis by year of publication for anatomical factors.

SUPPLEMENTARY FIGURE S32 Meta-regression analysis by year of publication for endocrine factors.

SUPPLEMENTARY FIGURE S33 Meta-regression analysis by year of publication for parental chromosomal abnormalities.

SUPPLEMENTARY FIGURE S34 Meta-regression analysis by year of publication for infectious causes.

SUPPLEMENTARY FIGURE S35 Meta-regression analysis by year of publication for idiopathic recurrent pregnancy loss.
