# Supplementary figures and images for: Recurrent pregnancy loss: systematic review and meta-analysis of overall prevalence and the distribution of major etiological categories
Source: Front Med (Lausanne). 2026 Apr 1;13:1805994. doi: 10.3389/fmed.2026.1805994 (PMC13079578; doi:10.3389/fmed.2026.1805994)

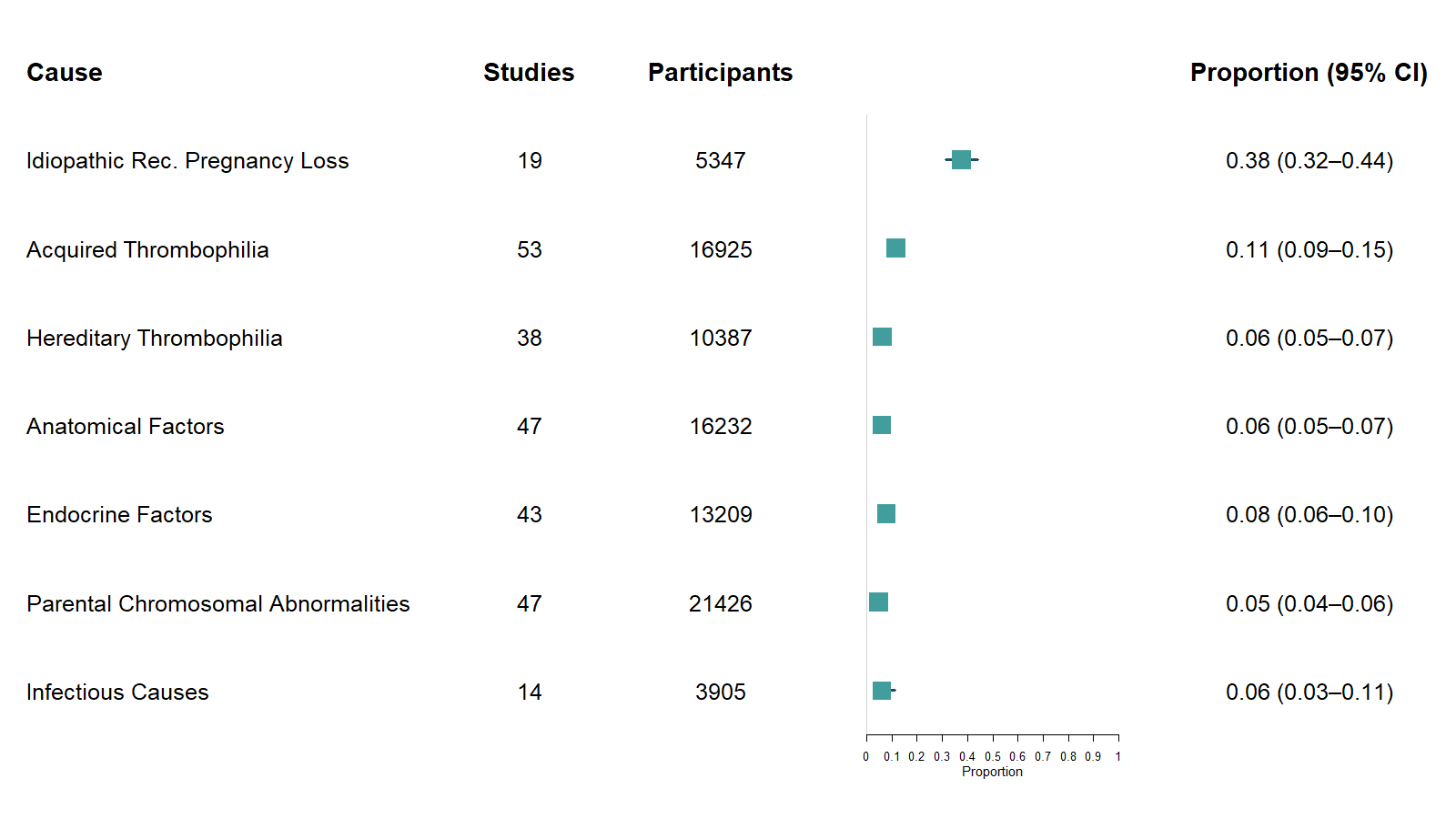

Supplement: SUPPLEMENTARY FIGURE S1 — Sensitivity analyses for the proportion of the major etiological categories of recurrent pregnancy loss, by excluding studies rated as high risk of bias. [file Data_sheet_1.zip › Supplementary Figures/SuppFig01.tiff]

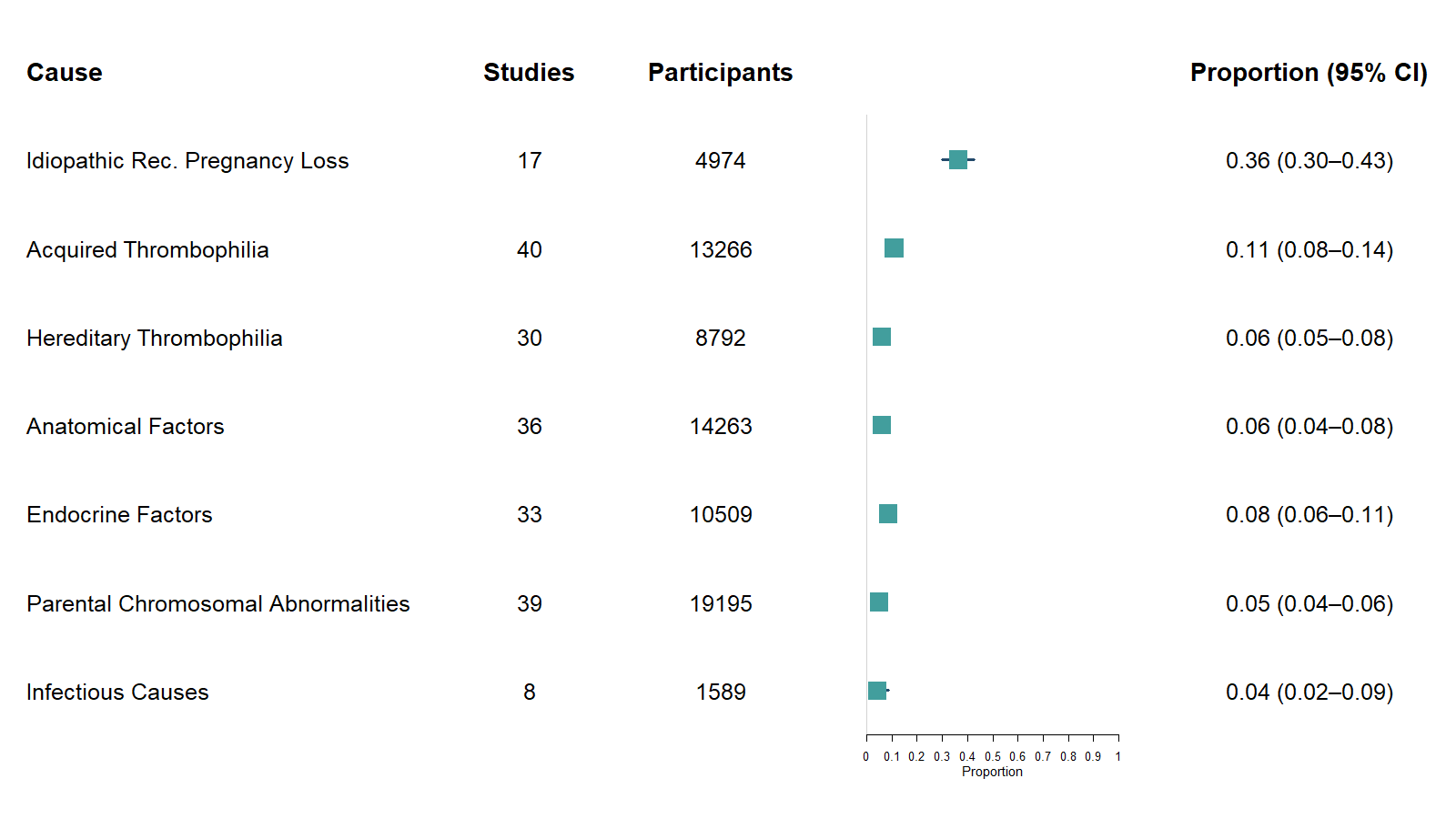

Supplement: SUPPLEMENTARY FIGURE S1 — Sensitivity analyses for the proportion of the major etiological categories of recurrent pregnancy loss, by excluding studies rated as high risk of bias. [file Data_sheet_1.zip › Supplementary Figures/SuppFig02.tiff]

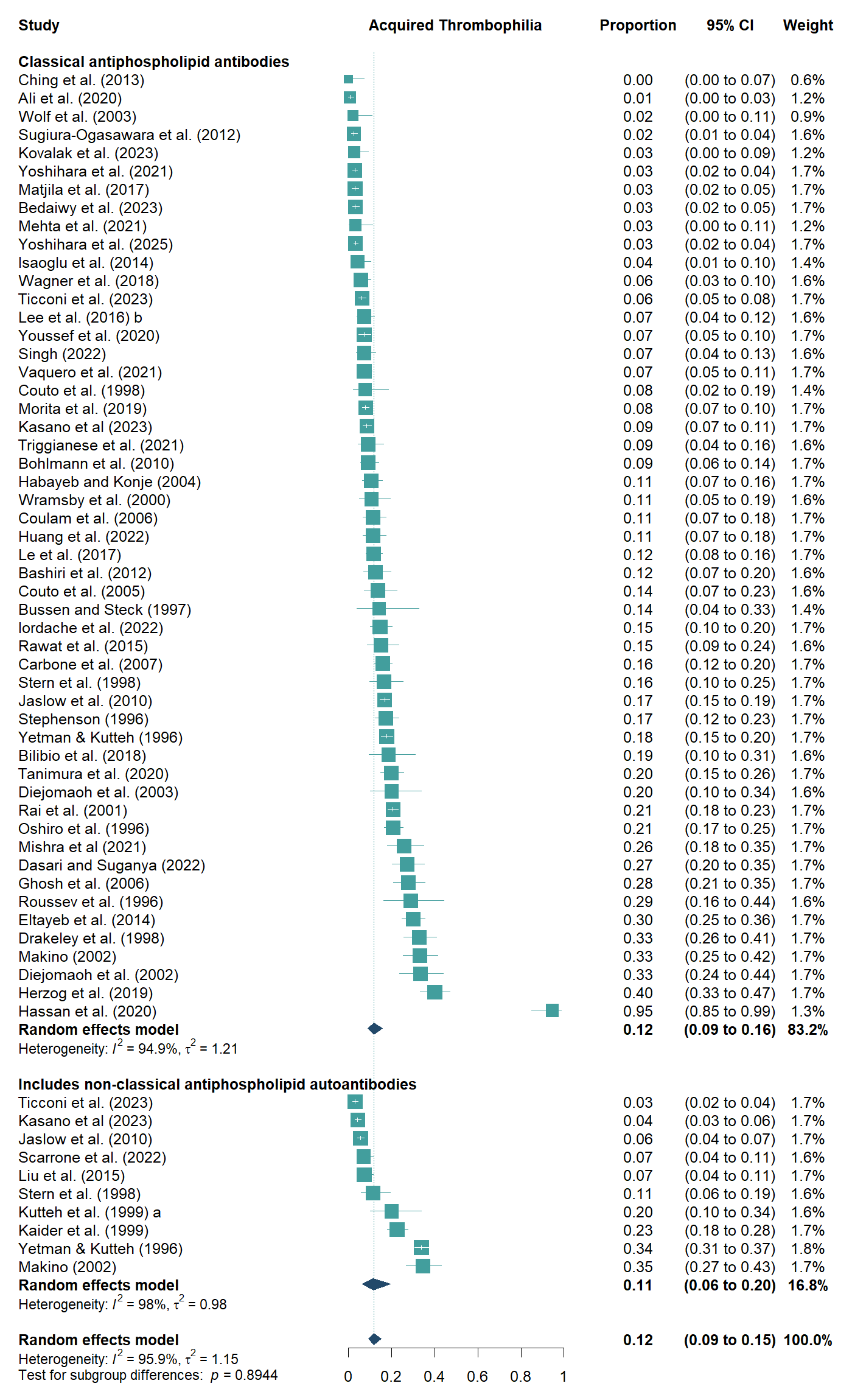

Supplement: SUPPLEMENTARY FIGURE S1 — Sensitivity analyses for the proportion of the major etiological categories of recurrent pregnancy loss, by excluding studies rated as high risk of bias. [file Data_sheet_1.zip › Supplementary Figures/SuppFig03.tiff]

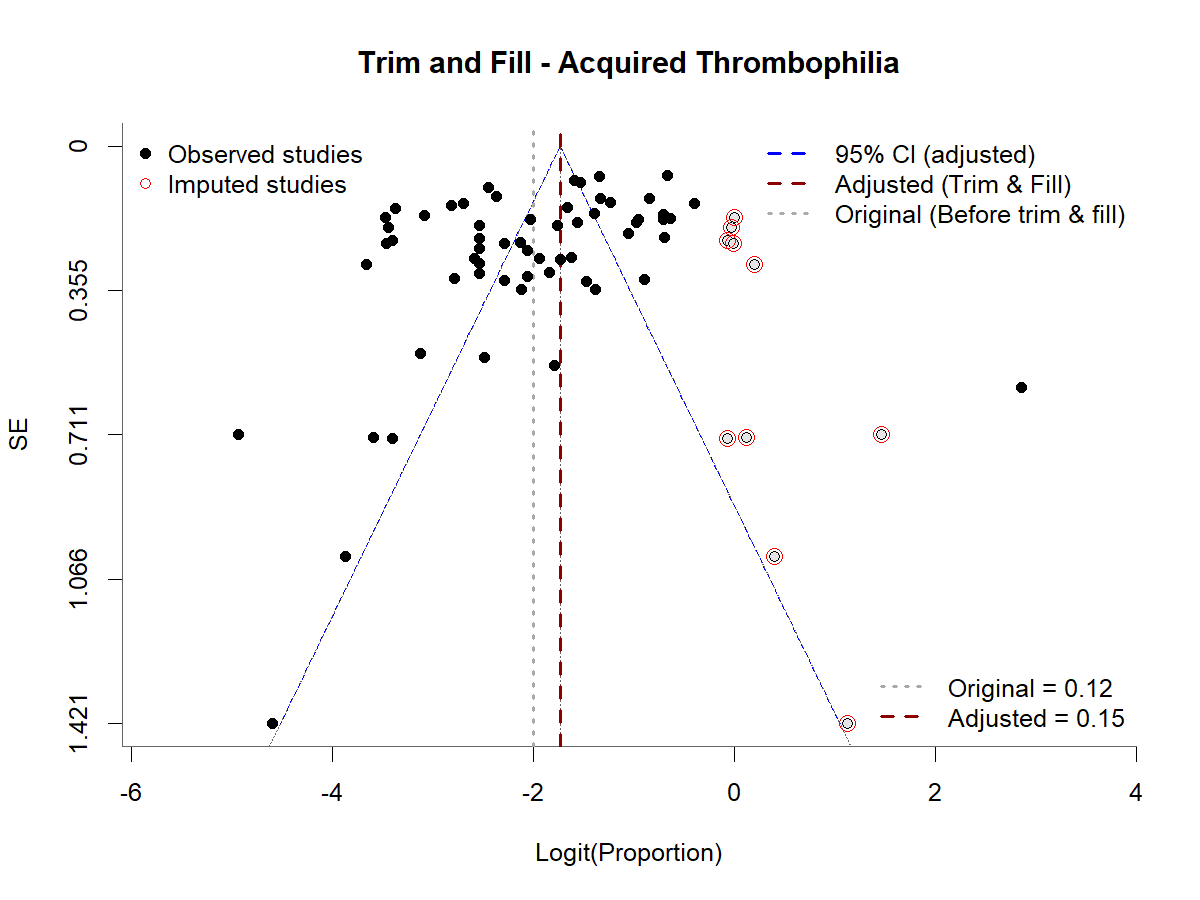

Supplement: SUPPLEMENTARY FIGURE S1 — Sensitivity analyses for the proportion of the major etiological categories of recurrent pregnancy loss, by excluding studies rated as high risk of bias. [file Data_sheet_1.zip › Supplementary Figures/SuppFig04.tiff]

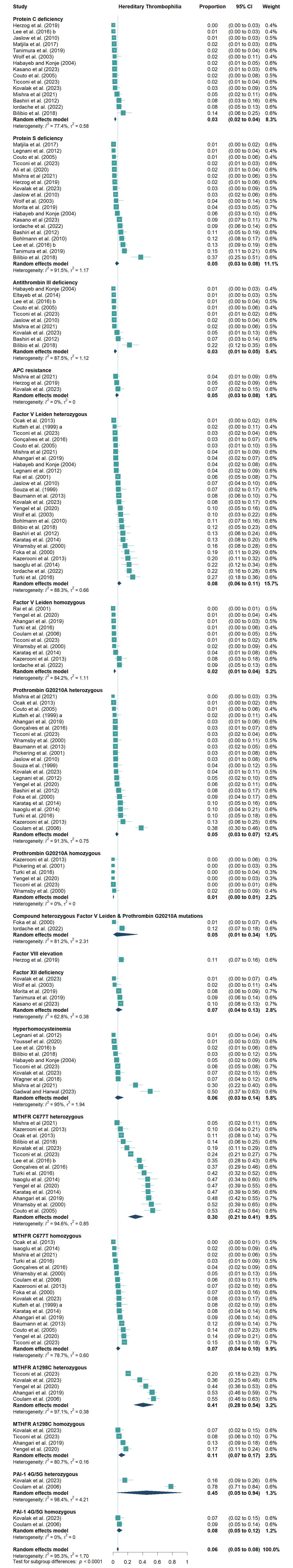

Supplement: SUPPLEMENTARY FIGURE S1 — Sensitivity analyses for the proportion of the major etiological categories of recurrent pregnancy loss, by excluding studies rated as high risk of bias. [file Data_sheet_1.zip › Supplementary Figures/SuppFig05.tiff]

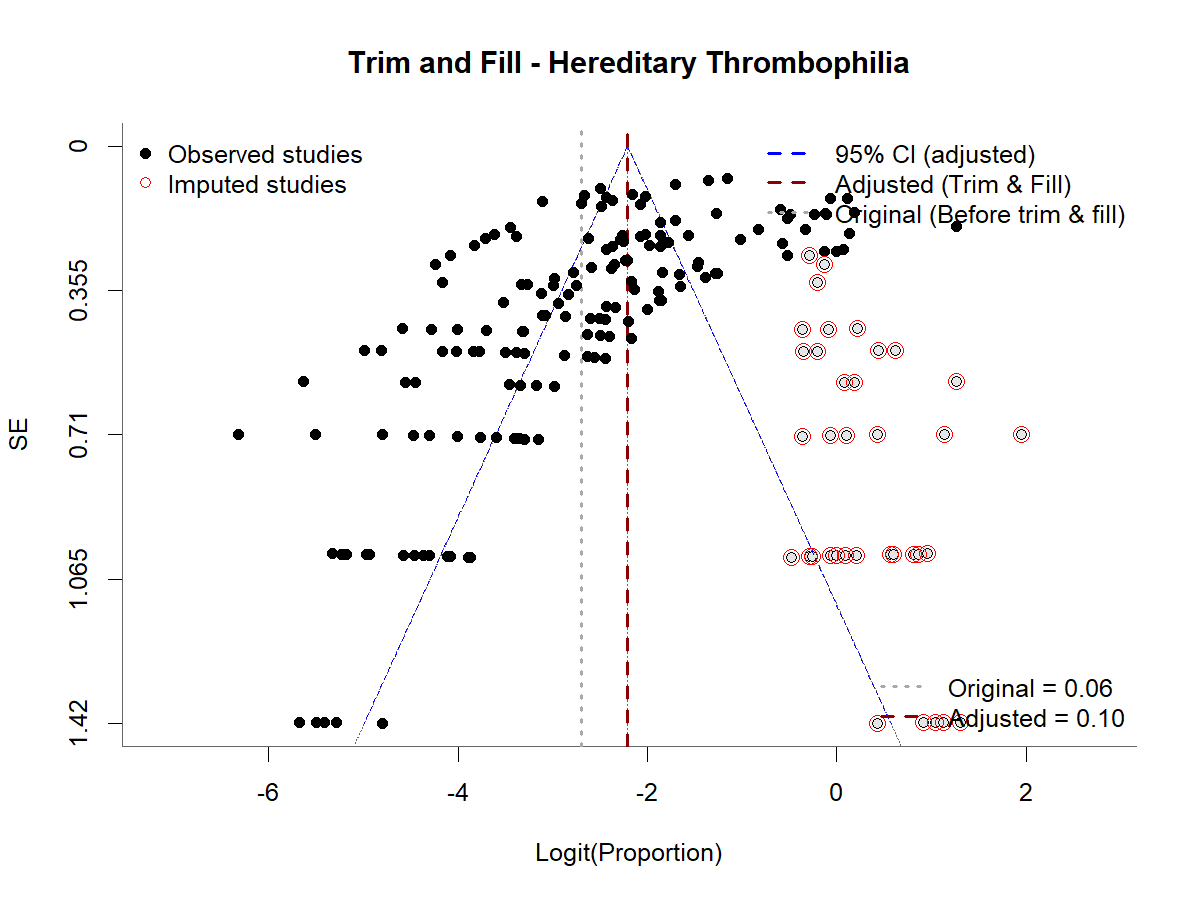

Supplement: SUPPLEMENTARY FIGURE S1 — Sensitivity analyses for the proportion of the major etiological categories of recurrent pregnancy loss, by excluding studies rated as high risk of bias. [file Data_sheet_1.zip › Supplementary Figures/SuppFig06.tiff]

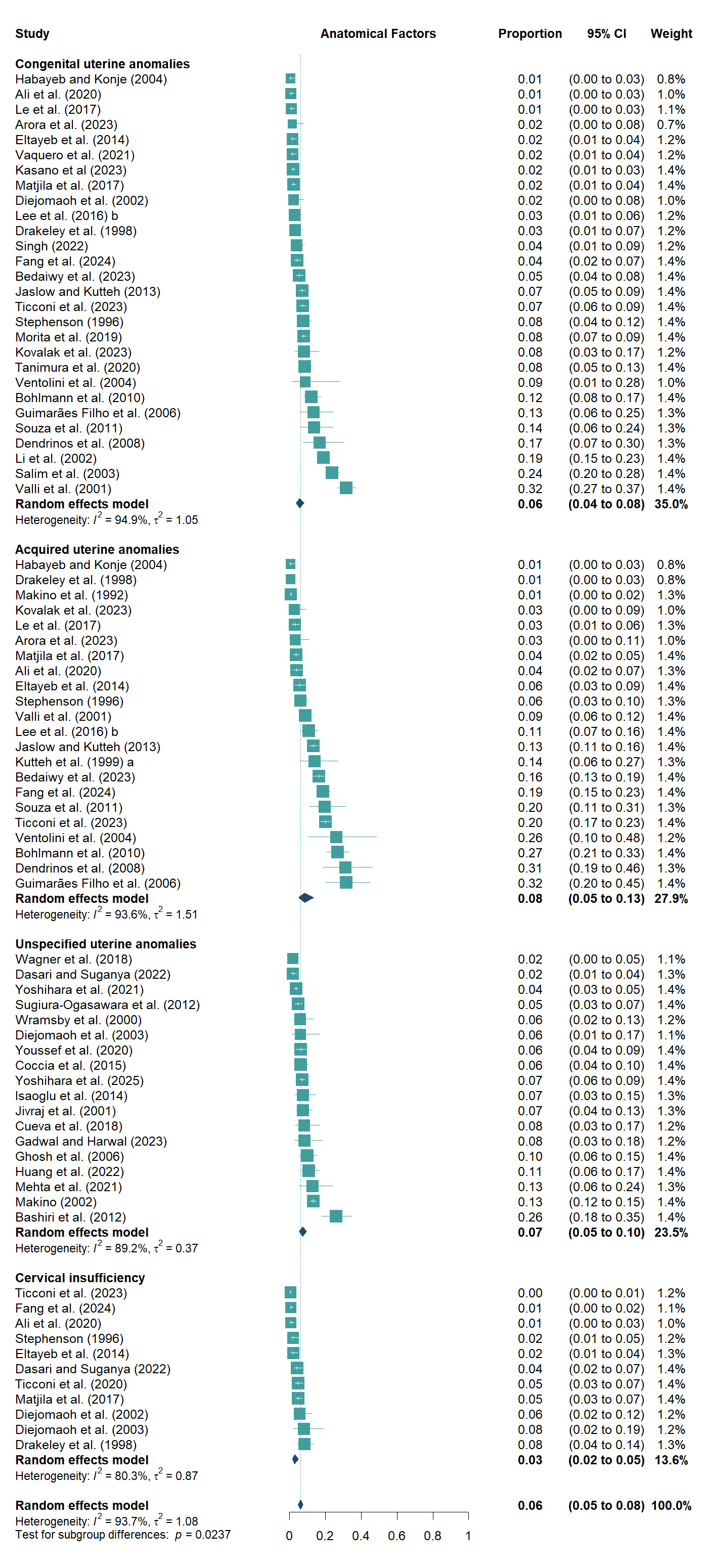

Supplement: SUPPLEMENTARY FIGURE S1 — Sensitivity analyses for the proportion of the major etiological categories of recurrent pregnancy loss, by excluding studies rated as high risk of bias. [file Data_sheet_1.zip › Supplementary Figures/SuppFig07.tiff]

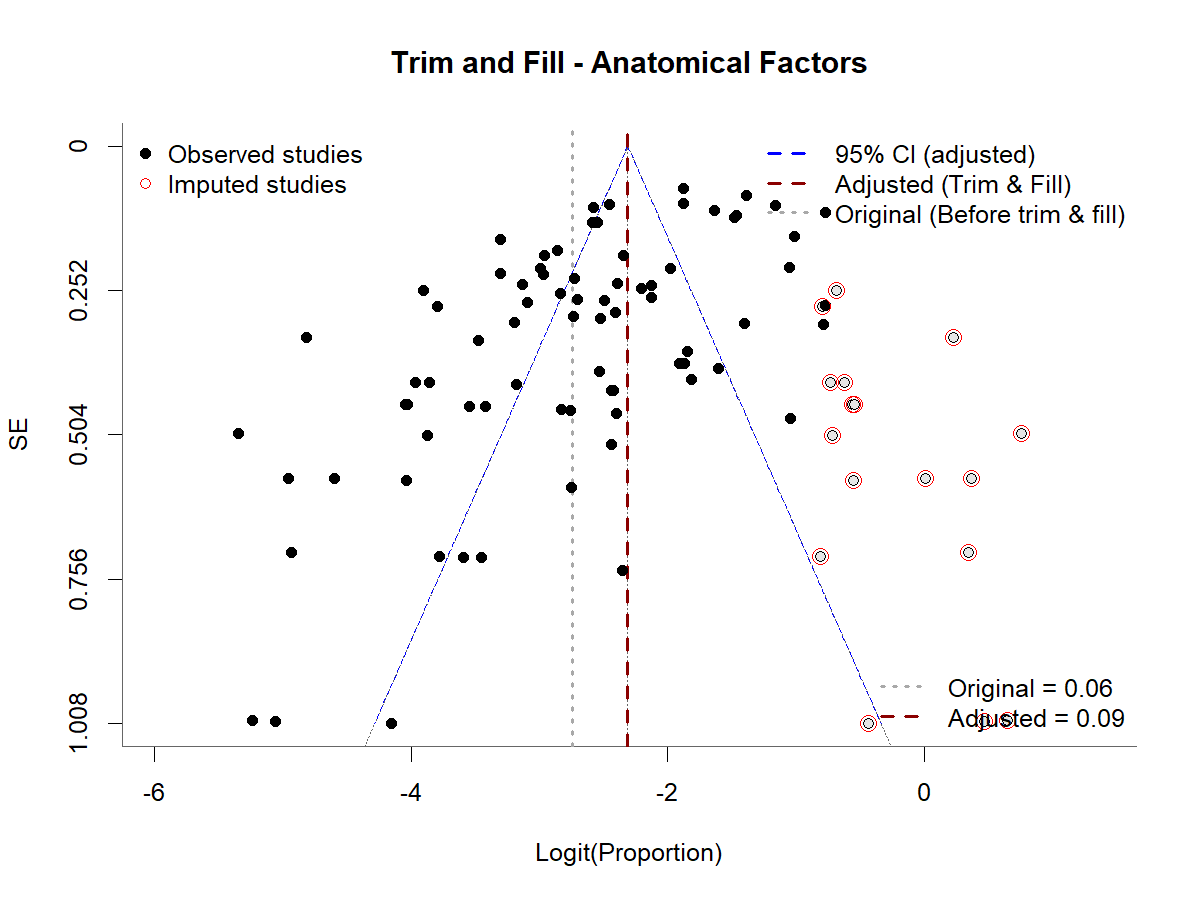

Supplement: SUPPLEMENTARY FIGURE S1 — Sensitivity analyses for the proportion of the major etiological categories of recurrent pregnancy loss, by excluding studies rated as high risk of bias. [file Data_sheet_1.zip › Supplementary Figures/SuppFig08.tiff]

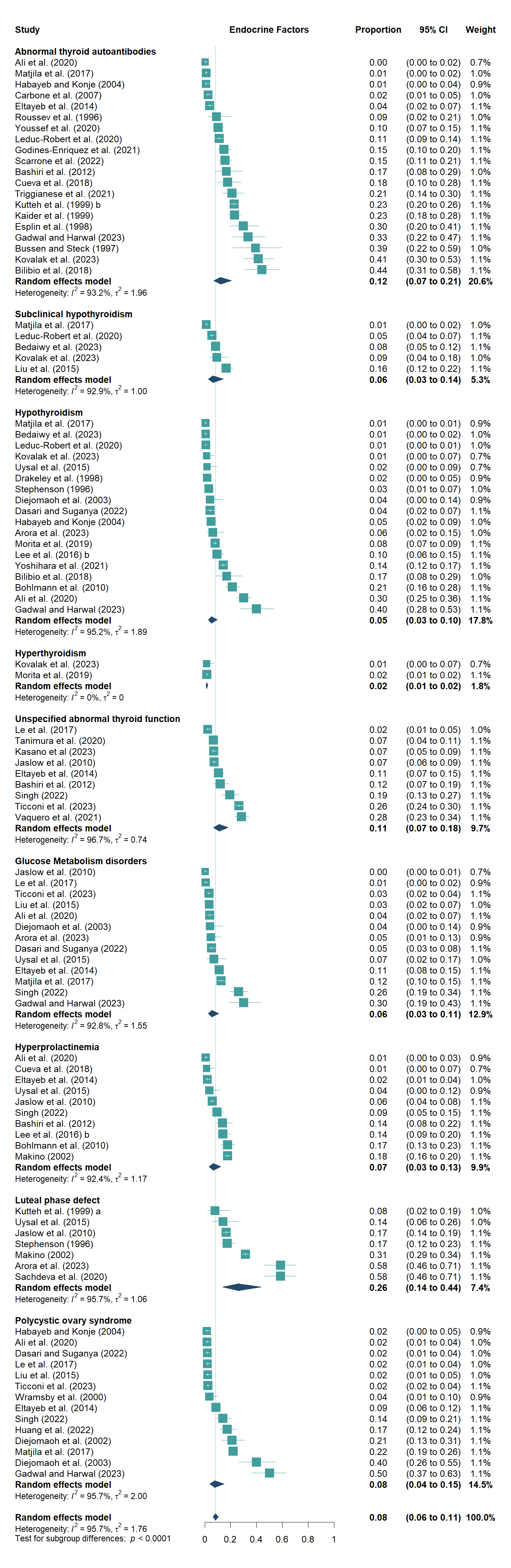

Supplement: SUPPLEMENTARY FIGURE S1 — Sensitivity analyses for the proportion of the major etiological categories of recurrent pregnancy loss, by excluding studies rated as high risk of bias. [file Data_sheet_1.zip › Supplementary Figures/SuppFig09.tiff]

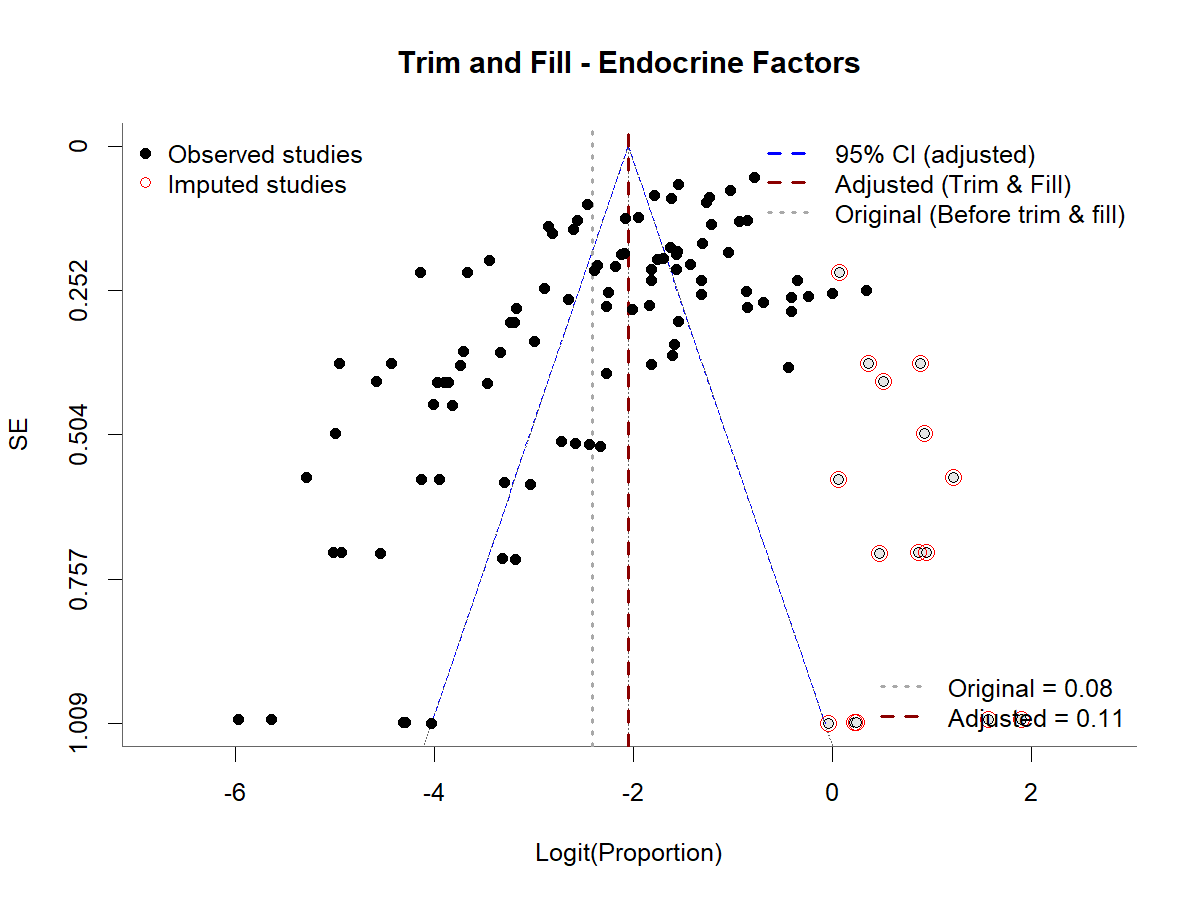

Supplement: SUPPLEMENTARY FIGURE S1 — Sensitivity analyses for the proportion of the major etiological categories of recurrent pregnancy loss, by excluding studies rated as high risk of bias. [file Data_sheet_1.zip › Supplementary Figures/SuppFig10.tiff]

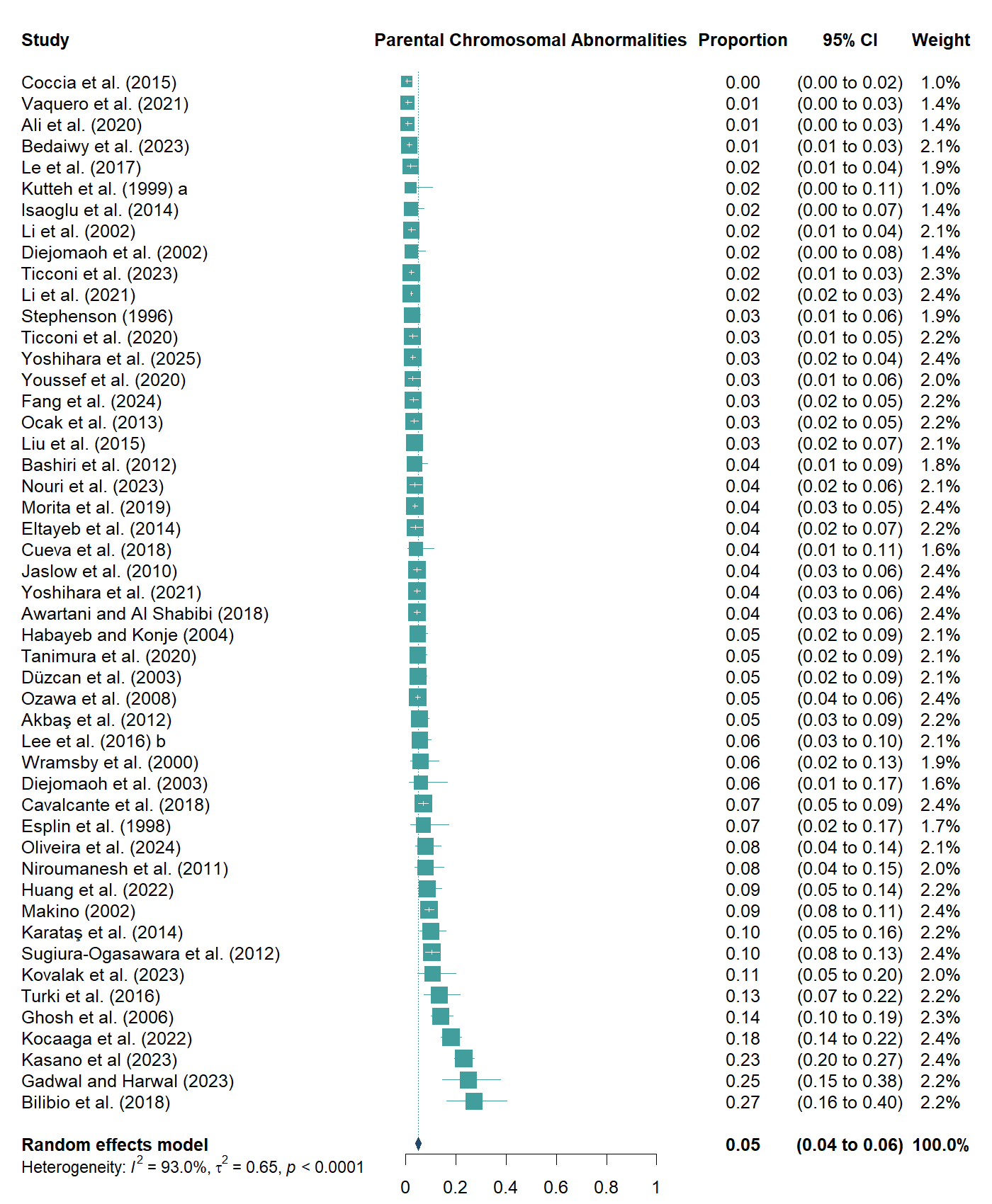

Supplement: SUPPLEMENTARY FIGURE S1 — Sensitivity analyses for the proportion of the major etiological categories of recurrent pregnancy loss, by excluding studies rated as high risk of bias. [file Data_sheet_1.zip › Supplementary Figures/SuppFig11.tiff]

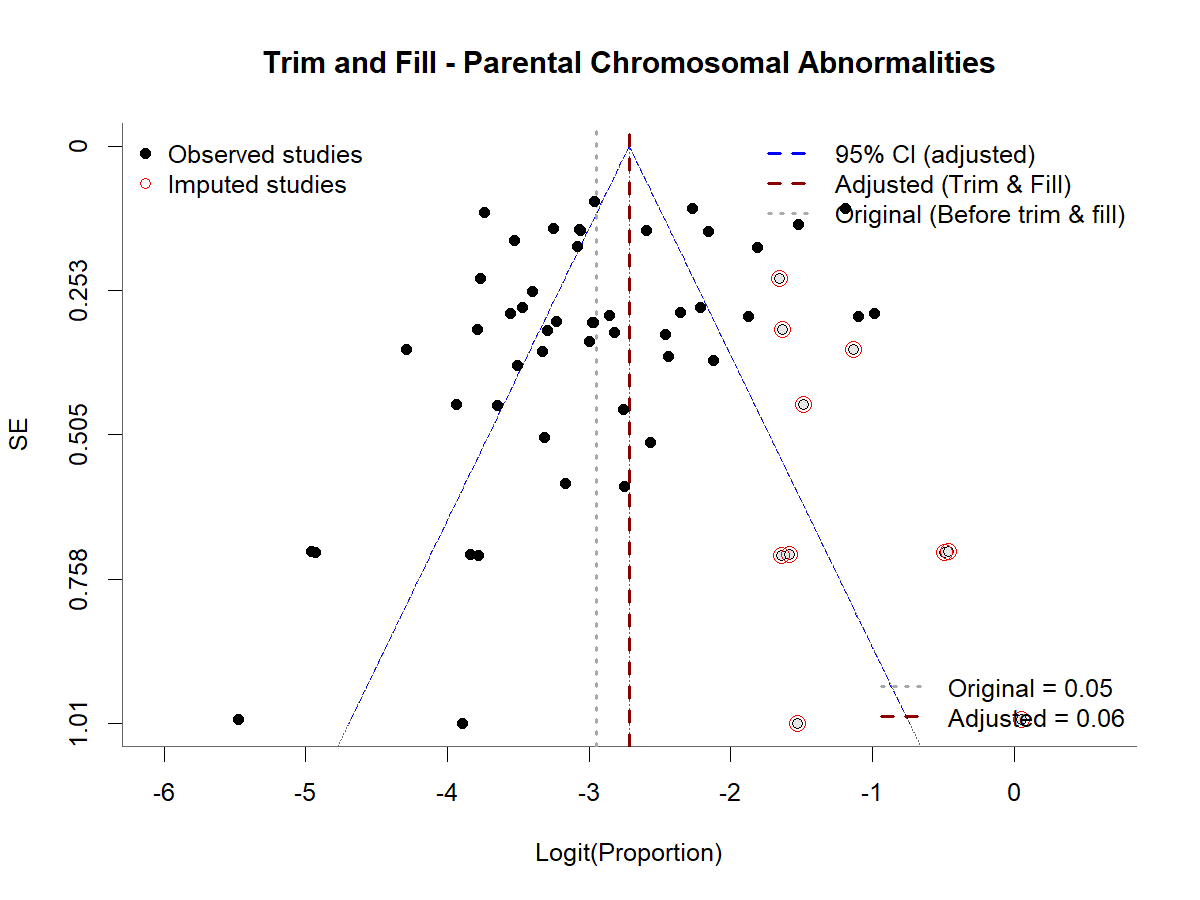

Supplement: SUPPLEMENTARY FIGURE S1 — Sensitivity analyses for the proportion of the major etiological categories of recurrent pregnancy loss, by excluding studies rated as high risk of bias. [file Data_sheet_1.zip › Supplementary Figures/SuppFig12.tiff]

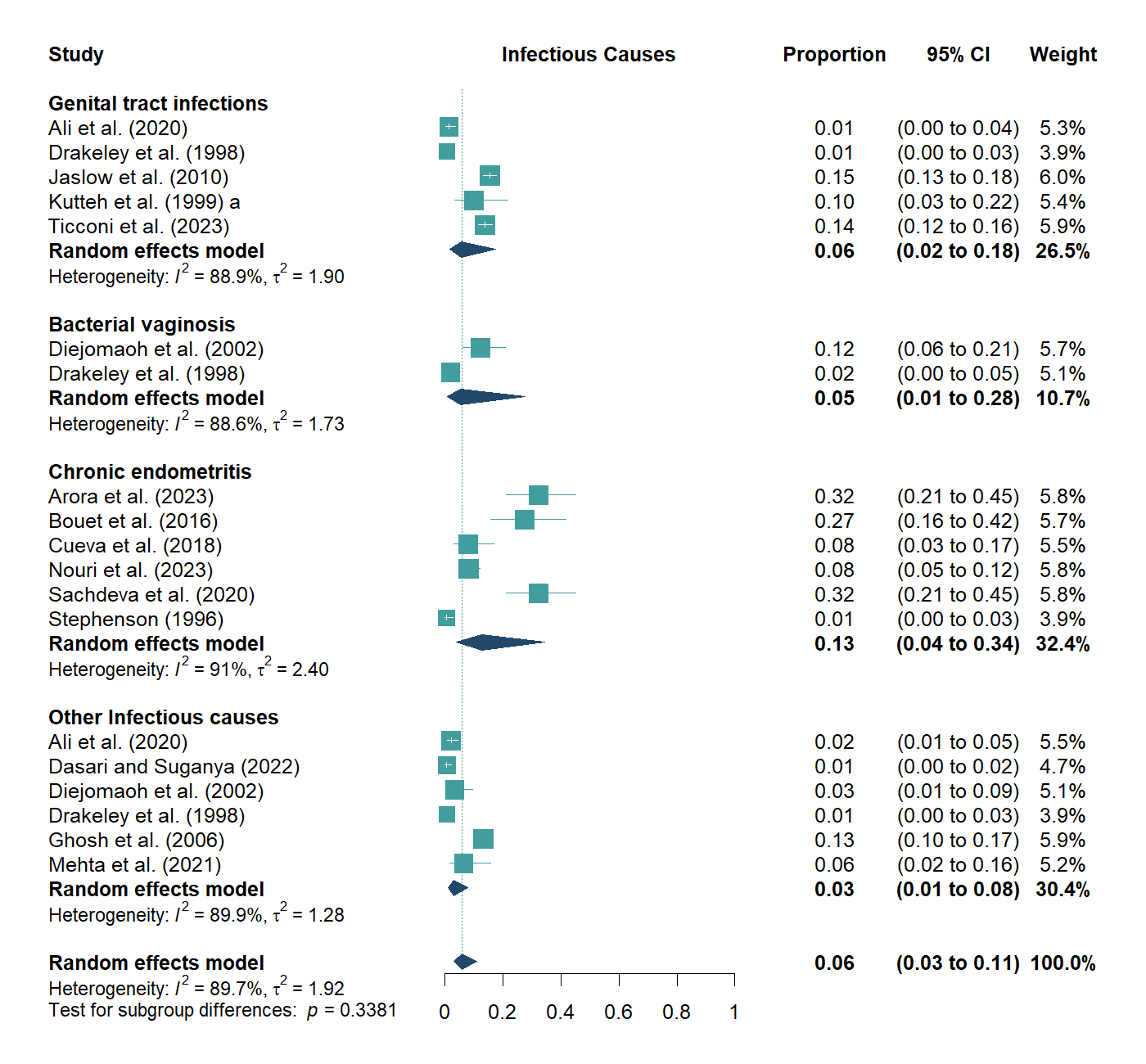

Supplement: SUPPLEMENTARY FIGURE S1 — Sensitivity analyses for the proportion of the major etiological categories of recurrent pregnancy loss, by excluding studies rated as high risk of bias. [file Data_sheet_1.zip › Supplementary Figures/SuppFig13.tiff]

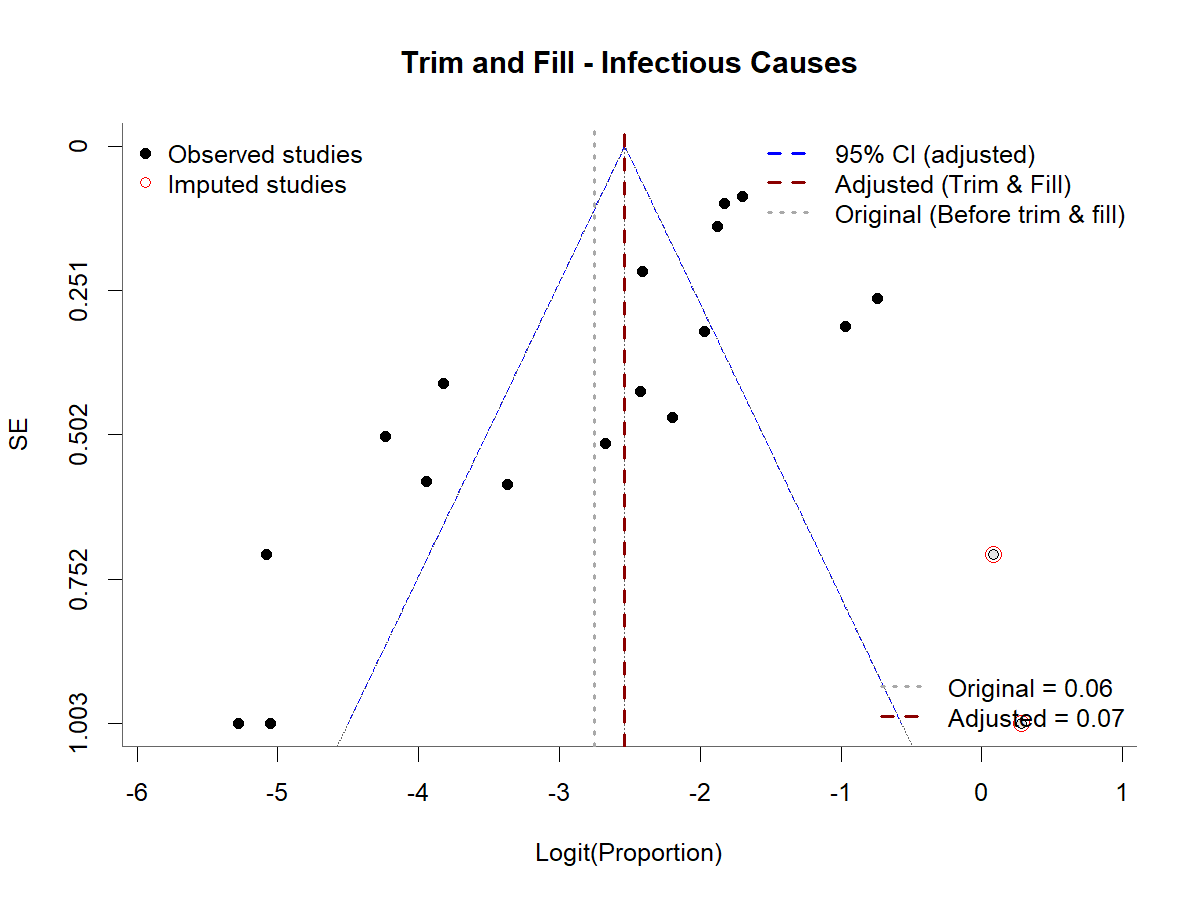

Supplement: SUPPLEMENTARY FIGURE S1 — Sensitivity analyses for the proportion of the major etiological categories of recurrent pregnancy loss, by excluding studies rated as high risk of bias. [file Data_sheet_1.zip › Supplementary Figures/SuppFig14.tiff]

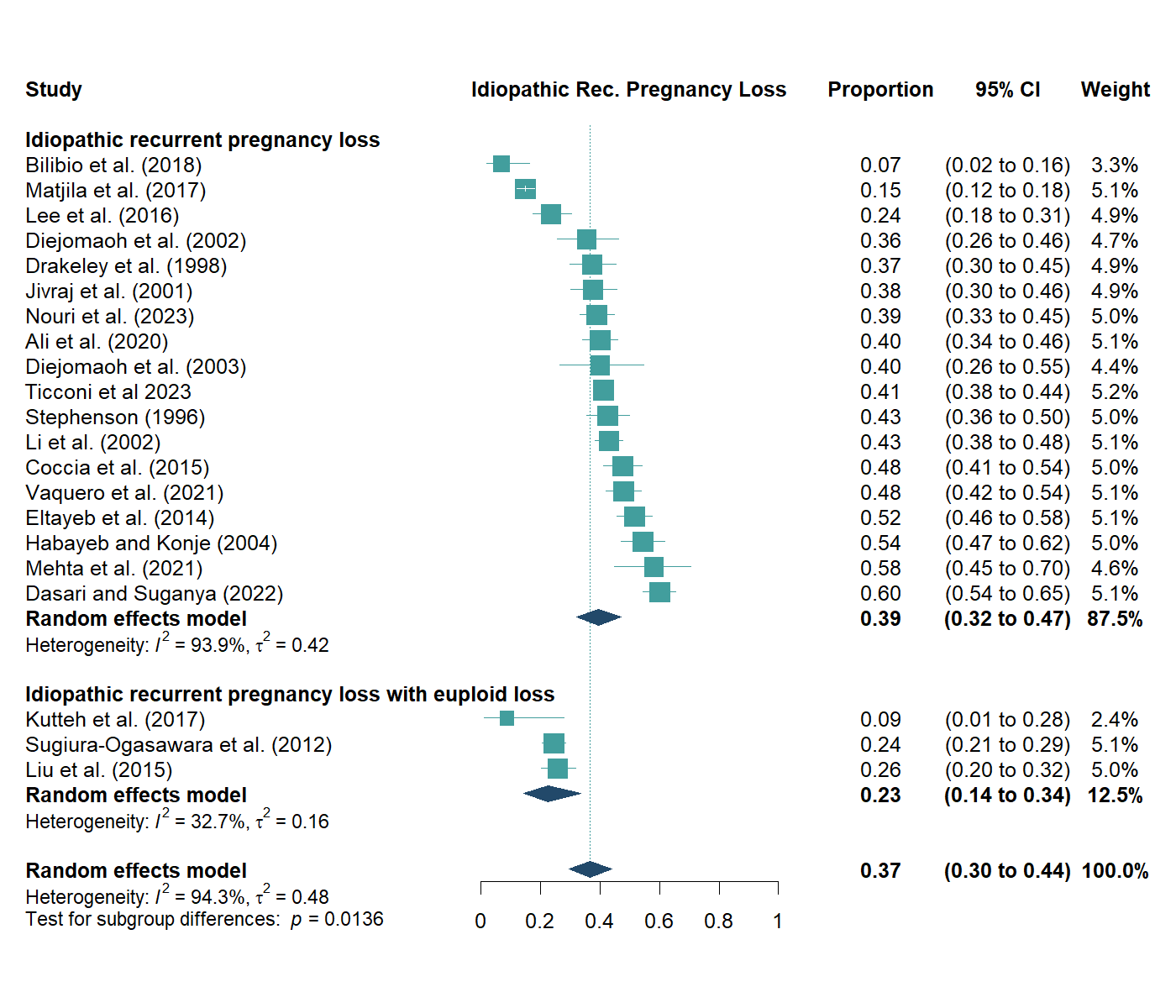

Supplement: SUPPLEMENTARY FIGURE S1 — Sensitivity analyses for the proportion of the major etiological categories of recurrent pregnancy loss, by excluding studies rated as high risk of bias. [file Data_sheet_1.zip › Supplementary Figures/SuppFig15.tiff]

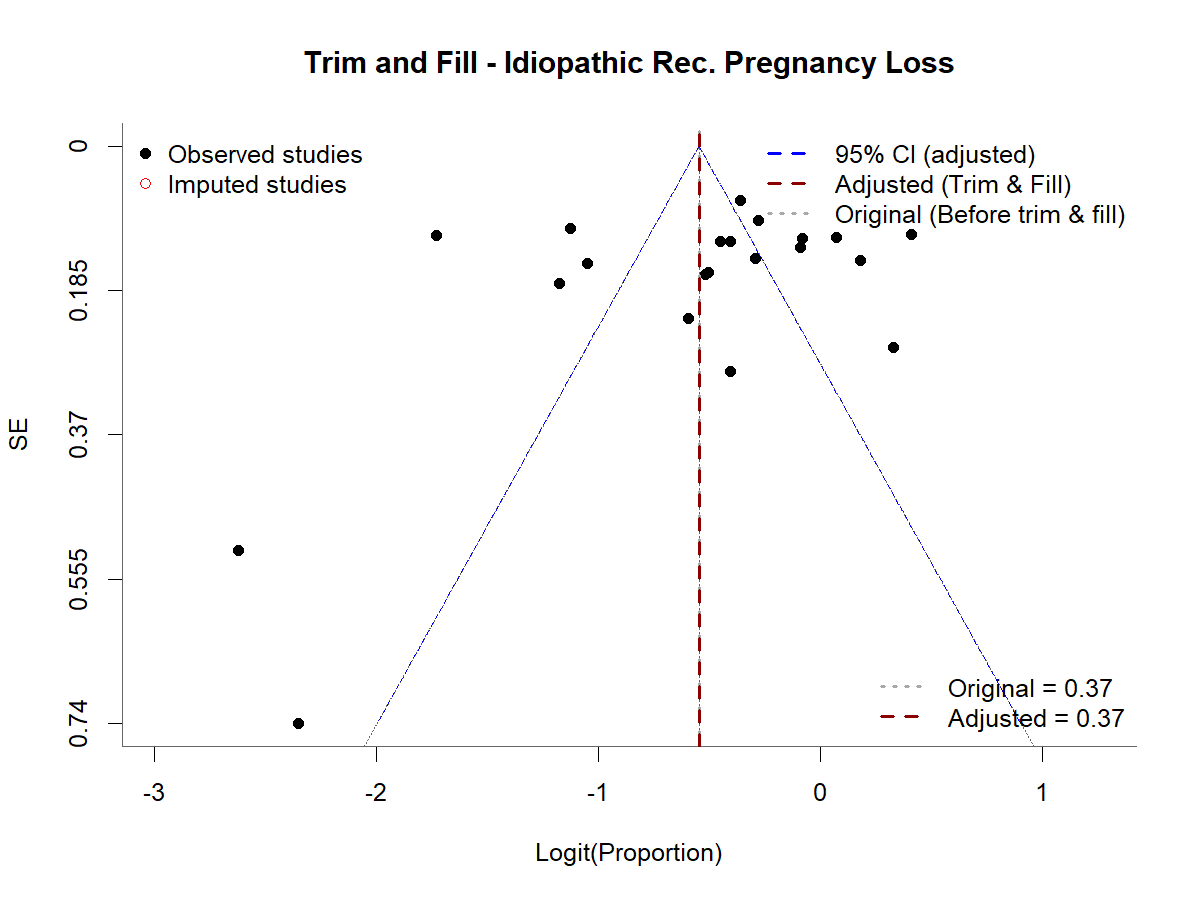

Supplement: SUPPLEMENTARY FIGURE S1 — Sensitivity analyses for the proportion of the major etiological categories of recurrent pregnancy loss, by excluding studies rated as high risk of bias. [file Data_sheet_1.zip › Supplementary Figures/SuppFig16.tiff]

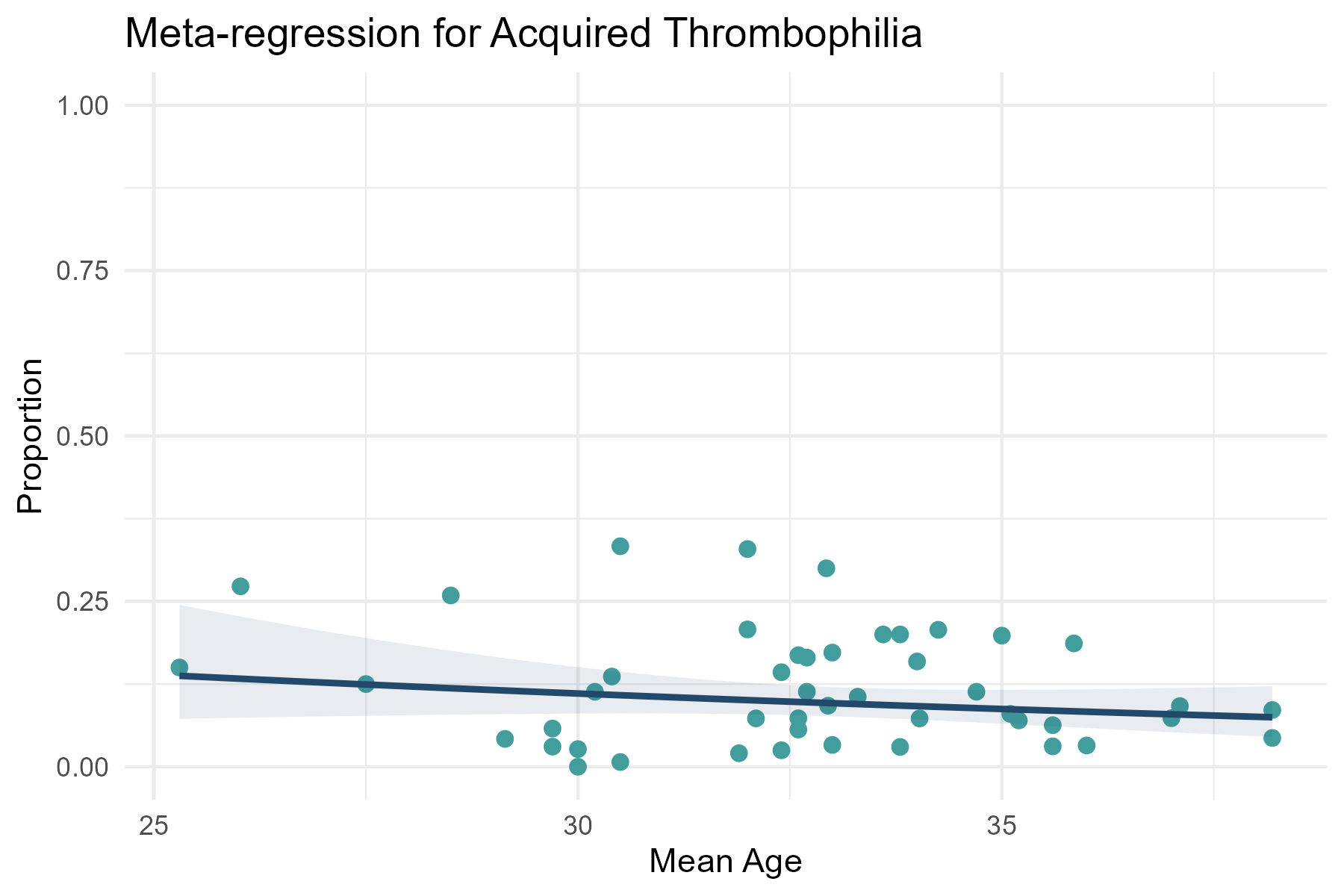

Supplement: SUPPLEMENTARY FIGURE S1 — Sensitivity analyses for the proportion of the major etiological categories of recurrent pregnancy loss, by excluding studies rated as high risk of bias. [file Data_sheet_1.zip › Supplementary Figures/SuppFig17.tiff]

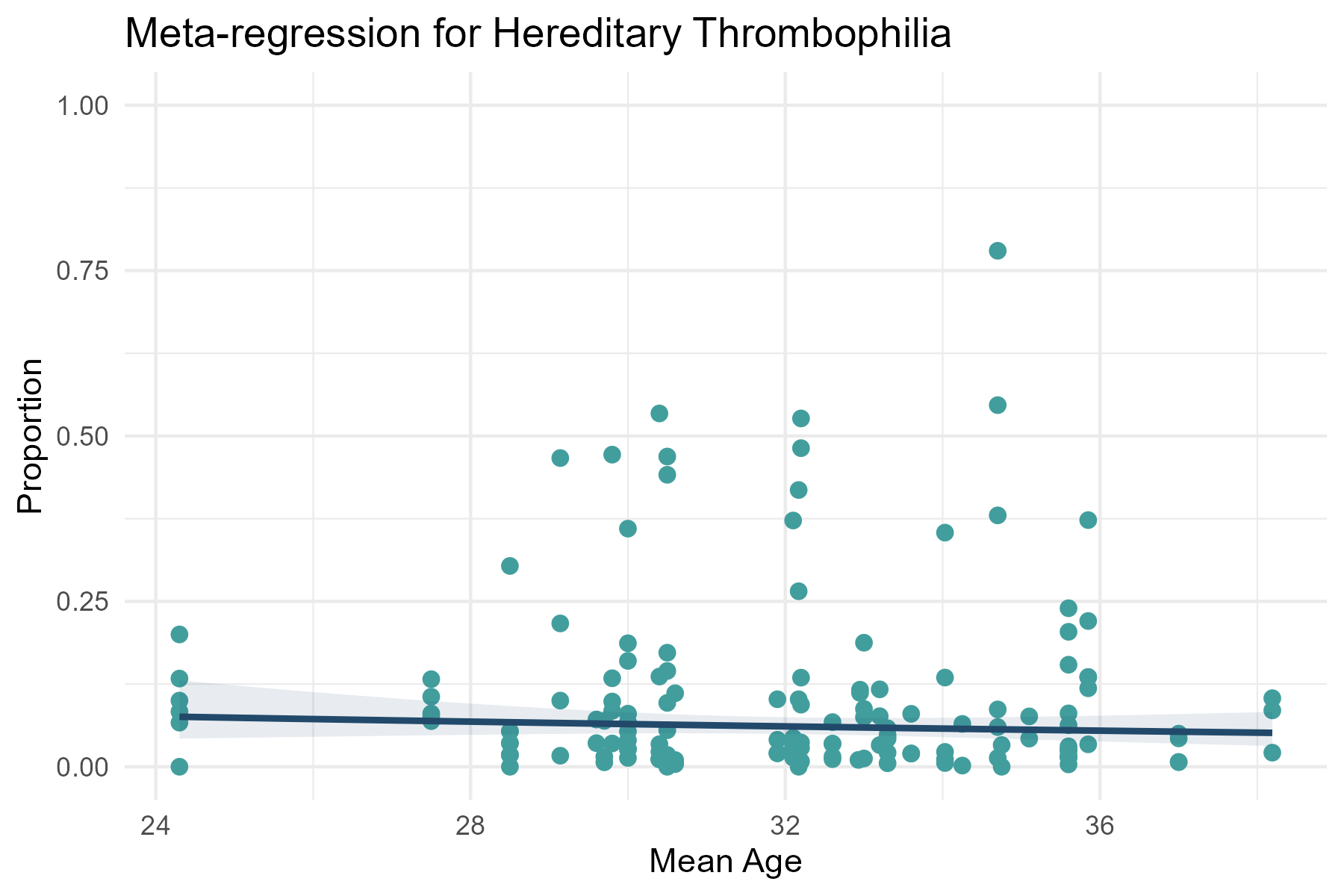

Supplement: SUPPLEMENTARY FIGURE S1 — Sensitivity analyses for the proportion of the major etiological categories of recurrent pregnancy loss, by excluding studies rated as high risk of bias. [file Data_sheet_1.zip › Supplementary Figures/SuppFig18.tiff]

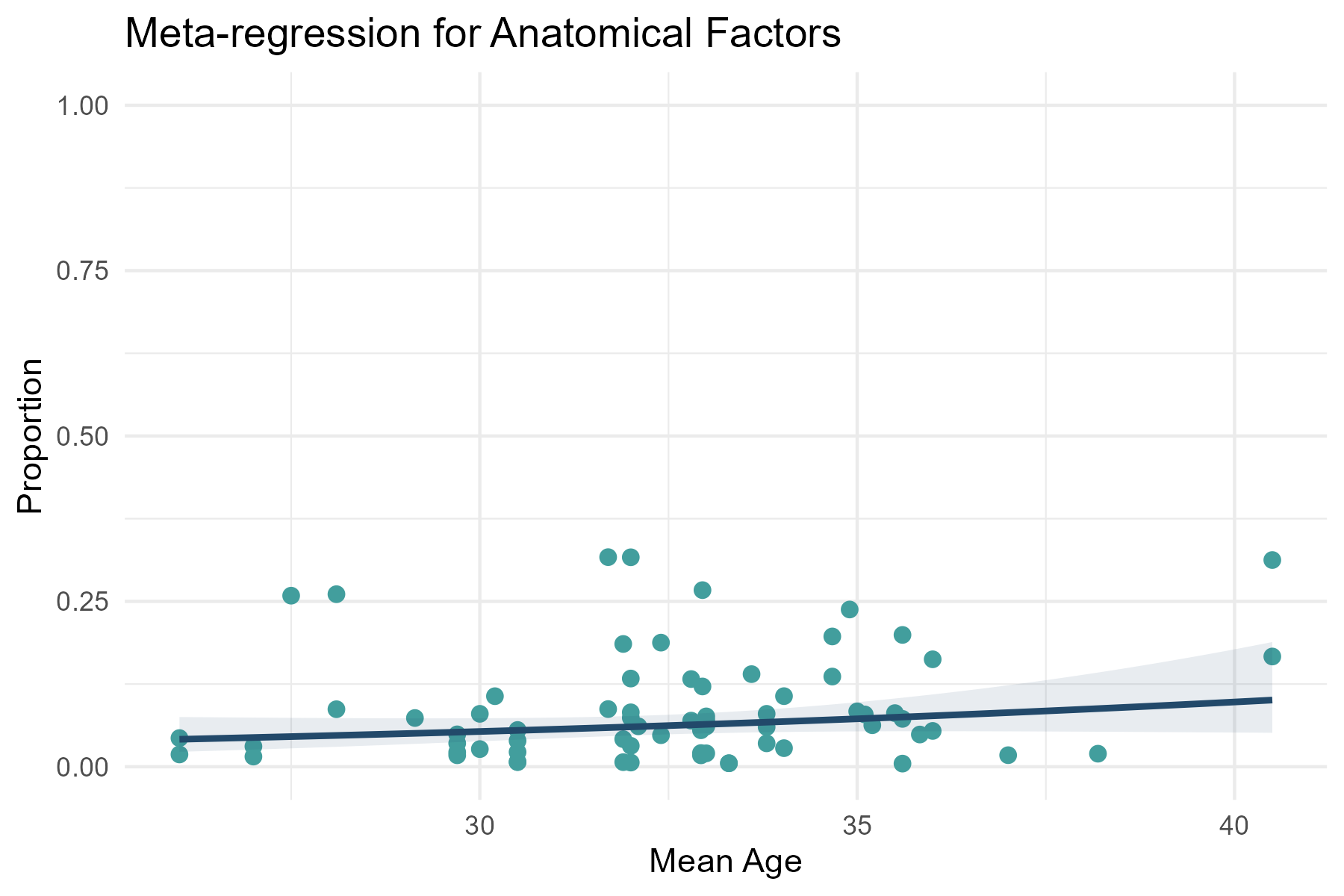

Supplement: SUPPLEMENTARY FIGURE S1 — Sensitivity analyses for the proportion of the major etiological categories of recurrent pregnancy loss, by excluding studies rated as high risk of bias. [file Data_sheet_1.zip › Supplementary Figures/SuppFig19.tiff]

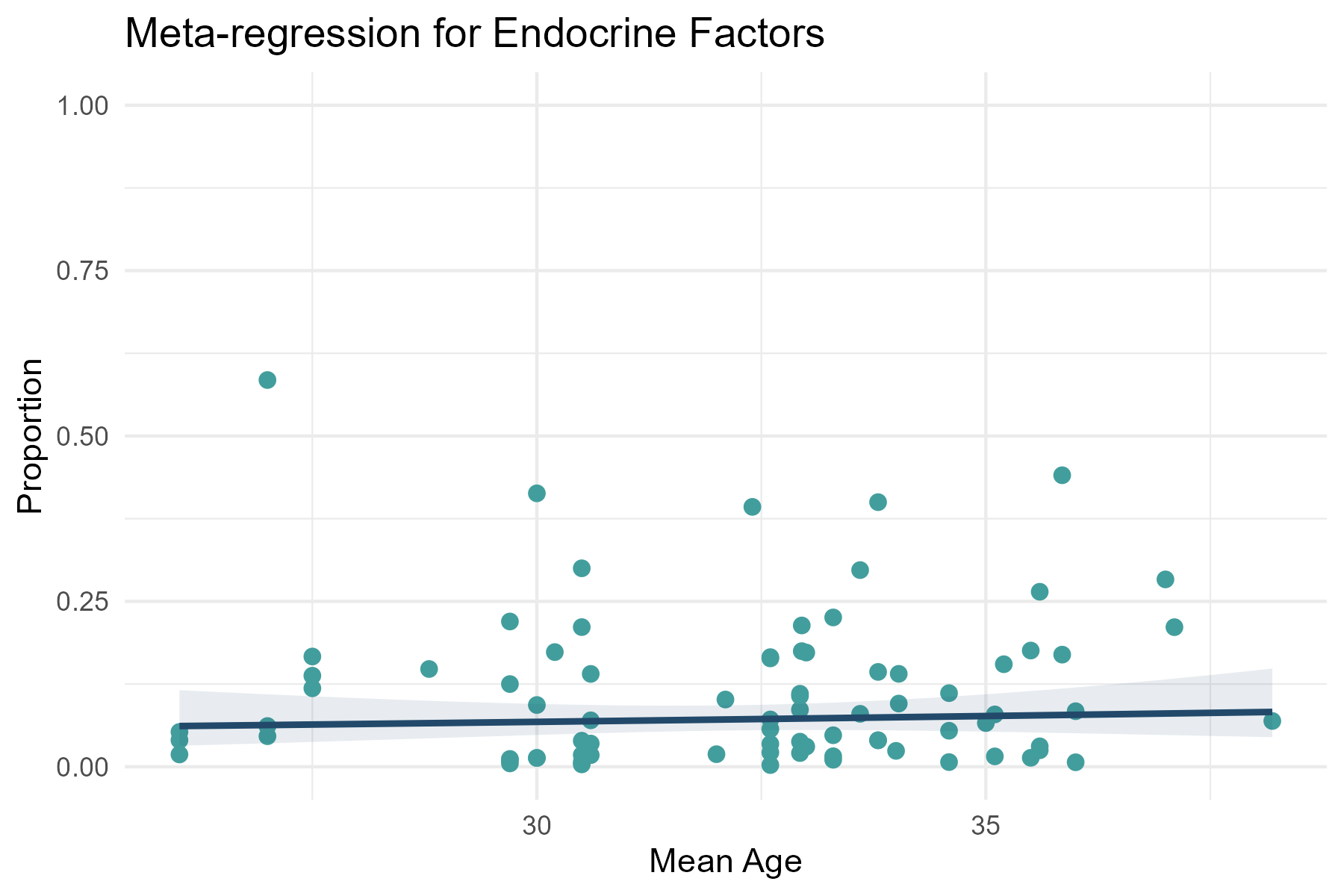

Supplement: SUPPLEMENTARY FIGURE S1 — Sensitivity analyses for the proportion of the major etiological categories of recurrent pregnancy loss, by excluding studies rated as high risk of bias. [file Data_sheet_1.zip › Supplementary Figures/SuppFig20.tiff]

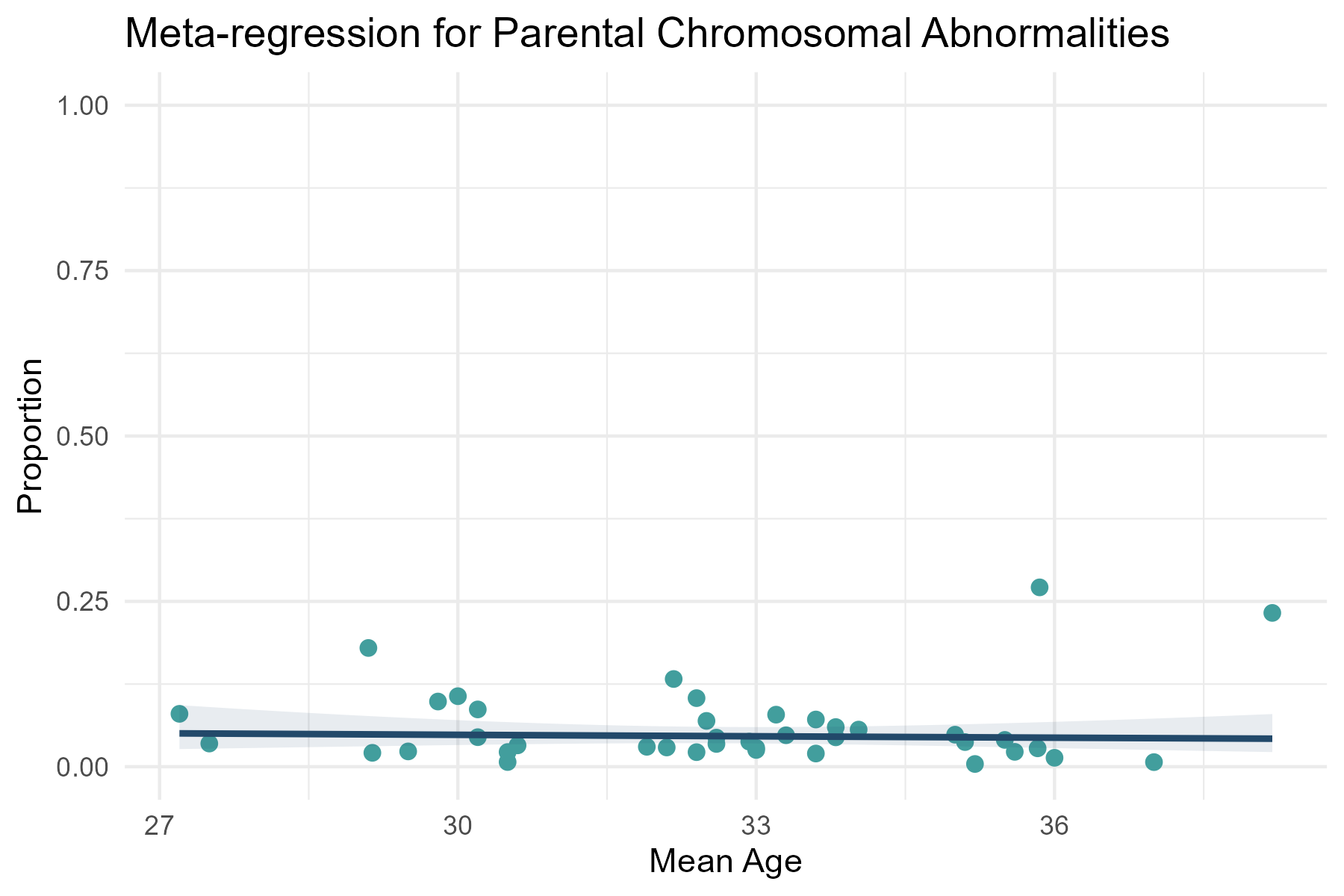

Supplement: SUPPLEMENTARY FIGURE S1 — Sensitivity analyses for the proportion of the major etiological categories of recurrent pregnancy loss, by excluding studies rated as high risk of bias. [file Data_sheet_1.zip › Supplementary Figures/SuppFig21.tiff]

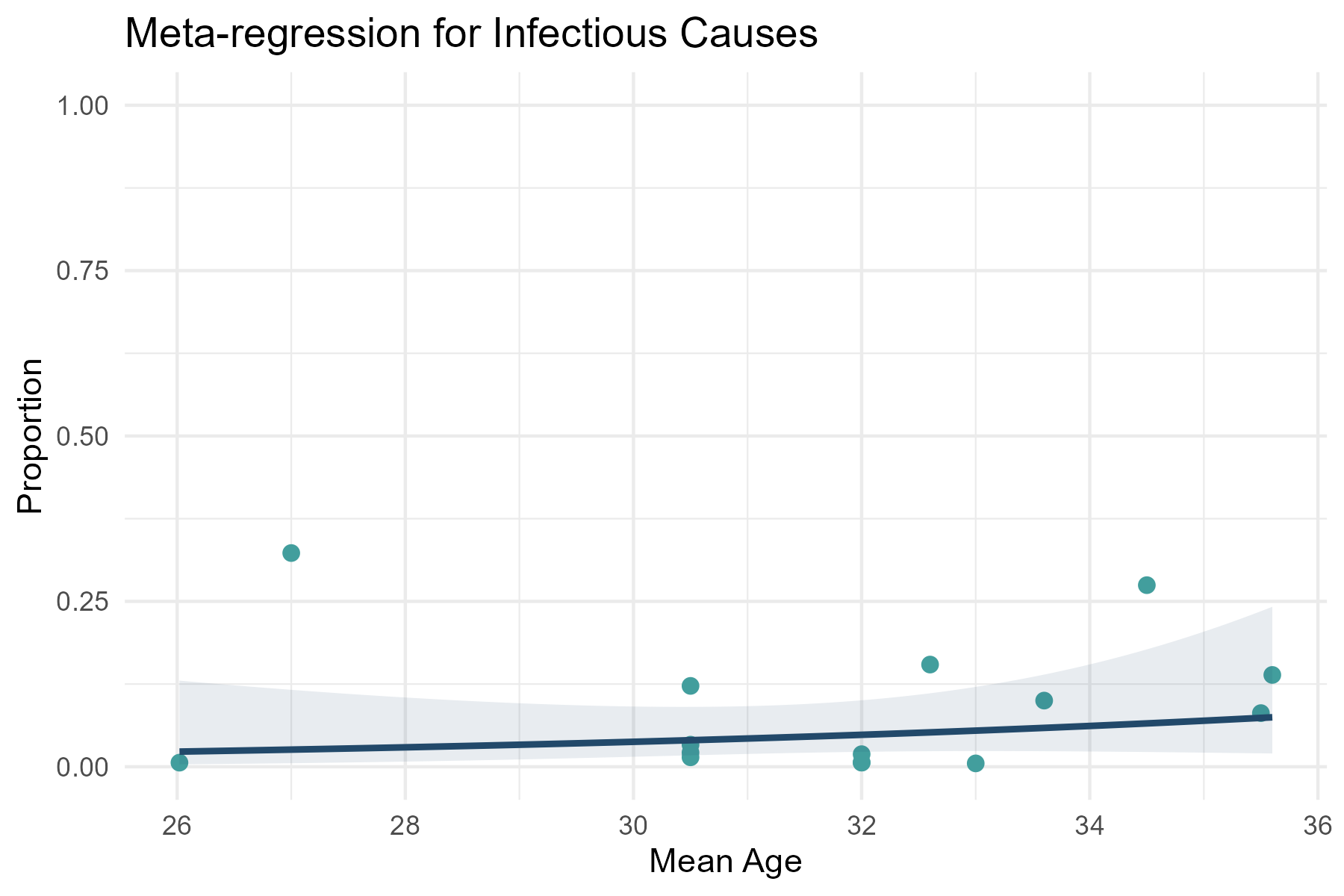

Supplement: SUPPLEMENTARY FIGURE S1 — Sensitivity analyses for the proportion of the major etiological categories of recurrent pregnancy loss, by excluding studies rated as high risk of bias. [file Data_sheet_1.zip › Supplementary Figures/SuppFig22.tiff]

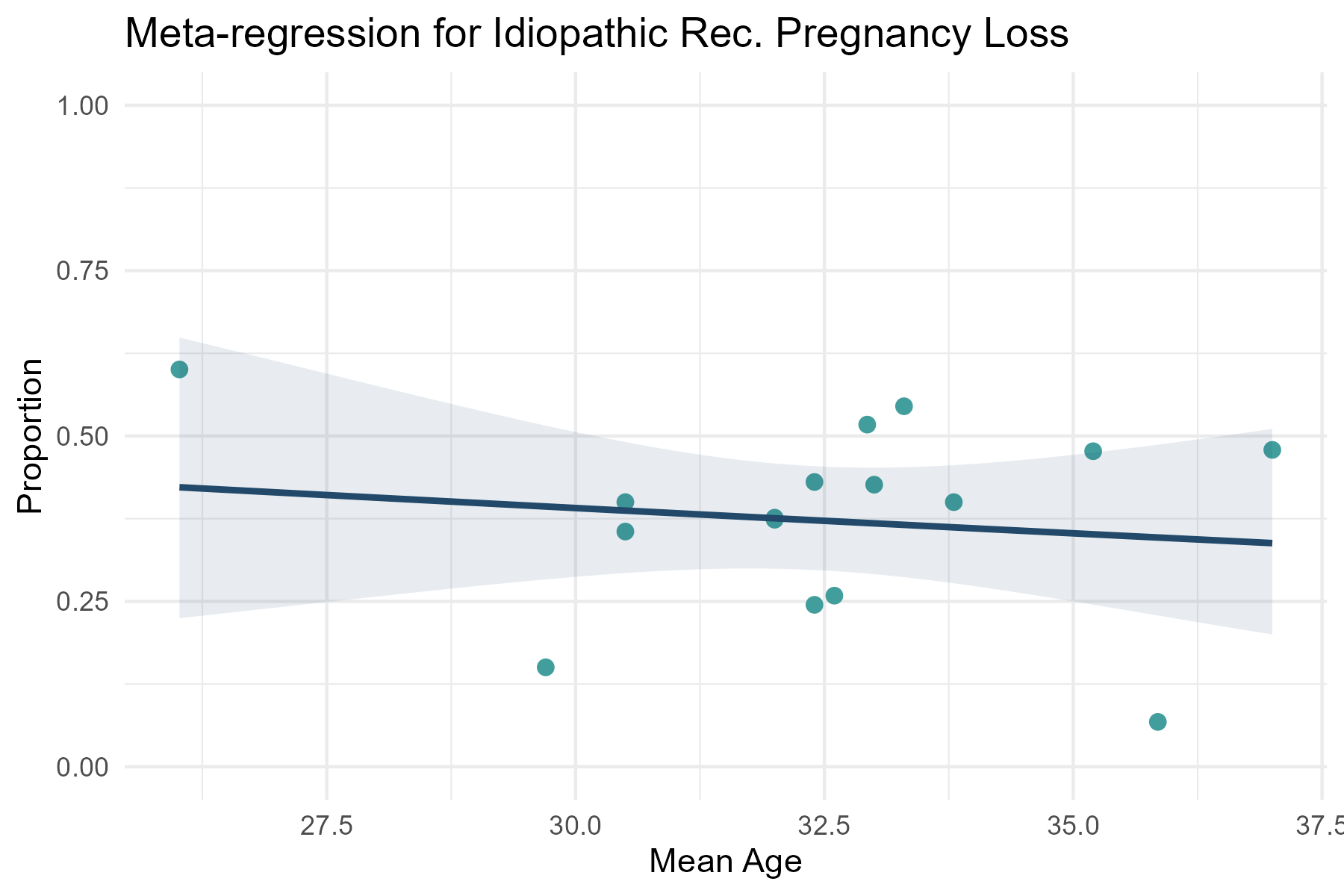

Supplement: SUPPLEMENTARY FIGURE S1 — Sensitivity analyses for the proportion of the major etiological categories of recurrent pregnancy loss, by excluding studies rated as high risk of bias. [file Data_sheet_1.zip › Supplementary Figures/SuppFig23.tiff]

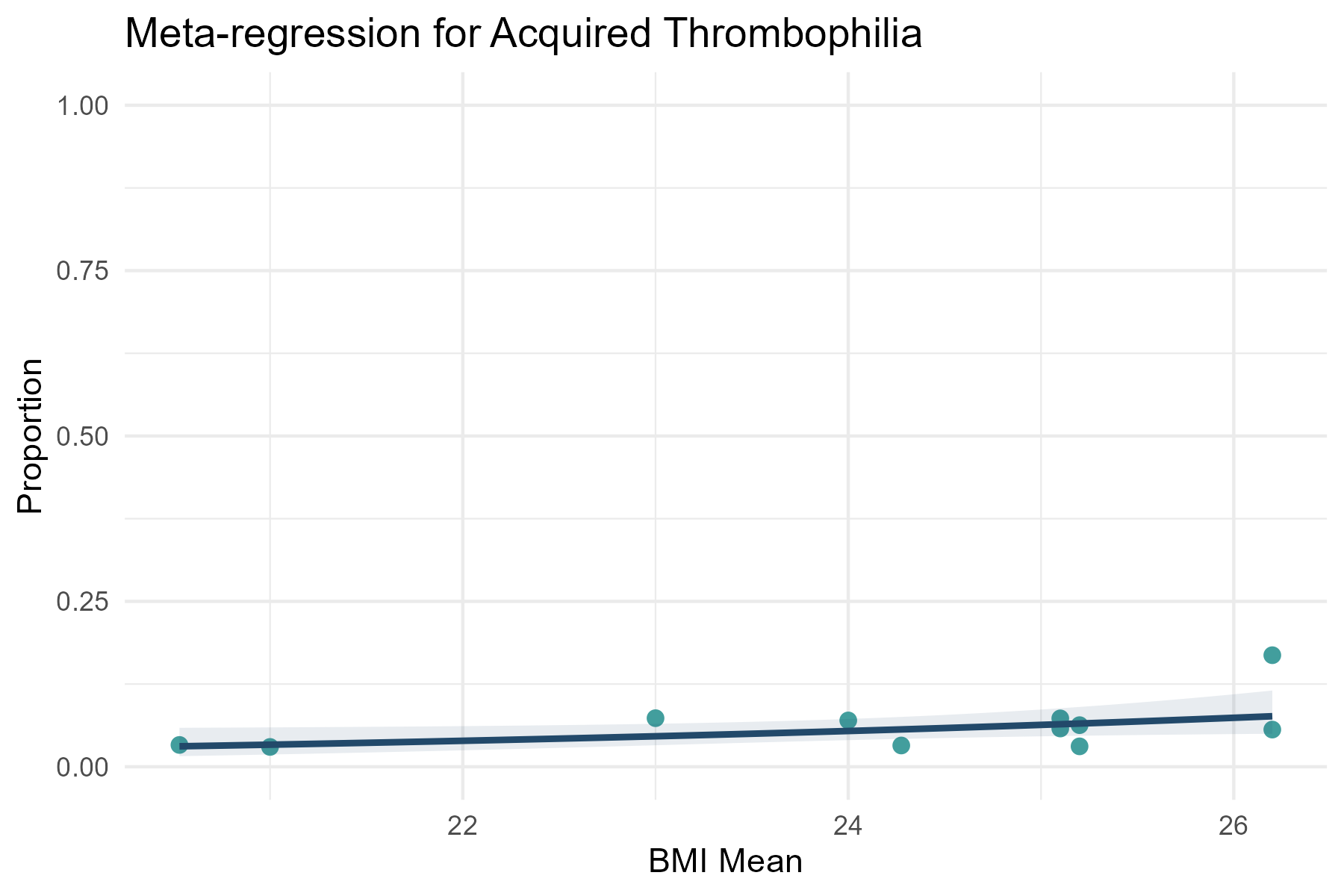

Supplement: SUPPLEMENTARY FIGURE S1 — Sensitivity analyses for the proportion of the major etiological categories of recurrent pregnancy loss, by excluding studies rated as high risk of bias. [file Data_sheet_1.zip › Supplementary Figures/SuppFig24.tiff]

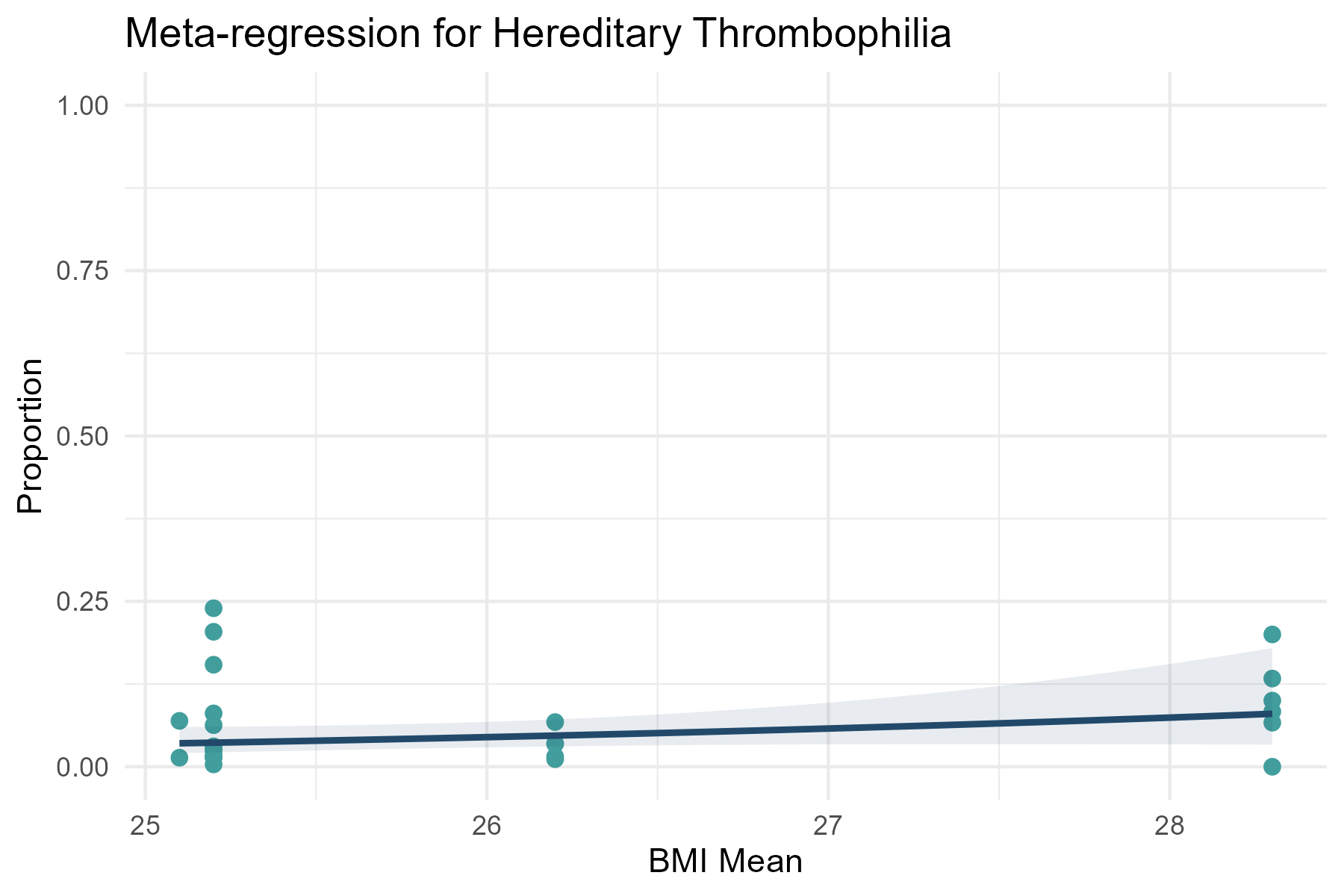

Supplement: SUPPLEMENTARY FIGURE S1 — Sensitivity analyses for the proportion of the major etiological categories of recurrent pregnancy loss, by excluding studies rated as high risk of bias. [file Data_sheet_1.zip › Supplementary Figures/SuppFig25.tiff]

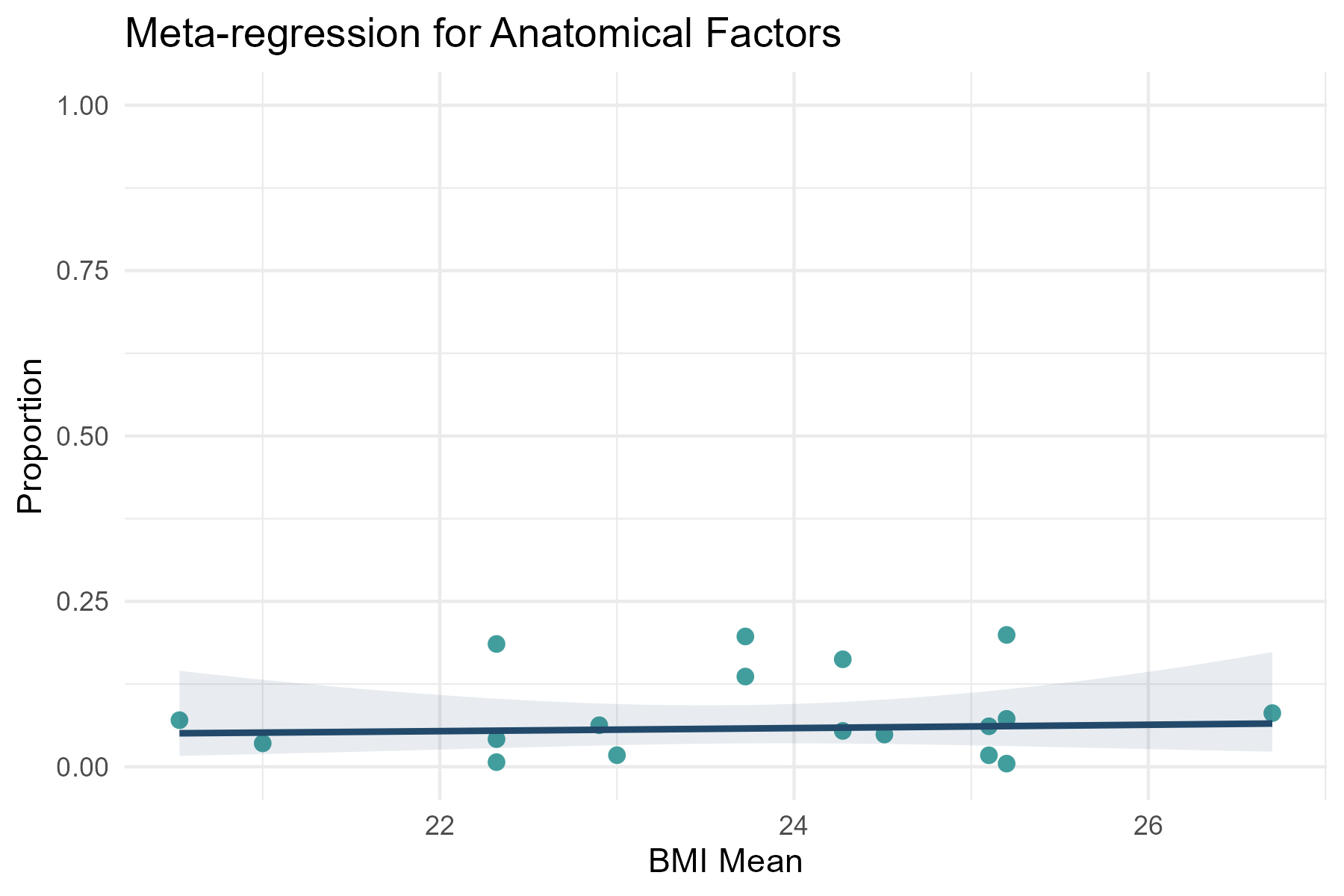

Supplement: SUPPLEMENTARY FIGURE S1 — Sensitivity analyses for the proportion of the major etiological categories of recurrent pregnancy loss, by excluding studies rated as high risk of bias. [file Data_sheet_1.zip › Supplementary Figures/SuppFig26.tiff]

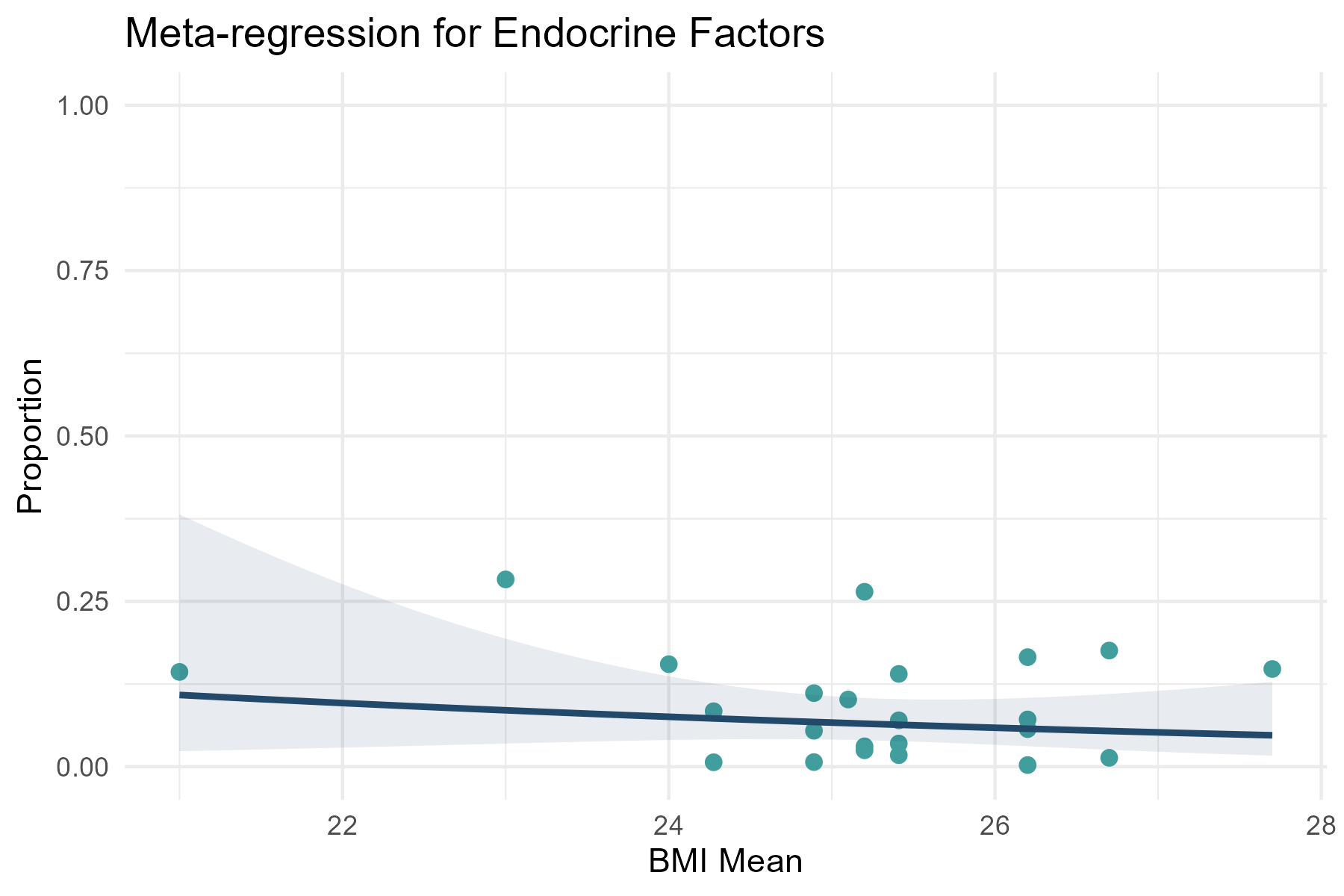

Supplement: SUPPLEMENTARY FIGURE S1 — Sensitivity analyses for the proportion of the major etiological categories of recurrent pregnancy loss, by excluding studies rated as high risk of bias. [file Data_sheet_1.zip › Supplementary Figures/SuppFig27.tiff]

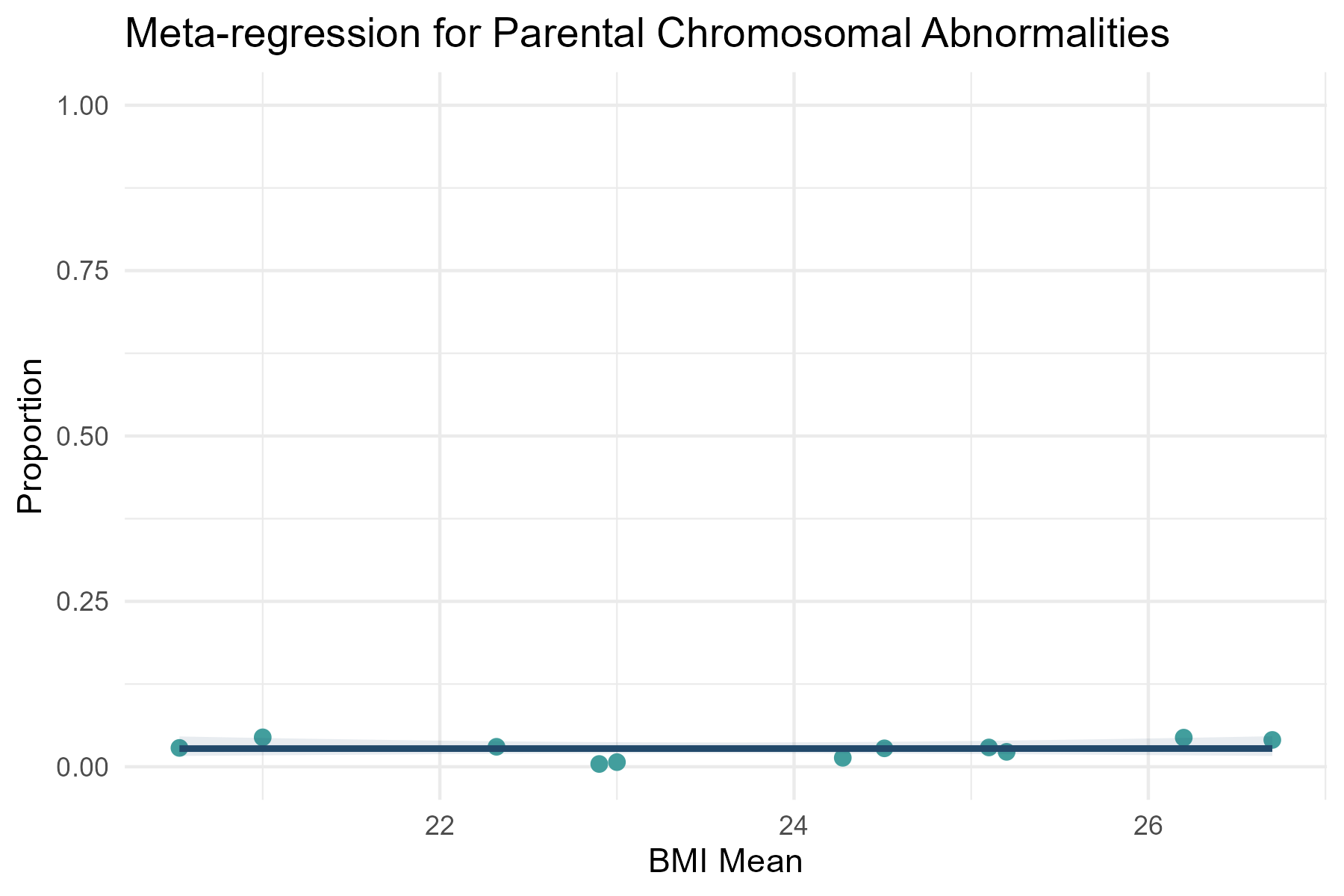

Supplement: SUPPLEMENTARY FIGURE S1 — Sensitivity analyses for the proportion of the major etiological categories of recurrent pregnancy loss, by excluding studies rated as high risk of bias. [file Data_sheet_1.zip › Supplementary Figures/SuppFig28.tiff]

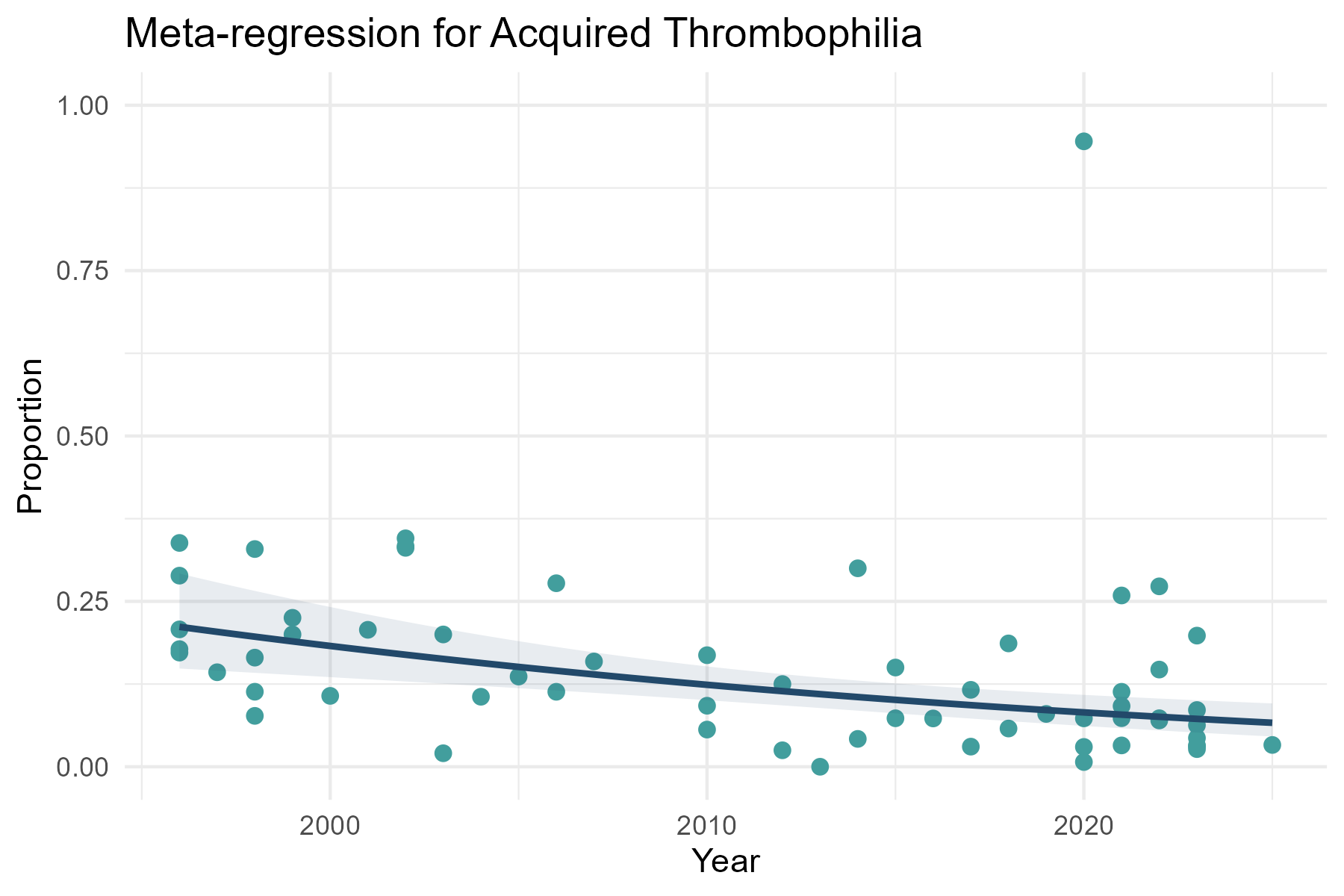

Supplement: SUPPLEMENTARY FIGURE S1 — Sensitivity analyses for the proportion of the major etiological categories of recurrent pregnancy loss, by excluding studies rated as high risk of bias. [file Data_sheet_1.zip › Supplementary Figures/SuppFig29.tiff]

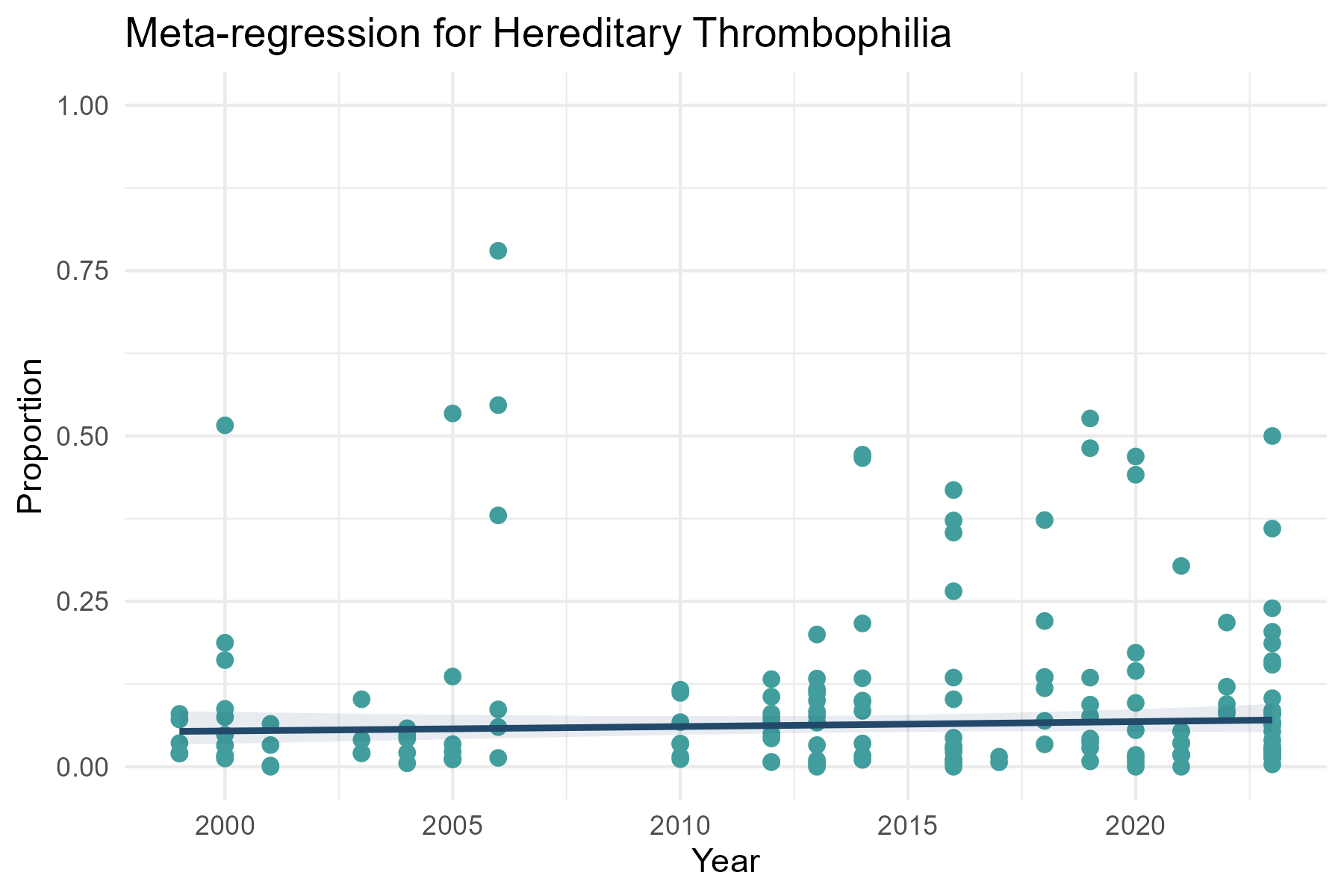

Supplement: SUPPLEMENTARY FIGURE S1 — Sensitivity analyses for the proportion of the major etiological categories of recurrent pregnancy loss, by excluding studies rated as high risk of bias. [file Data_sheet_1.zip › Supplementary Figures/SuppFig30.tiff]

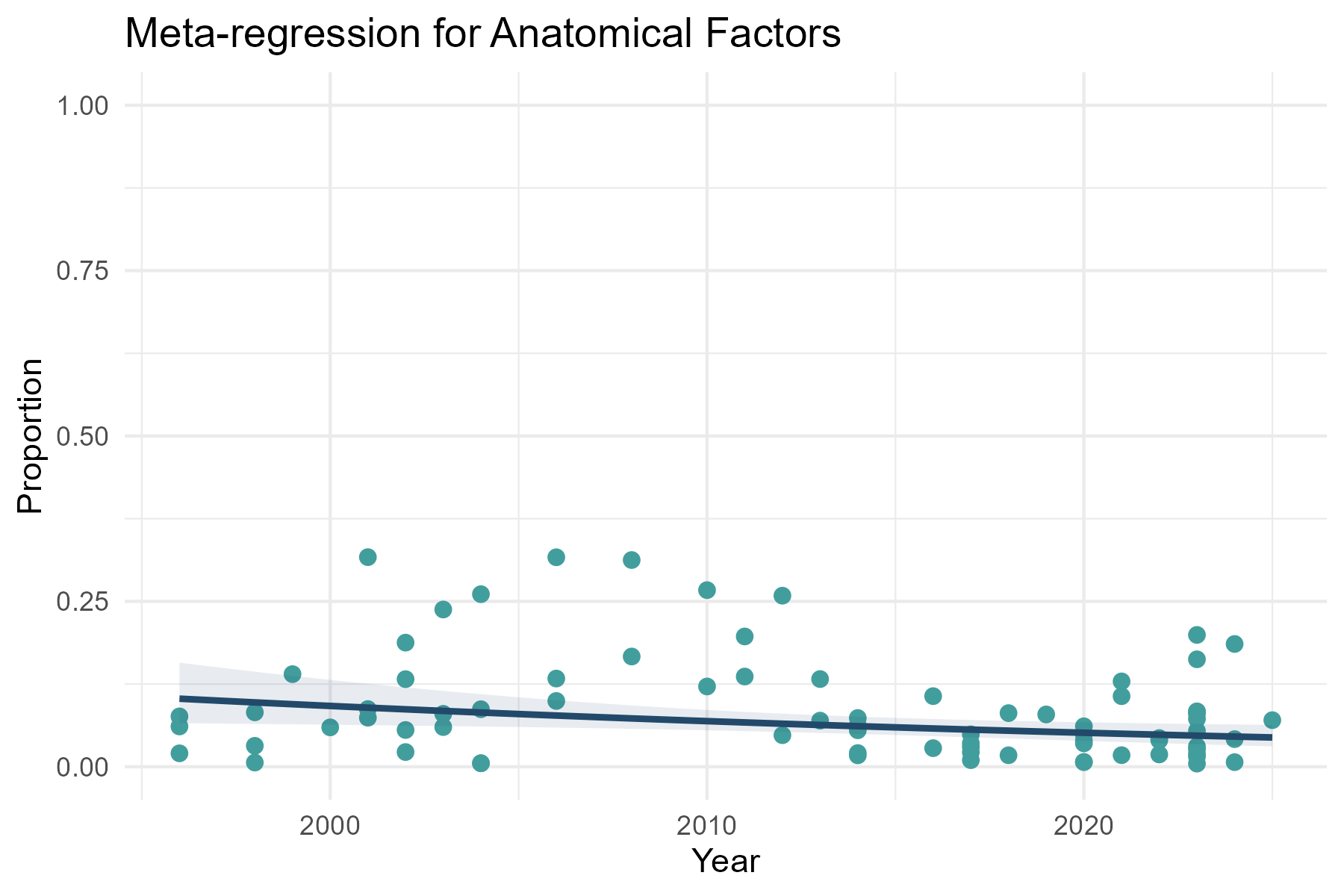

Supplement: SUPPLEMENTARY FIGURE S1 — Sensitivity analyses for the proportion of the major etiological categories of recurrent pregnancy loss, by excluding studies rated as high risk of bias. [file Data_sheet_1.zip › Supplementary Figures/SuppFig31.tiff]

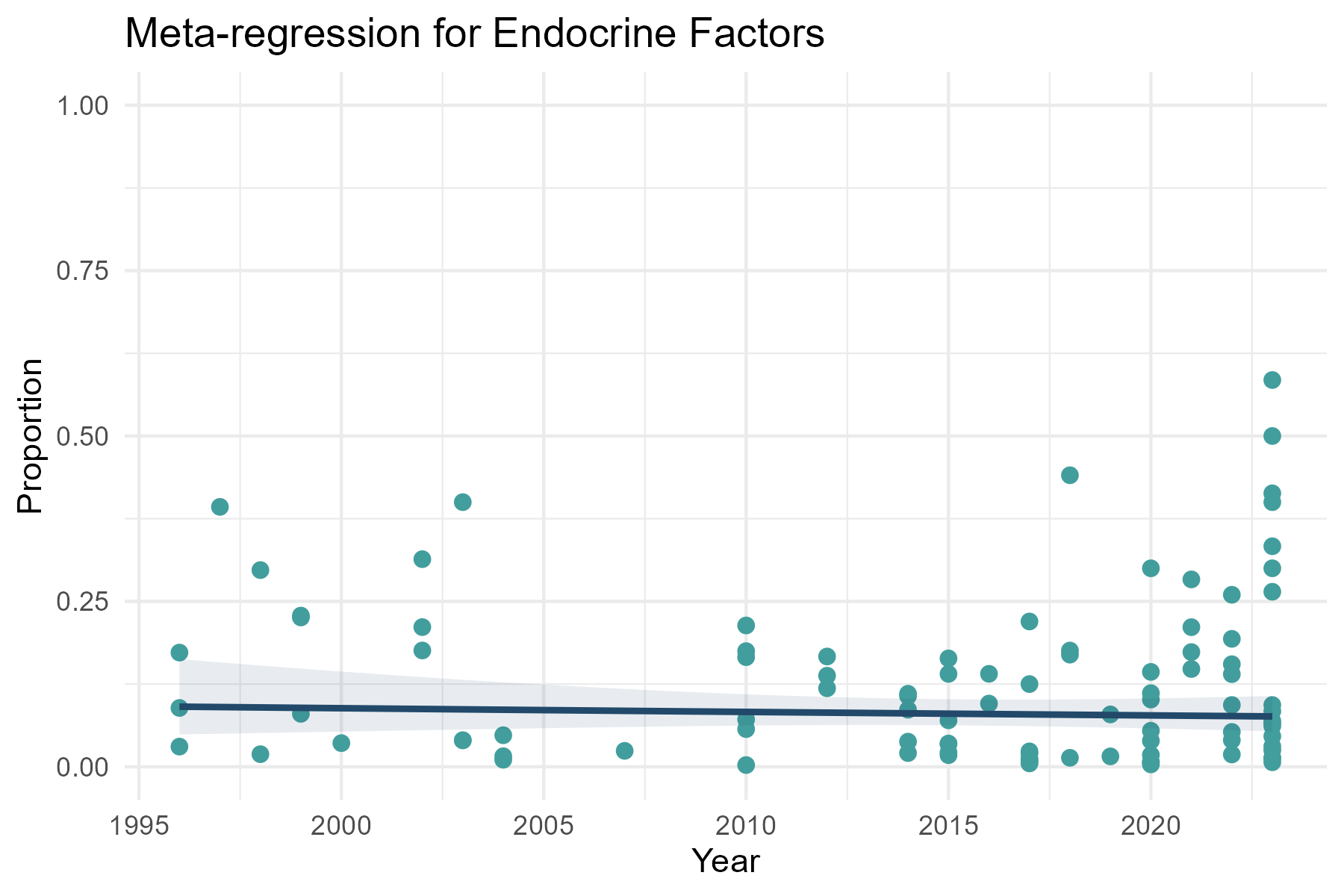

Supplement: SUPPLEMENTARY FIGURE S1 — Sensitivity analyses for the proportion of the major etiological categories of recurrent pregnancy loss, by excluding studies rated as high risk of bias. [file Data_sheet_1.zip › Supplementary Figures/SuppFig32.tiff]

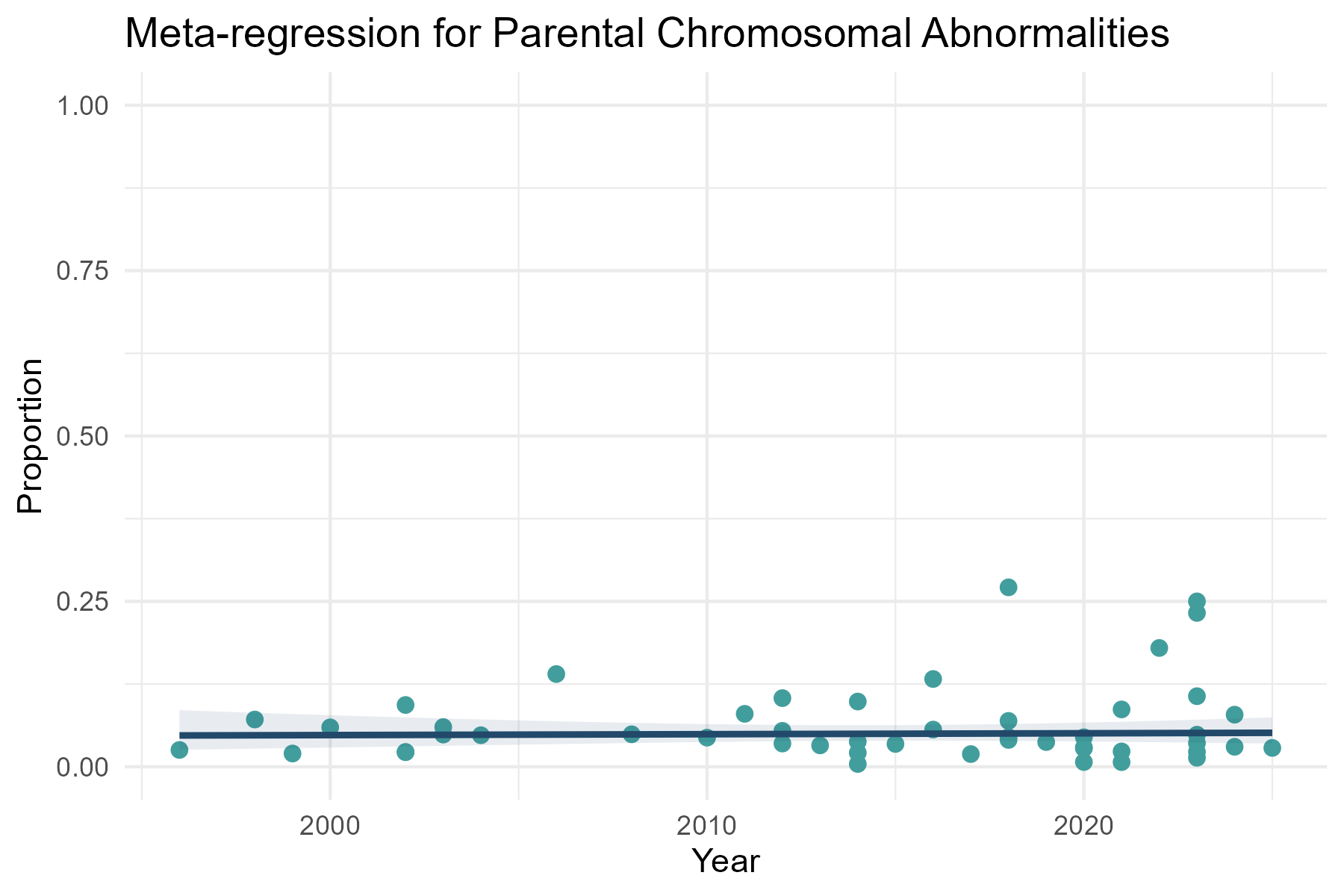

Supplement: SUPPLEMENTARY FIGURE S1 — Sensitivity analyses for the proportion of the major etiological categories of recurrent pregnancy loss, by excluding studies rated as high risk of bias. [file Data_sheet_1.zip › Supplementary Figures/SuppFig33.tiff]

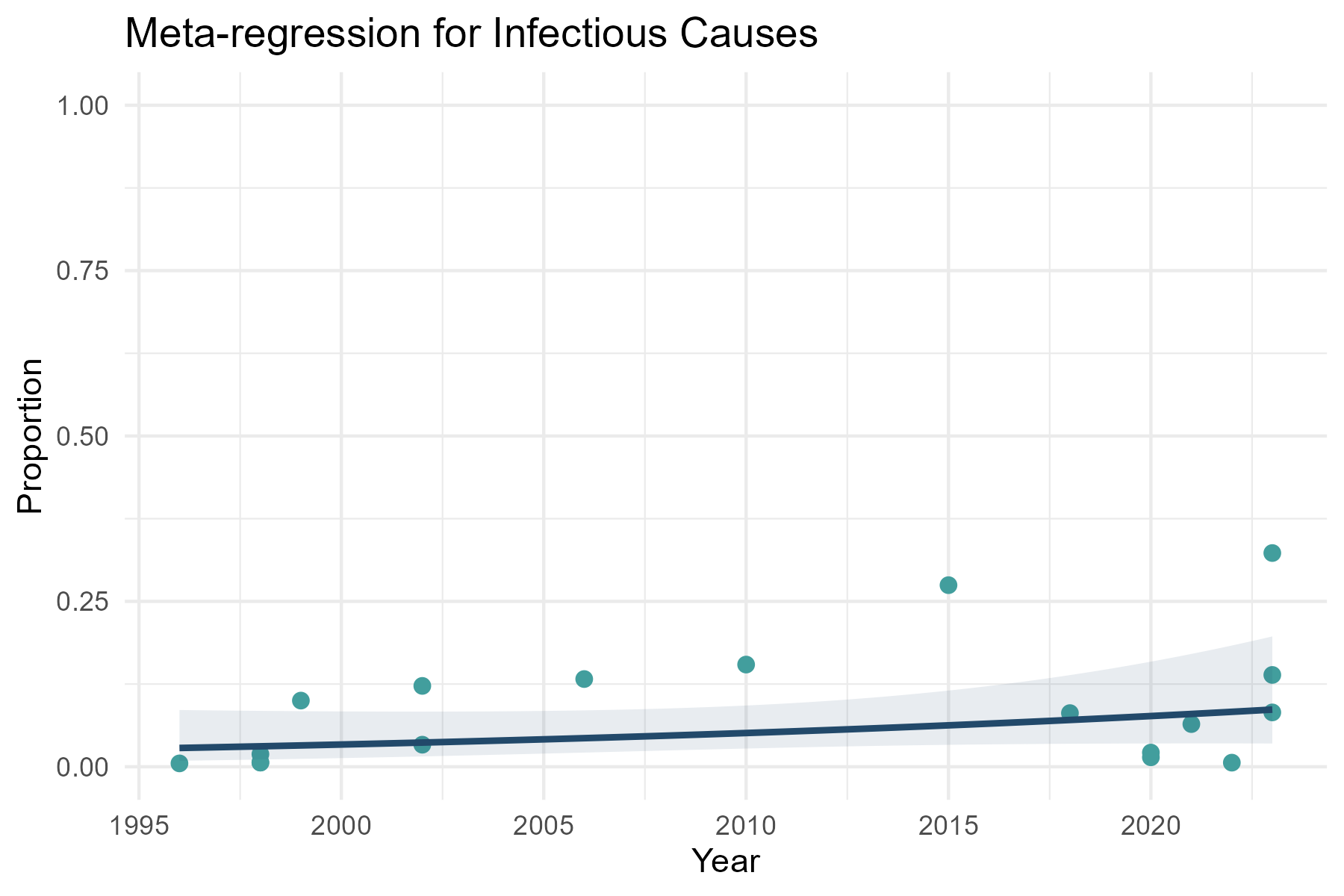

Supplement: SUPPLEMENTARY FIGURE S1 — Sensitivity analyses for the proportion of the major etiological categories of recurrent pregnancy loss, by excluding studies rated as high risk of bias. [file Data_sheet_1.zip › Supplementary Figures/SuppFig34.tiff]

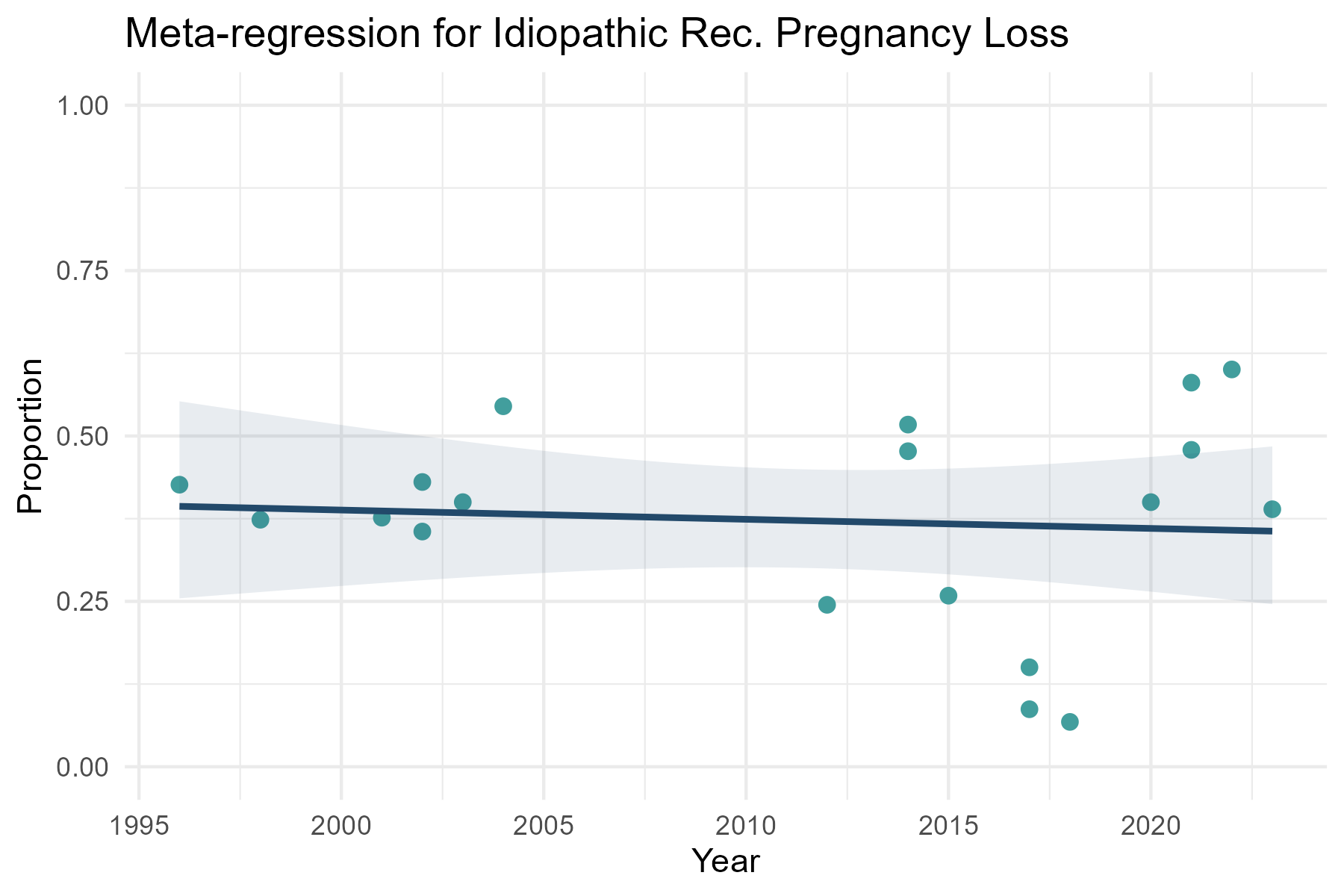

Supplement: SUPPLEMENTARY FIGURE S1 — Sensitivity analyses for the proportion of the major etiological categories of recurrent pregnancy loss, by excluding studies rated as high risk of bias. [file Data_sheet_1.zip › Supplementary Figures/SuppFig35.tiff]
