# Supplementary material for: Recurrent pregnancy loss: systematic review and meta-analysis of overall prevalence and the distribution of major etiological categories
Source: Front Med (Lausanne). 2026 Apr 1;13:1805994. doi: 10.3389/fmed.2026.1805994 (PMC13079578; doi:10.3389/fmed.2026.1805994)
Supplement: Supplementary file 2 [file Data_sheet_2.zip › Supplementary Tables/New Supplementary Table 2.docx]

**Supplementary Table 2.** Retrieval search strategy.

| **Query** | **Search** |
| --- | --- |
| **PubMed** | *Title, Abstract* |
| #1 | Abortion, Habitual[MesH Terms] OR "recurrent miscarriage*"[Title/Abstract] OR "recurrent abortion*"[Title/Abstract] OR "recurrent pregnancy loss*"[Title/Abstract] OR "spontaneous abortion*"[Title/Abstract] OR "recurrent spontaneous abortion*"[Title/Abstract] OR "repeated abortion*"[Title/Abstract] OR "repeated miscarriage*"[Title/Abstract] OR "repetitive miscarriage*"[Title/Abstract] OR "repetitive abortion*"[Title/Abstract] OR "successive abortion*"[Title/Abstract] OR "successive pregnancy loss*"[Title/Abstract] OR "recurrent reproductive failure"[Title/Abstract] OR "pregnancy loss*"[Title/Abstract] OR "Recurrent Spontaneous Miscarriage*"[Title/Abstract] |
| #2 | Prevalence[MesH Terms] OR Incidence[MesH Terms] OR frequenc*[Title/Abstract] OR significan*[Title/Abstract] OR relevan*[Title/Abstract] OR value[Title/Abstract] OR "clinical utility"[Title/Abstract] OR occurrenc*[Title/Abstract] OR likelihood[Title/Abstract] OR Probability[MeSH Terms] OR impact[Title/Abstract] OR Etiology[MeSH Terms] OR Causality[MeSH Terms] OR "etiologic factor*"[Title/Abstract] OR aetiolog*[Title/Abstract] OR "aetiologic* factor*"[Title/Abstract] OR aberration*[Title/Abstract] OR abnormal*[Title/Abstract] OR anomal*[Title/Abstract] OR malformat*[Title/Abstract] OR Congenital Abnormalities[MeSH Terms] OR congenital[Title/Abstract] OR defect*[Title/Abstract] OR Mutation[MeSH Terms] OR Chromosome Aberrations[MeSH Terms] OR chromosomal[Title/Abstract] OR karyotyp*[Title/Abstract] OR Abnormal Karyotype[MeSH Terms] OR cytogenetic[Title/Abstract] OR carrier*[Title/Abstract] OR Translocation, Genetic[MeSH Terms] OR translocat*[Title/Abstract] OR Aneuploidy[MeSH Terms] OR carrier*[Title/Abstract] OR mullerian*[Title/Abstract] OR "uterine abnormalit*"[Title/Abstract] OR "congenital uterine anomal*"[Title/Abstract] OR "uter* anatom*"[Title/Abstract] OR "arcuate uterus"[Title/Abstract] OR Septate Uterus[MeSH Terms] OR (uterus[MeSH Terms] AND (subseptat*[Title/Abstract] OR subsept*[Title/Abstract] OR sept*[Title/Abstract] OR sub-sept*[Title/Abstract])) OR Hysteroscopy[MeSH Terms] OR hysteroscop*[Title/Abstract] OR Myoma[MeSH Terms] OR fibroid*[Title/Abstract] OR "endometrial polyp*"[Title/Abstract] OR "intrauterine adhesion*"[Title/Abstract] or Asherman*[Title/Abstract] OR Gynatresia[MeSH Terms] OR "cervical integrity"[Title/Abstract] OR "cervical insufficiency"[Title/Abstract] OR Antiphospholipid Syndrome[MeSH Terms] OR thrombophil*[Title/Abstract] OR "APC resistance"[Title/Abstract] OR APC-R[Title/Abstract] OR Activated Protein C Resistance[MeSH Terms] OR "Thrombophilia due to Activated Protein C Resistance"[Title/Abstract] OR "factor V Leiden"[Title/Abstract] OR G1691A[Title/Abstract] OR prothrombin*[Title/Abstract] OR Hyperprothrombinemia[Title/Abstract] OR G20210A[Title/Abstract] OR Methylenetetrahydrofolate Reductase[MeSH Terms] OR MTHFR[Title/Abstract] OR C677T[Title/Abstract] OR A1298C[Title/Abstract] OR Hyperhomocysteinemia[MeSH Terms] OR homocystein*[Title/Abstract] OR Factor VIII[MeSH Terms] OR Protein C[MeSH Terms] OR Protein S[MeSH Terms] OR antithrombin[Title/Abstract] OR antiphospho*[Title/Abstract] OR phospho*[Title/Abstract] OR aPL[Title/Abstract] OR Cardiolipins[MeSH Terms] OR Antibodies, Anticardiolipin[MeSH Terms] OR anticardiolipin*[Title/Abstract] OR ACA[Title/Abstract] OR aCL[Title/Abstract] OR CLAb*[Title/Abstract] OR CL-Ab*[Title/Abstract] OR Lupus Coagulation Inhibitor[MeSH Terms] OR "lupus anticoag*"[Title/Abstract] OR beta 2-Glycoprotein I[MeSH Terms] OR beta-2-glycoprotein*[Title/Abstract] OR beta2-glycoprotein*[Title/Abstract] OR abeta2GPI[Title/Abstract] OR beta-2-GPI[Title/Abstract] OR beta2GPI[Title/Abstract] OR beta2-GPI[Title/Abstract] OR Annexins[MeSH Terms] OR antiphosphatidyl*[Title/Abstract] OR phosphatidyl*[Title/Abstract] OR APA[Title/Abstract] OR APSA*[Title/Abstract] OR APS[Title/Abstract] OR anti-PS[Title/Abstract] OR aPE[Title/Abstract] OR (Thyroid[Title/Abstract] AND (Autoimmunity[MeSH Terms] OR autoimmun*[Title/Abstract] OR auto-immun*[Title/Abstract] OR autoantibod*[Title/Abstract] OR auto-antibod*[Title/Abstract] OR auto-Ab*)) OR Hyperthyroidism[MeSH Terms] OR Hypothyroidism[MeSH Terms] OR hyperthyr*[Title/Abstract] OR hypothyr*[Title/Abstract] OR TFT[Title/Abstract] OR TSH[Title/Abstract] OR TRH[Title/Abstract] OR antithyroid*[Title/Abstract] OR thyroglobulin*[Title/Abstract] OR TG[Title/Abstract] OR antiTG*[Title/Abstract] OR TPO[Title/Abstract] OR TPOAb[Title/Abstract] OR thyroperoxidas*[Title/Abstract] OR iodide peroxidase*[Title/Abstract] OR ATA[Title/Abstract] OR antithyroglobulin*[Title/Abstract] OR TgAb[Title/Abstract] OR Thyrotropin[MeSH Terms] OR TRAb[Title/Abstract] OR Polycystic Ovary Syndrome[MeSH Terms] OR PCOS[Title/Abstract] OR "luteal phase defect"[Title/Abstract] OR LPD[Title/Abstract] OR Reproductive Tract Infections[MeSH Terms] OR "genital tract infection*"[Title/Abstract] OR "genital infection*"[Title/Abstract] OR Vaginosis, Bacterial[MeSH Terms] OR "nonspecific vaginitis"[Title/Abstract] OR "chronic endometritis"[Title/Abstract] |
| #3 | #1 AND #2 |
| #4 | #1 AND #2 NOT ("Randomized Controlled Trial*" OR Review* OR "case report*" OR "clinical trial*" OR "meta-analysis") |
| #5 | #1 AND #2 NOT ("Randomized Controlled Trial*" OR Review* OR "case report*" OR "clinical trial*" OR "meta-analysis")  Filters: Humans |
| #6 | #1 AND #2 NOT ("Randomized Controlled Trial*" OR Review* OR "case report*" OR "clinical trial*" OR "meta-analysis")  Filters: Humans, 1995-2024 |
| **Scopus** | *Article title, abstract, keywords* |
| #1 | "recurrent miscarriage*" OR "recurrent abortion*" OR "recurrent pregnancy loss*" OR "spontaneous abortion*" OR "recurrent spontaneous abortion*" OR "repeated abortion*" OR "repeated miscarriage*" OR "repetitive miscarriage*" OR "repetitive abortion*"OR "successive abortion*" OR "successive pregnancy loss*" OR "recurrent reproductive failure" OR "pregnancy loss*" OR "Recurrent Spontaneous Miscarriage*" |
| #2 | prevalence OR incidence OR frequenc* OR significan* OR relevan* OR value OR "clinical utility" OR occurrenc* OR likelihood OR probability OR impact OR causality OR etiolog* OR aetiolog* OR aberration* OR anomal* OR malformat* OR congenital OR defect* OR mutation* OR “chromosome aberration*” OR chromosomal OR karyotyp* OR cytogenetic OR carrier* OR translocat* OR aneuploid* OR carrier* OR mullerian* OR "uterine abnormalit*" OR "congenital uterine anomal*" OR "uter* anatom*" OR "arcuate uterus" OR “sept* uterus” OR “subseptat* uterus” OR “sub-sept* uterus” OR hysteroscop* OR myoma* OR fibroid* OR "endometrial polyp*" OR "intrauterine adhesion*" OR Asherman* OR gynatresia OR "cervical integrity" OR "cervical insufficiency" OR “antiphospholipid syndrome” OR thrombophil* OR "APC resistance" OR APC-R OR “activated protein C resistance” OR "thrombophilia due to activated protein C resistance" OR "factor V Leiden" OR G1691A OR prothrombin* OR hyperprothrombinemia OR G20210A OR “mthylenetetrahydrofolate reductase” OR MTHFR OR C677T OR A1298C OR hyperhomocysteinemia OR homocystein* OR “Factor VIII” OR “Protein C” OR “Protein S” OR antithrombin OR antiphospho* OR phospho* OR aPL OR cardiolipins OR anticardiolipin* OR ACA OR aCL OR CLAb* OR CL-Ab* OR “lupus coagulation inhibitor” OR "lupus anticoag*" OR “beta 2-Glycoprotein I” OR beta-2-glycoprotein* OR beta2-glycoprotein* OR abeta2GPI OR beta-2-GPI OR beta2GPI OR beta2-GPI OR annexins OR antiphosphatidyl* OR phosphatidyl* OR APA OR APSA* OR APS OR anti-PS OR aPE OR (Thyroid AND (autoimmun* OR auto-immun* OR autoantibod* OR auto-antibod* OR auto-Ab*)) OR hyperthyr* OR hypothyr* OR TFT OR TSH OR TRH OR antithyroid* OR thyroglobulin* OR TG OR antiTG* OR TPO OR TPOAb OR thyroperoxidas* OR iodide peroxidase* OR ATA OR antithyroglobulin* OR TgAb OR thyrotropin OR TRAb OR “polycystic ovary syndrome” OR PCOS OR "luteal phase defect" OR LPD OR “reproductive tract infection*” OR "genital tract infection*" OR "genital infection*" OR “bacterial vaginosis” OR "nonspecific vaginitis" OR "chronic endometritis" |
| #3 | #1 AND #2 |
| #4 | #1 AND #2 AND NOT animal* |
| #5 | #1 AND #2 AND NOT animal* AND NOT ("Randomized Controlled Trial*" OR Review* OR "case report*" OR "clinical trial*" OR "meta-analysis") |
| #6 | #5 Filters. 1995-2024 |
| **Web of Science** | *TOPIC - title, abstract, author keywords, and Keywords Plus* |
| #1 | "recurrent miscarriage*" OR "recurrent abortion*" OR "recurrent pregnancy loss*" OR "spontaneous abortion*" OR "recurrent spontaneous abortion*" OR "repeated abortion*" OR "repeated miscarriage*" OR "repetitive miscarriage*" OR "repetitive abortion*"OR "successive abortion*" OR "successive pregnancy loss*" OR "recurrent reproductive failure" OR "pregnancy loss*" OR "Recurrent Spontaneous Miscarriage*" |
| #2 | prevalence OR incidence OR frequenc* OR significan* OR relevan* OR value OR "clinical utility" OR occurrenc* OR likelihood OR probability OR impact OR causality OR etiolog* OR aetiolog* OR aberration* OR anomal* OR malformat* OR congenital OR defect* OR mutation* OR “chromosome aberration*” OR chromosomal OR karyotyp* OR cytogenetic OR carrier* OR translocat* OR aneuploid* OR carrier* OR mullerian* OR "uterine abnormalit*" OR "congenital uterine anomal*" OR "uter* anatom*" OR "arcuate uterus" OR “sept* uterus” OR “subseptat* uterus” OR “sub-sept* uterus” OR hysteroscop* OR myoma* OR fibroid* OR "endometrial polyp*" OR "intrauterine adhesion*" OR Asherman* OR gynatresia OR "cervical integrity" OR "cervical insufficiency" OR “antiphospholipid syndrome” OR thrombophil* OR "APC resistance" OR APC-R OR “activated protein C resistance” OR "thrombophilia due to activated protein C resistance" OR "factor V Leiden" OR G1691A OR prothrombin* OR hyperprothrombinemia OR G20210A OR “mthylenetetrahydrofolate reductase” OR MTHFR OR C677T OR A1298C OR hyperhomocysteinemia OR homocystein* OR “Factor VIII” OR “Protein C” OR “Protein S” OR antithrombin OR antiphospho* OR phospho* OR aPL OR cardiolipins OR anticardiolipin* OR ACA OR aCL OR CLAb* OR CL-Ab* OR “lupus coagulation inhibitor” OR "lupus anticoag*" OR “beta 2-Glycoprotein I” OR beta-2-glycoprotein* OR beta2-glycoprotein* OR abeta2GPI OR beta-2-GPI OR beta2GPI OR beta2-GPI OR annexins OR antiphosphatidyl* OR phosphatidyl* OR APA OR APSA* OR APS OR anti-PS OR aPE OR (Thyroid AND (autoimmun* OR auto-immun* OR autoantibod* OR auto-antibod* OR auto-Ab*)) OR hyperthyr* OR hypothyr* OR TFT OR TSH OR TRH OR antithyroid* OR thyroglobulin* OR TG OR antiTG* OR TPO OR TPOAb OR thyroperoxidas* OR iodide peroxidase* OR ATA OR antithyroglobulin* OR TgAb OR thyrotropin OR TRAb OR “polycystic ovary syndrome” OR PCOS OR "luteal phase defect" OR LPD OR "immune factor*" OR HLA OR cytokine* OR “natural killer cell*” OR "NK cell*" OR “reproductive tract infection*” OR "genital tract infection*" OR "genital infection*" OR “bacterial vaginosis” OR "nonspecific vaginitis" OR "chronic endometritis" |
| #3 | #1 AND #2 |
| #4 | #1 AND #2 NOT animal* |
| #5 | #1 AND #2 NOT animal* NOT ("randomized controlled trial" OR "observational study") |
| #6 | #1 AND #2 NOT animal* NOT ("Randomized Controlled Trial*" OR Review* OR "case report*" OR "clinical trial*" OR "meta-analysis")  Filters: 1995-2024 |
| **Cochrane Library** | *Title, abstract, keywords* |
| #1 | “recurrent miscarriage” OR “recurrent abortion” OR “recurrent pregnancy loss” OR “spontaneous abortion” OR “recurrent spontaneous abortion” OR “repeated abortion” OR “repeated miscarriage” OR “repetitive miscarriage” OR “repetitive abortion” OR “successive abortion” OR “successive pregnancy loss” OR “recurrent reproductive failure” OR “pregnancy loss” OR "Recurrent Spontaneous Miscarriage" |
| #2 | prevalence OR incidence OR frequenc* OR significan* OR relevan* OR value OR "clinical utility" OR occurrenc* OR likelihood OR probability OR impact OR causality OR etiolog* OR aetiolog* OR aberration* OR anomal* OR malformat* OR congenital OR defect* OR mutation* OR “chromosome aberration” OR chromosomal OR karyotype* OR cytogenetic OR carrier* OR translocate OR aneuploid* OR carrier* OR mullerian* OR “uterine abnormality” OR "congenital uterine anomaly" OR "uterine anatomy" OR "arcuate uterus" OR “septate uterus” OR “subseptate uterus” OR “sub-septate uterus” OR hysteroscop* OR myoma* OR fibroid* OR "endometrial polyp" OR "intrauterine adhesion" OR Asherman* OR gynatresia OR "cervical integrity" OR "cervical insufficiency" OR “antiphospholipid syndrome” OR thrombophil* OR "APC resistance" OR APC-R OR “activated protein C resistance” OR "thrombophilia due to activated protein C resistance" OR "factor V Leiden" OR G1691A OR prothrombin* OR hyperprothrombinemia OR G20210A OR “mthylenetetrahydrofolate reductase” OR MTHFR OR C677T OR A1298C OR hyperhomocysteinemia OR homocystein* OR “Factor VIII” OR “Protein C” OR “Protein S” OR antithrombin OR antiphospho* OR phospho* OR aPL OR cardiolipins OR anticardiolipin* OR ACA OR aCL OR CLAb* OR CL-Ab* OR “lupus coagulation inhibitor” OR "lupus anticoagulant" OR "beta 2-Glycoprotein" OR "beta-2-glycoprotein" OR "beta2-glycoprotein" OR abeta2GPI OR "beta-2-GPI" OR beta2GPI OR "beta2-GPI" OR annexins OR antiphosphatidyl* OR phosphatidyl* OR APA OR APSA* OR APS OR "anti-PS" OR aPE OR (Thyroid AND (autoimmun* OR auto-immun* OR autoantibod* OR auto-antibodies OR auto-Ab)) OR hyperthyr* OR hypothyr* OR TFT OR TSH OR TRH OR antithyroid* OR thyroglobulin* OR TG OR antiTG OR TPO OR TPOAb OR thyroperoxidas* OR “iodide peroxidase” OR “ATA” OR antithyroglobulin* OR TgAb OR thyrotropin OR TRAb OR “polycystic ovary syndrome” OR PCOS OR “luteal phase defect” OR LPD OR “genital tract infection” OR “genital infection” OR “bacterial vaginosis” OR "nonspecific vaginitis" OR "chronic endometritis" |
| #3 | #1 AND #2 |
| #4 | #1 AND #2 NOT animal* |
| #5 | #1 AND #2 NOT animal* NOT ("Randomized Controlled Trial" OR Review OR "case report" OR "clinical trial" OR "meta-analysis") |
| #6 | #1 AND #2 NOT animal* NOT ("Randomized Controlled Trial" OR Review OR "case report" OR "clinical trial" OR "meta-analysis")  Filters: 1995-2024 |
| **EMBASE** | ***Title & Abstract*** |
| #1 | "recurrent miscarriage*" OR "recurrent abortion*" OR "recurrent pregnancy loss*" OR "spontaneous abortion*" OR "recurrent spontaneous abortion*" OR "repeated abortion*" OR "repeated miscarriage*" OR "repetitive miscarriage*" OR "repetitive abortion*"OR "successive abortion*" OR "successive pregnancy loss*" OR "recurrent reproductive failure" OR "pregnancy loss*" OR "Recurrent Spontaneous Miscarriage*" |
| #2 | prevalence OR incidence OR frequenc* OR significan* OR relevan* OR value OR "clinical utility" OR occurrenc* OR likelihood OR probability OR impact OR causality OR etiolog* OR aetiolog* OR aberration* OR anomal* OR malformat* OR congenital OR defect* OR mutation* OR “chromosome aberration*” OR chromosomal OR karyotyp* OR cytogenetic OR carrier* OR translocat* OR aneuploid* OR carrier* OR mullerian* OR "uterine abnormalit*" OR "congenital uterine anomal*" OR "uter* anatom*" OR "arcuate uterus" OR “sept* uterus” OR “subseptat* uterus” OR “sub-sept* uterus” OR hysteroscop* OR myoma* OR fibroid* OR "endometrial polyp*" OR "intrauterine adhesion*" OR Asherman* OR gynatresia OR "cervical integrity" OR "cervical insufficiency" OR “antiphospholipid syndrome” OR thrombophil* OR "APC resistance" OR APC-R OR “activated protein C resistance” OR "thrombophilia due to activated protein C resistance" OR "factor V Leiden" OR G1691A OR prothrombin* OR hyperprothrombinemia OR G20210A OR “mthylenetetrahydrofolate reductase” OR MTHFR OR C677T OR A1298C OR hyperhomocysteinemia OR homocystein* OR “Factor VIII” OR “Protein C” OR “Protein S” OR antithrombin OR antiphospho* OR phospho* OR aPL OR cardiolipins OR anticardiolipin* OR ACA OR aCL OR CLAb* OR CL-Ab* OR “lupus coagulation inhibitor” OR "lupus anticoag*" OR “beta 2-Glycoprotein I” OR beta-2-glycoprotein* OR beta2-glycoprotein* OR abeta2GPI OR beta-2-GPI OR beta2GPI OR beta2-GPI OR annexins OR antiphosphatidyl* OR phosphatidyl* OR APA OR APSA* OR APS OR anti-PS OR aPE OR (Thyroid AND (autoimmun* OR auto-immun* OR autoantibod* OR auto-antibod* OR auto-Ab*)) OR hyperthyr* OR hypothyr* OR TFT OR TSH OR TRH OR antithyroid* OR thyroglobulin* OR TG OR antiTG* OR TPO OR TPOAb OR thyroperoxidas* OR iodide peroxidase* OR ATA OR antithyroglobulin* OR TgAb OR thyrotropin OR TRAb OR “polycystic ovary syndrome” OR PCOS OR "luteal phase defect" OR LPD OR “reproductive tract infection*” OR "genital tract infection*" OR "genital infection*" OR “bacterial vaginosis” OR "nonspecific vaginitis" OR "chronic endometritis" |
| #3 | #1 AND #2 |
| #4 | #1 AND #2 NOT ('animal'/exp NOT 'human'/exp) |
| #5 | #1 AND #2 NOT ('animal'/exp NOT 'human'/exp) NOT ("Randomized Controlled Trial*" OR Review* OR "case report*" OR "clinical trial*" OR "meta-analysis") |
