# Supplementary material for: Recurrent pregnancy loss: systematic review and meta-analysis of overall prevalence and the distribution of major etiological categories
Source: Front Med (Lausanne). 2026 Apr 1;13:1805994. doi: 10.3389/fmed.2026.1805994 (PMC13079578; doi:10.3389/fmed.2026.1805994)
Supplement: Supplementary file 2 [file Data_sheet_2.zip › Supplementary Tables/NEW Supplementary Table 4.docx]

**Supplementary Table 4.** Pairwise comparisons between the different definitions of hereditary thrombophilia**.**

| Subgroup 1 | Subgroup 2 | *P* value^a^ |
| --- | --- | --- |
| Protein C deficiency | Protein S deficiency | > 0.99 |
| Protein C deficiency | Antithrombin III deficiency | > 0.99 |
| Protein C deficiency | APC resistance | > 0.99 |
| Protein C deficiency | Factor V Leiden heterozygous | 0.02 |
| Protein C deficiency | Factor V Leiden homozygous | > 0.99 |
| Protein C deficiency | Prothrombin G20210A heterozygous | > 0.99 |
| Protein C deficiency | Prothrombin G20210A homozygous | 0.02 |
| Protein C deficiency | Compound heterozygous Factor V Leiden & Prothrombin G20210A genetic variations | > 0.99 |
| Protein C deficiency | Factor XII deficiency | > 0.99 |
| Protein C deficiency | Hyperhomocysteinemia | > 0.99 |
| Protein C deficiency | MTHFR C677T heterozygous | < 0.001 |
| Protein C deficiency | MTHFR C677T homozygous | 0.56 |
| Protein C deficiency | MTHFR A1298C heterozygous | < 0.001 |
| Protein C deficiency | MTHFR A1298C homozygous | 0.002 |
| Protein C deficiency | PAI-1 4G/5G heterozygous | > 0.99 |
| Protein C deficiency | PAI-1 4G/5G homozygous | 0.14 |
| Protein S deficiency | Antithrombin III deficiency | > 0.99 |
| Protein S deficiency | APC resistance | > 0.99 |
| Protein S deficiency | Factor V Leiden heterozygous | > 0.99 |
| Protein S deficiency | Factor V Leiden homozygous | > 0.99 |
| Protein S deficiency | Prothrombin G20210A heterozygous | > 0.99 |
| Protein S deficiency | Prothrombin G20210A homozygous | < 0.001 |
| Protein S deficiency | Compound heterozygous Factor V Leiden & Prothrombin G20210A genetic variations | > 0.99 |
| Protein S deficiency | Factor XII deficiency | > 0.99 |
| Protein S deficiency | Hyperhomocysteinemia | > 0.99 |
| Protein S deficiency | MTHFR C677T heterozygous | < 0.001 |
| Protein S deficiency | MTHFR C677T homozygous | > 0.99 |
| Protein S deficiency | MTHFR A1298C heterozygous | < 0.001 |
| Protein S deficiency | MTHFR A1298C homozygous | 0.99 |
| Protein S deficiency | PAI-1 4G/5G heterozygous | > 0.99 |
| Protein S deficiency | PAI-1 4G/5G homozygous | > 0.99 |
| Antithrombin III deficiency | APC resistance | > 0.99 |
| Antithrombin III deficiency | Factor V Leiden heterozygous | 0.34 |
| Antithrombin III deficiency | Factor V Leiden homozygous | > 0.99 |
| Antithrombin III deficiency | Prothrombin G20210A heterozygous | > 0.99 |
| Antithrombin III deficiency | Prothrombin G20210A homozygous | 0.35 |
| Antithrombin III deficiency | Compound heterozygous Factor V Leiden & Prothrombin G20210A genetic variations | > 0.99 |
| Antithrombin III deficiency | Factor XII deficiency | > 0.99 |
| Antithrombin III deficiency | Hyperhomocysteinemia | > 0.99 |
| Antithrombin III deficiency | MTHFR C677T heterozygous | < 0.001 |
| Antithrombin III deficiency | MTHFR C677T homozygous | > 0.99 |
| Antithrombin III deficiency | MTHFR A1298C heterozygous | < 0.001 |
| Antithrombin III deficiency | MTHFR A1298C homozygous | 0.046 |
| Antithrombin III deficiency | PAI-1 4G/5G heterozygous | > 0.99 |
| Antithrombin III deficiency | PAI-1 4G/5G homozygous | 0.67 |
| APC resistance | Factor V Leiden heterozygous | > 0.99 |
| APC resistance | Factor V Leiden homozygous | > 0.99 |
| APC resistance | Prothrombin G20210A heterozygous | > 0.99 |
| APC resistance | Prothrombin G20210A homozygous | < 0.001 |
| APC resistance | Compound heterozygous Factor V Leiden & Prothrombin G20210A genetic variations | > 0.99 |
| APC resistance | Factor XII deficiency | > 0.99 |
| APC resistance | Hyperhomocysteinemia | > 0.99 |
| APC resistance | MTHFR C677T heterozygous | < 0.001 |
| APC resistance | MTHFR C677T homozygous | > 0.99 |
| APC resistance | MTHFR A1298C heterozygous | < 0.001 |
| APC resistance | MTHFR A1298C homozygous | 0.68 |
| APC resistance | PAI-1 4G/5G heterozygous | > 0.99 |
| APC resistance | PAI-1 4G/5G homozygous | > 0.99 |
| Factor V Leiden heterozygous | Factor V Leiden homozygous | 0.03 |
| Factor V Leiden heterozygous | Prothrombin G20210A heterozygous | > 0.99 |
| Factor V Leiden heterozygous | Prothrombin G20210A homozygous | < 0.001 |
| Factor V Leiden heterozygous | Compound heterozygous Factor V Leiden & Prothrombin G20210A genetic variations | > 0.99 |
| Factor V Leiden heterozygous | Factor XII deficiency | > 0.99 |
| Factor V Leiden heterozygous | Hyperhomocysteinemia | > 0.99 |
| Factor V Leiden heterozygous | MTHFR C677T heterozygous | < 0.001 |
| Factor V Leiden heterozygous | MTHFR C677T homozygous | > 0.99 |
| Factor V Leiden heterozygous | MTHFR A1298C heterozygous | < 0.001 |
| Factor V Leiden heterozygous | MTHFR A1298C homozygous | > 0.99 |
| Factor V Leiden heterozygous | PAI-1 4G/5G heterozygous | > 0.99 |
| Factor V Leiden heterozygous | PAI-1 4G/5G homozygous | > 0.99 |
| Factor V Leiden homozygous | Prothrombin G20210A heterozygous | > 0.99 |
| Factor V Leiden homozygous | Prothrombin G20210A homozygous | > 0.99 |
| Factor V Leiden homozygous | Compound heterozygous Factor V Leiden & Prothrombin G20210A genetic variations | > 0.99 |
| Factor V Leiden homozygous | Factor XII deficiency | 0.60 |
| Factor V Leiden homozygous | Hyperhomocysteinemia | > 0.99 |
| Factor V Leiden homozygous | MTHFR C677T heterozygous | < 0.001 |
| Factor V Leiden homozygous | MTHFR C677T homozygous | 0.24 |
| Factor V Leiden homozygous | MTHFR A1298C heterozygous | < 0.001 |
| Factor V Leiden homozygous | MTHFR A1298C homozygous | 0.003 |
| Factor V Leiden homozygous | PAI-1 4G/5G heterozygous | 0.99 |
| Factor V Leiden homozygous | PAI-1 4G/5G homozygous | 0.07 |
| Prothrombin G20210A heterozygous | Prothrombin G20210A homozygous | < 0.001 |
| Prothrombin G20210A heterozygous | Compound heterozygous Factor V Leiden & Prothrombin G20210A genetic variations | > 0.99 |
| Prothrombin G20210A heterozygous | Factor XII deficiency | > 0.99 |
| Prothrombin G20210A heterozygous | Hyperhomocysteinemia | > 0.99 |
| Prothrombin G20210A heterozygous | MTHFR C677T heterozygous | < 0.001 |
| Prothrombin G20210A heterozygous | MTHFR C677T homozygous | > 0.99 |
| Prothrombin G20210A heterozygous | MTHFR A1298C heterozygous | < 0.001 |
| Prothrombin G20210A heterozygous | MTHFR A1298C homozygous | 0.55 |
| Prothrombin G20210A heterozygous | PAI-1 4G/5G heterozygous | > 0.99 |
| Prothrombin G20210A heterozygous | PAI-1 4G/5G homozygous | > 0.99 |
| Prothrombin G20210A homozygous | Compound heterozygous Factor V Leiden & Prothrombin G20210A genetic variations | > 0.99 |
| Prothrombin G20210A homozygous | Factor XII deficiency | < 0.001 |
| Prothrombin G20210A homozygous | Hyperhomocysteinemia | 0.003 |
| Prothrombin G20210A homozygous | MTHFR C677T heterozygous | < 0.001 |
| Prothrombin G20210A homozygous | MTHFR C677T homozygous | < 0.001 |
| Prothrombin G20210A homozygous | MTHFR A1298C heterozygous | < 0.001 |
| Prothrombin G20210A homozygous | MTHFR A1298C homozygous | < 0.001 |
| Prothrombin G20210A homozygous | PAI-1 4G/5G heterozygous | 0.08 |
| Prothrombin G20210A homozygous | PAI-1 4G/5G homozygous | < 0.001 |
| Compound heterozygous Factor V Leiden & Prothrombin G20210A genetic variations | Factor XII deficiency | > 0.99 |
| Compound heterozygous Factor V Leiden & Prothrombin G20210A genetic variations | Hyperhomocysteinemia | > 0.99 |
| Compound heterozygous Factor V Leiden & Prothrombin G20210A genetic variations | MTHFR C677T heterozygous | > 0.99 |
| Compound heterozygous Factor V Leiden & Prothrombin G20210A genetic variations | MTHFR C677T homozygous | > 0.99 |
| Compound heterozygous Factor V Leiden & Prothrombin G20210A genetic variations | MTHFR A1298C heterozygous | > 0.99 |
| Compound heterozygous Factor V Leiden & Prothrombin G20210A genetic variations | MTHFR A1298C homozygous | > 0.99 |
| Compound heterozygous Factor V Leiden & Prothrombin G20210A genetic variations | PAI-1 4G/5G heterozygous | > 0.99 |
| Compound heterozygous Factor V Leiden & Prothrombin G20210A genetic variations | PAI-1 4G/5G homozygous | > 0.99 |
| Factor XII deficiency | Hyperhomocysteinemia | > 0.99 |
| Factor XII deficiency | MTHFR C677T heterozygous | 0.003 |
| Factor XII deficiency | MTHFR C677T homozygous | > 0.99 |
| Factor XII deficiency | MTHFR A1298C heterozygous | < 0.001 |
| Factor XII deficiency | MTHFR A1298C homozygous | > 0.99 |
| Factor XII deficiency | PAI-1 4G/5G heterozygous | > 0.99 |
| Factor XII deficiency | PAI-1 4G/5G homozygous | > 0.99 |
| Hyperhomocysteinemia | MTHFR C677T heterozygous | 0.06 |
| Hyperhomocysteinemia | MTHFR C677T homozygous | > 0.99 |
| Hyperhomocysteinemia | MTHFR A1298C heterozygous | 0.002 |
| Hyperhomocysteinemia | MTHFR A1298C homozygous | > 0.99 |
| Hyperhomocysteinemia | PAI-1 4G/5G heterozygous | > 0.99 |
| Hyperhomocysteinemia | PAI-1 4G/5G homozygous | > 0.99 |
| MTHFR C677T heterozygous | MTHFR C677T homozygous | < 0.001 |
| MTHFR C677T heterozygous | MTHFR A1298C heterozygous | > 0.99 |
| MTHFR C677T heterozygous | MTHFR A1298C homozygous | 0.032 |
| MTHFR C677T heterozygous | PAI-1 4G/5G heterozygous | > 0.99 |
| MTHFR C677T heterozygous | PAI-1 4G/5G homozygous | 0.001 |
| MTHFR C677T homozygous | MTHFR A1298C heterozygous | < 0.001 |
| MTHFR C677T homozygous | MTHFR A1298C homozygous | > 0.99 |
| MTHFR C677T homozygous | PAI-1 4G/5G heterozygous | > 0.99 |
| MTHFR C677T homozygous | PAI-1 4G/5G homozygous | > 0.99 |
| MTHFR A1298C heterozygous | MTHFR A1298C homozygous | 0.001 |
| MTHFR A1298C heterozygous | PAI-1 4G/5G heterozygous | > 0.99 |
| MTHFR A1298C heterozygous | PAI-1 4G/5G homozygous | < 0.001 |
| MTHFR A1298C homozygous | PAI-1 4G/5G heterozygous | > 0.99 |
| MTHFR A1298C homozygous | PAI-1 4G/5G homozygous | > 0.99 |
| PAI-1 4G/5G heterozygous | PAI-1 4G/5G homozygous | > 0.99 |

APC, activated protein C; MTHFR, methylenetetrahydrofolate reductase; PAI-1, plasminogen activator inhibitor-1.

^a^ *P* values were adjusted for multiple comparisons using the Holm method.
