# Supplementary material for: Recurrent pregnancy loss: systematic review and meta-analysis of overall prevalence and the distribution of major etiological categories
Source: Front Med (Lausanne). 2026 Apr 1;13:1805994. doi: 10.3389/fmed.2026.1805994 (PMC13079578; doi:10.3389/fmed.2026.1805994)
Supplement: Supplementary file 2 [file Data_sheet_2.zip › Supplementary Tables/SuppTable05.docx]

**Supplementary Table 5.** Pairwise comparisons of anatomical factors.

| Subgroup 1 | Subgroup 2 | *P* value^a^ |
| --- | --- | --- |
| Congenital uterine anomalies | Acquired uterine anomalies | 0.63 |
| Congenital uterine anomalies | Unspecified uterine anomalies | 0.63 |
| Congenital uterine anomalies | Cervical insufficiency | 0.25 |
| Acquired uterine anomalies | Unspecified uterine anomalies | 0.63 |
| Acquired uterine anomalies | Cervical insufficiency | 0.03 |
| Unspecified uterine anomalies | Cervical insufficiency | 0.03 |

^a^ *P* values were adjusted for multiple comparisons using the Holm method.
