# Supplementary material for: Recurrent pregnancy loss: systematic review and meta-analysis of overall prevalence and the distribution of major etiological categories
Source: Front Med (Lausanne). 2026 Apr 1;13:1805994. doi: 10.3389/fmed.2026.1805994 (PMC13079578; doi:10.3389/fmed.2026.1805994)
Supplement: Supplementary file 2 [file Data_sheet_2.zip › Supplementary Tables/SuppTable10.docx]

**Supplementary Table 10.** Pairwise comparisons of the estimated distribution of each major etiological category of recurrent pregnancy loss between continents.

| **Subgroup 1** | **Subgroup 2** | **Difference** | **SE** | **Z-value** | ***P* value** | ***P* value^a^** |
| --- | --- | --- | --- | --- | --- | --- |
| **Acquired thrombophilia** |  |  |  |  |  |  |
| Asia | North America | -0.070 | 0.38 | -0.75 | 0.45 | > 0.99 |
| Asia | South America | -0.033 | 0.39 | -0.34 | 0.74 | > 0.99 |
| Asia | Europe | 0.083 | 0.34 | 0.99 | 0.32 | > 0.99 |
| Asia | Oceania | -0.039 | 0.36 | -0.43 | 0.67 | > 0.99 |
| North America | South America | 0.037 | 0.36 | 0.42 | 0.67 | > 0.99 |
| North America | Europe | 0.150 | 0.30 | 2.05 | 0.040 | 0.40 |
| North America | Oceania | 0.031 | 0.32 | 0.39 | 0.70 | > 0.99 |
| South America | Europe | 0.115 | 0.31 | 1.48 | 0.14 | > 0.99 |
| South America | Oceania | -0.006 | 0.34 | -0.07 | 0.94 | > 0.99 |
| Europe | Oceania | -0.121 | 0.28 | -1.76 | 0.078 | 0.07 |
| **Hereditary thrombophilia** |  |  |  |  |  |  |
| Asia | Europe | 0.034 | 0.25 | 0.55 | 0.58 | > 0.99 |
| Asia | South America | -0.043 | 0.38 | -0.46 | 0.65 | > 0.99 |
| Asia | North America | -0.023 | 0.52 | -0.18 | 0.86 | > 0.99 |
| Asia | Africa | 0.368 | 0.45 | 4.18 | < 0.0001 | < 0.001 |
| Europe | South America | -0.077 | 0.35 | -0.89 | 0.37 | > 0.99 |
| Europe | North America | -0.058 | 0.50 | -0.46 | 0.64 | > 0.99 |
| Europe | Africa | 0.351 | 0.43 | 4.10 | < 0.0001 | < 0.001 |
| South America | North America | 0.020 | 0.57 | 0.14 | 0.89 | > 0.99 |
| South America | Africa | 0.387 | 0.51 | 4.02 | < 0.0001 | < 0.001 |
| North America | Africa | 0.378 | 0.62 | 3.17 | 0.002 | 0.01 |
| **Anatomical factors** |  |  |  |  |  |  |
| Asia | North America | -0.166 | 0.27 | -2.57 | 0.01 | 0.05 |
| Asia | Europe | -0.095 | 0.31 | -1.26 | 0.21 | 0.63 |
| Asia | South America | -0.329 | 0.31 | -5.09 | < 0.0001 | < 0.0001 |
| Asia | Africa | 0.077 | 0.29 | 1.08 | 0.28 | 0.63 |
| North America | Europe | 0.075 | 0.33 | 0.91 | 0.36 | 0.63 |
| North America | South America | -0.209 | 0.34 | -2.64 | 0.01 | 0.05 |
| North America | Africa | 0.231 | 0.32 | 3.14 | 0.002 | 0.01 |
| Europe | South America | -0.267 | 0.37 | -3.24 | 0.001 | 0.01 |
| Europe | Africa | 0.167 | 0.35 | 1.99 | 0.047 | 0.19 |
| South America | Africa | 0.369 | 0.35 | 5.34 | < 0.0001 | < 0.0001 |
| **Endocrine factors** |  |  |  |  |  |  |
| Asia | South America | -0.307 | 0.40 | -2.03 | 0.042 | 0.29 |
| Asia | Europe | 0.043 | 0.33 | 0.52 | 0.60 | > 0.99 |
| Asia | North America | 0.059 | 0.37 | 0.65 | 0.52 | > 0.99 |
| Asia | Africa | 0.255 | 0.82 | 1.37 | 0.17 | > 0.99 |
| South America | Europe | 0.332 | 0.73 | 2.20 | 0.028 | 0.23 |
| South America | North America | -0.341 | 0.74 | 2.24 | 0.025 | 0.23 |
| South America | Africa | 0.428 | 1.04 | 2.45 | 0.02 | 0.15 |
| Europe | North America | 0.016 | 0.41 | 0.16 | 0.88 | > 0.99 |
| Europe | Africa | 0.216 | 0.84 | 1.13 | 0.26 | > 0.99 |
| North America | Africa | 0.208 | 0.85 | 1.04 | 0.30 | > 0.99 |
| **Parental chromosomal abnormalities** |  |  |  |  |  |  |
| Europe | Asia | -0.081 | 0.30 | -1.09 | 0.28 | 0.56 |
| Europe | North America | 0.058 | 0.35 | 0.66 | 0.51 | 0.56 |
| Europe | South America | -0.259 | 0.57 | -2.02 | 0.04 | 0.22 |
| Asia | North America | 0.137 | 0.31 | 1.79 | 0.07 | 0.29 |
| Asia | South America | -0.194 | 0.54 | -1.50 | 0.13 | 0.40 |
| North America | South America | -0.299 | 0.57 | -2.41 | 0.02 | 0.10 |
| **Infectious causes** |  |  |  |  |  |  |
| Asia | North America | -0.137 | 0.84 | -0.67 | 0.50 | 0.72 |
| Asia | Europe | 0.201 | 0.94 | 0.91 | 0.36 | 0.72 |
| North America | Europe | 0.305 | 1.04 | 1.36 | 0.17 | 0.52 |
| **Idiopathic RPL** |  |  |  |  |  |  |
| Asia | Europe | -0.036 | 0.23 | -0.62 | 0.53 | > 0.99 |
| Asia | North America | 0.199 | 1.04 | 0.81 | 0.42 | > 0.99 |
| Europe | North America | 0.229 | 1.02 | 0.96 | 0.34 | > 0.99 |
|  |  |  |  |  |  |  |

RPL, recurrent pregnancy loss; SE, standard error.

^a^ *P* values were adjusted for multiple comparisons using the Holm method.
