# Supplementary material for: Recurrent pregnancy loss: systematic review and meta-analysis of overall prevalence and the distribution of major etiological categories
Source: Front Med (Lausanne). 2026 Apr 1;13:1805994. doi: 10.3389/fmed.2026.1805994 (PMC13079578; doi:10.3389/fmed.2026.1805994)
Supplement: Supplementary file 2 [file Data_sheet_2.zip › Supplementary Tables/SuppTable11.docx]

**Supplementary Table 11.** Meta-regression for each major etiological category of recurrent pregnancy loss and mean age.

| Cause of RPL | Slope | Lower 95% CI | Upper 95% CI | *P* value |
| --- | --- | --- | --- | --- |
| Acquired thrombophilia | -0.053 | -0.141 | 0.036 | 0.24 |
| Hereditary thrombophilia | -0.03 | -0.104 | 0.044 | 0.42 |
| Anatomical factors | 0.066 | -0.021 | 0.153 | 0.14 |
| Endocrine factors | 0.026 | -0.076 | 0.128 | 0.61 |
| Parental chromosomal abnormalities | -0.016 | -0.126 | 0.093 | 0.77 |
| Infectious causes | 0.129 | -0.164 | 0.422 | 0.39 |
| Idiopathic RPL | -0.033 | -0.169 | 0.104 | 0.64 |

CI, confidence interval; RPL, recurrent pregnancy loss.
