# Supplementary material for: Recurrent pregnancy loss: systematic review and meta-analysis of overall prevalence and the distribution of major etiological categories
Source: Front Med (Lausanne). 2026 Apr 1;13:1805994. doi: 10.3389/fmed.2026.1805994 (PMC13079578; doi:10.3389/fmed.2026.1805994)
Supplement: Supplementary file 2 [file Data_sheet_2.zip › Supplementary Tables/SuppTable12.docx]

**Supplementary Table 12.** Meta-regression for each major etiological category of recurrent pregnancy loss and mean body mass index.

| Cause of RPL | Slope | Lower 95% CI | Upper 95% CI | *P* value |
| --- | --- | --- | --- | --- |
| Acquired thrombophilia | 0.168 | 0.004 | 0.332 | 0.045 |
| Hereditary thrombophilia | 0.269 | -0.089 | 0.627 | 0.14 |
| Anatomical factors | 0.044 | -0.281 | 0.369 | 0.79 |
| Endocrine factors | -0.133 | -0.505 | 0.24 | 0.48 |
| Parental chromosomal abnormalities | 0.001 | -0.142 | 0.144 | 0.99 |

CI, confidence interval; RPL, recurrent pregnancy loss.
