# Supplementary material for: Recurrent pregnancy loss: systematic review and meta-analysis of overall prevalence and the distribution of major etiological categories
Source: Front Med (Lausanne). 2026 Apr 1;13:1805994. doi: 10.3389/fmed.2026.1805994 (PMC13079578; doi:10.3389/fmed.2026.1805994)
Supplement: Supplementary file 2 [file Data_sheet_2.zip › Supplementary Tables/SuppTable13.docx]

**Supplementary Table 13.** Meta-regression for each major etiological category of recurrent pregnancy loss and year of publication.

| Cause of RPL | Slope | Lower 95% CI | Upper 95% CI | *P* value |
| --- | --- | --- | --- | --- |
| Acquired thrombophilia | -0.046 | -0.069 | -0.022 | 0.0001 |
| Hereditary thrombophilia | 0.012 | -0.016 | 0.04 | 0.41 |
| Anatomical factors | -0.031 | -0.057 | -0.006 | 0.02 |
| Endocrine factors | -0.007 | -0.039 | 0.026 | 0.70 |
| Parental chromosomal abnormalities | 0.003 | -0.028 | 0.034 | 0.85 |
| Infectious causes | 0.043 | -0.019 | 0.106 | 0.17 |
| Idiopathic RPL | -0.006 | -0.042 | 0.030 | 0.75 |

CI, confidence interval; RPL, recurrent pregnancy loss.
