# Supplementary material for: Recurrent pregnancy loss: systematic review and meta-analysis of overall prevalence and the distribution of major etiological categories
Source: Front Med (Lausanne). 2026 Apr 1;13:1805994. doi: 10.3389/fmed.2026.1805994 (PMC13079578; doi:10.3389/fmed.2026.1805994)
Supplement: Supplementary file 2 [file Data_sheet_2.zip › Supplementary Tables/SuppTable6.docx]

**Supplementary Table 6.** Pairwise comparisons for endocrine factors.

| Subgroup 1 | Subgroup 2 | P value^a^ |
| --- | --- | --- |
| Abnormal thyroid autoantibodies | Subclinical hypothyroidism | > 0.99 |
| Abnormal thyroid autoantibodies | Hypothyroidism | > 0.99 |
| Abnormal thyroid autoantibodies | Hyperthyroidism | < 0.001 |
| Abnormal thyroid autoantibodies | Unspecified abnormal thyroid function | > 0.99 |
| Abnormal thyroid autoantibodies | Glucose metabolism disorders | > 0.99 |
| Abnormal thyroid autoantibodies | Hyperprolactinemia | > 0.99 |
| Abnormal thyroid autoantibodies | Luteal phase defect | > 0.99 |
| Abnormal thyroid autoantibodies | Polycystic ovary syndrome | > 0.99 |
| Subclinical hypothyroidism | Hypothyroidism | > 0.99 |
| Subclinical hypothyroidism | Hyperthyroidism | 0.11 |
| Subclinical hypothyroidism | Unspecified abnormal thyroid function | > 0.99 |
| Subclinical hypothyroidism | Glucose metabolism disorders | > 0.99 |
| Subclinical hypothyroidism | Hyperprolactinemia | > 0.99 |
| Subclinical hypothyroidism | Luteal phase defect | 0.18 |
| Subclinical hypothyroidism | Polycystic ovary syndrome | > 0.99 |
| Hypothyroidism | Hyperthyroidism | 0.05 |
| Hypothyroidism | Unspecified abnormal thyroid function | > 0.99 |
| Hypothyroidism | Glucose metabolism disorders | > 0.99 |
| Hypothyroidism | Hyperprolactinemia | > 0.99 |
| Hypothyroidism | Luteal phase defect | 0.02 |
| Hypothyroidism | Polycystic ovary syndrome | > 0.99 |
| Hyperthyroidism | Unspecified abnormal thyroid function | < 0.001 |
| Hyperthyroidism | Glucose metabolism disorders | 0.06 |
| Hyperthyroidism | Hyperprolactinemia | 0.02 |
| Hyperthyroidism | Luteal phase defect | < 0.001 |
| Hyperthyroidism | Polycystic ovary syndrome | 0.007 |
| Unspecified abnormal thyroid function | Glucose metabolism disorders | > 0.99 |
| Unspecified abnormal thyroid function | Hyperprolactinemia | > 0.99 |
| Unspecified abnormal thyroid function | Luteal phase defect | 0.84 |
| Unspecified abnormal thyroid function | Polycystic ovary syndrome | > 0.99 |
| Glucose metabolism disorders | Hyperprolactinemia | > 0.99 |
| Glucose metabolism disorders | Luteal phase defect | 0.03 |
| Glucose metabolism disorders | Polycystic ovary syndrome | > 0.99 |
| Hyperprolactinemia | Luteal phase defect | 0.09 |
| Hyperprolactinemia | Polycystic ovary syndrome | > 0.99 |
| Luteal phase defect | Polycystic ovary syndrome | 0.23 |

^a^ *P* values were adjusted for multiple comparisons using the Holm method.
