# Supplementary material for: Recurrent pregnancy loss: systematic review and meta-analysis of overall prevalence and the distribution of major etiological categories
Source: Front Med (Lausanne). 2026 Apr 1;13:1805994. doi: 10.3389/fmed.2026.1805994 (PMC13079578; doi:10.3389/fmed.2026.1805994)
Supplement: Supplementary file 2 [file Data_sheet_2.zip › Supplementary Tables/SuppTable8.docx]

**Supplementary Table 8.** Subgroup analyses of the estimated distribution of each major etiological category of recurrent pregnancy loss, by continent.

| Cause of RPL | Q | df | *P* value |
| --- | --- | --- | --- |
| Acquired thrombophilia | 38.7 | 5 | < 0.0001 |
| Hereditary thrombophilia | 20.46 | 4 | < 0.001 |
| Anatomical factors | 36.71 | 4 | < 0.0001 |
| Endocrine factors | 7.24 | 4 | 0.12 |
| Parental chromosomal abnormalities | 10.50 | 5 | 0.06 |
| Infectious causes | 3.24 | 3 | 0.36 |
| Idiopathic RPL | 121.18 | 5 | < 0.0001 |

RPL, recurrent pregnancy loss.
