# Supplementary material for: Recurrent pregnancy loss: systematic review and meta-analysis of overall prevalence and the distribution of major etiological categories
Source: Front Med (Lausanne). 2026 Apr 1;13:1805994. doi: 10.3389/fmed.2026.1805994 (PMC13079578; doi:10.3389/fmed.2026.1805994)
Supplement: Supplementary file 2 [file Data_sheet_2.zip › Supplementary Tables/SuppTable9.docx]

**Supplementary Table 9.** Estimated distribution of each major etiological category of recurrent pregnancy loss, by continent.

| **Subgroup** | **Estimate**  **(95% CI)** | **I²**  **(%)** | **Studies**  **(n)** |
| --- | --- | --- | --- |
| **Acquired thrombophilia** |  |  |  |
| Asia | 0.123 (0.074 to 0.198) | 0.96 | 26 |
| North America | 0.157 (0.104 to 0.230) | 0.97 | 11 |
| South America | 0.138 (0.088 to 0.210) | 0.26 | 3 |
| Europe | 0.091 (0.066 to 0.125) | 0.93 | 18 |
| Oceania | 0.141 (0.097 to 0.200) | 0.06 | 2 |
| **Hereditary thrombophilia** |  |  |  |
| Asia | 0.068 (0.047 to 0.099) | 0.96 | 46 |
| Europe | 0.060 (0.046 to 0.078) | 0.95 | 86 |
| South America | 0.080 (0.045 to 0.140) | 0.92 | 19 |
| North America | 0.075 (0.031 to 0.170) | 0.97 | 14 |
| Africa | 0.011 (0.005 to 0.024) | 0.46 | 2 |
| **Anatomical factors** |  |  |  |
| Asia | 0.047 (0.034 to 0.064) | 0.91 | 34 |
| North America | 0.089 (0.061 to 0.129) | 0.87 | 11 |
| Europe | 0.068 (0.042 to 0.107) | 0.94 | 26 |
| South America | 0.193 (0.125 to 0.286) | 0.63 | 4 |
| Africa | 0.035 (0.022 to 0.054) | 0.67 | 3 |
| **Endocrine factors** |  |  |  |
| Asia | 0.089 (0.063 to 0.125) | 0.96 | 47 |
| South America | 0.290 (0.098 to 0.606) | 0.90 | 2 |
| Europe | 0.076 (0.046 to 0.123) | 0.95 | 24 |
| North America | 0.072 (0.040 to 0.125) | 0.95 | 19 |
| Africa | 0.031 (0.007 to 0.132) | 0.97 | 5 |
| **Parental chromosomal abnormalities** |  |  |  |
| Europe | 0.040 (0.025 to 0.063) | 0.91 | 15 |
| Asia | 0.055 (0.039 to 0.076) | 0.95 | 23 |
| North America | 0.032 (0.020 to 0.052) | 0.59 | 6 |
| South America | 0.116 (0.046 to 0.265) | 0.92 | 3 |
| **Infectious causes** |  |  |  |
| Asia | 0.053 (0.020 to 0.129) | 0.92 | 8 |
| North America | 0.089 (0.025 to 0.265) | 0.82 | 5 |
| Europe | 0.023 (0.005 to 0.100) | 0.91 | 4 |
| **Idiopathic RPL** |  |  |  |
| Asia | 0.412 (0.318 to 0.513) | 0.95 | 8 |
| Europe | 0.448 (0.397 to 0.501) | 0.70 | 6 |
| North America | 0.232 (0.039 to 0.690) | 0.87 | 2 |

CI, confidence interval; RPL, recurrent pregnancy loss.
